# DDX5 inhibits hyaline cartilage fibrosis and degradation in osteoarthritis via alternative splicing and G-quadruplex unwinding

**DOI:** 10.1038/s43587-024-00624-0

**Published:** 2024-05-17

**Authors:** Qianqian Liu, Mingrui Han, Zhigui Wu, Wenqiang Fu, Jun Ji, Qingqing Liang, Minjia Tan, Linhui Zhai, Jian Gao, Dongquan Shi, Qing Jiang, Ziying Sun, Yuping Lai, Qiang Xu, Yang Sun

**Affiliations:** 1 State Key Laboratory of Pharmaceutical Biotechnology and Department of Sports Medicine and Adult Reconstructive Surgery, Nanjing Drum Tower Hospital, the Affiliated Hospital of Nanjing University Medical School, School of Life Sciences, Nanjing University, Nanjing, China; 2 Jiangsu Key Laboratory of New Drug Research and Clinical Pharmacy, Xuzhou Medical University, Xuzhou, China; 3 Peking-Tsinghua Center for Life Sciences, Academy for Advanced Interdisciplinary Studies, Peking University, Beijing, China; 4 High Magnetic Field Laboratory, Chinese Academy of Sciences, Hefei, China; 5 Nanjing Stomatological Hospital, Affiliated Hospital of Medical School, Nanjing University, Nanjing, China; 6 State Key Laboratory of Drug Research, Shanghai Institute of Materia Medica, Chinese Academy of Sciences, Shanghai, China; 7 Department of Sports Medicine and Adult Reconstructive Surgery, Nanjing Drum Tower Hospital, The Affiliated Hospital of Nanjing University Medical School, Nanjing, China; 8 Department of Orthopaedics, Jinling Hospital, The Affiliated Hospital of Nanjing University Medical School, Nanjing, China; 9 Shanghai Key Laboratory of Regulatory Biology, School of Life Sciences, East China Normal University, Shanghai, China

**Keywords:** RNA splicing, Proteomics, Solution-state NMR, Ageing

## Abstract

Hyaline cartilage fibrosis is typically considered an end-stage pathology of osteoarthritis (OA), which results in changes to the extracellular matrix. However, the mechanism behind this is largely unclear. Here, we found that the RNA helicase DDX5 was dramatically downregulated during the progression of OA. DDX5 deficiency increased fibrosis phenotype by upregulating COL1 expression and downregulating COL2 expression. In addition, loss of DDX5 aggravated cartilage degradation by inducing the production of cartilage-degrading enzymes. Chondrocyte-specific deletion of *Ddx5* led to more severe cartilage lesions in the mouse OA model. Mechanistically, weakened DDX5 resulted in abundance of the *Fn1*-AS-WT and *Plod2*-AS-WT transcripts, which promoted expression of fibrosis-related genes (*Col1*, *Acta2*) and extracellular matrix degradation genes (*Mmp13*, *Nos2* and so on), respectively. Additionally, loss of DDX5 prevented the unfolding *Col2* promoter G-quadruplex, thereby reducing COL2 production. Together, our data suggest that strategies aimed at the upregulation of DDX5 hold significant potential for the treatment of cartilage fibrosis and degradation in OA.

## Abstract (web summary)

Liu et al. identify downregulation of DDX5, an RNA helicase, in the cartilage of patients and mouse model of osteoarthritis. Targeted upregulation of DDX5 in mouse chondrocytes inhibits hyaline cartilage fibrosis and degradation via pre-mRNA splicing and G4 unwinding, a potential therapeutic strategy against osteoarthritis.

## Main

Healthy articular cartilage has a smooth extracellular matrix (ECM) composed mostly of aggrecan and type II collagen (COL2), with chondrocytes remaining in small spaces called lacunae^1^. Once cartilage is damaged, chondrocytes undergo abnormal proliferation. Eventually, proliferative chondrocytes change into a fibroblast-like phenotype, forming a thick layer of fibrocartilage-like tissue by synthesizing type I collagen (COL1) and inducing degradation and de-differentiation in the nearby hyaline cartilage^1,2^. These changes result in stiffer, less durable cartilage and eventually transform the articular cartilage phenotype, fueling osteoarthritis (OA) progression^3,4^. Increasing evidence acquired from single-cell sequencing efforts demonstrated that fibrocartilage chondrocytes have an important role in OA progression^5^. Thus, the fibrocartilage produced after cartilage injury cannot be ignored. However, the mechanism of hyaline cartilage fibrosis is largely unclear.

Alternative splicing (AS) is a crucial biological process that enhances the coding capacity of a finite genome^6^. An increasing number of examples illustrate that abnormal AS events can lead to human diseases such as cancer^7,8^. Some genes, such as procollagen-lysine,2-oxoglutarate 5-dioxygenase 2 (*PLOD2*), fibronectin 1 (*FN1*) and transforming growth factor beta receptor 1 (*TGFBR1*), which regulate ECM-related pathways, have many AS events^9,10^. However, it is unclear whether aberrant AS events are involved in hyaline cartilage fibrosis.

Our study shows that DDX5, one of the most important members of the DEAD-box RNA helicase family, may have a dual role in preventing both cartilage degradation and the development of the fibrocartilage phenotype. DDX5 prevents the degradation of hyaline cartilage and fibrosis by regulating the splicing of *Fn1* and *Plod2* pre-mRNAs, as well as unwinding the G-quadruplex (G4) of the *Col2* promoter. These findings suggest that DDX5 has the potential to be a therapeutic target for the treatment of OA.

## Results

### Identification of DDX5 as a downregulated gene and protein in relation to OA progression in human patient samples and animal models

**Fig. 1:**
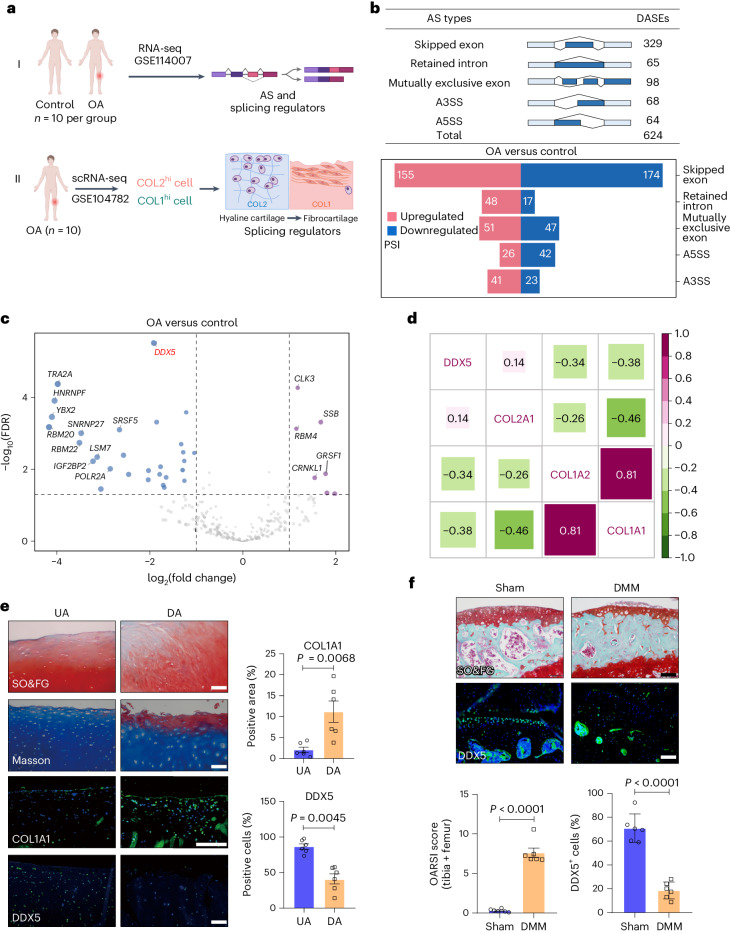
Identification of *DDX5* as a downregulated gene and protein in relation to OA progression in human patient samples and animal models. **a**, Schematic of the experimental design. **b**, rMATS analysis of the number of DASEs between healthy and OA samples (*n* = 10 per group). Delta PSI > 4%, FDR < 0.05. **c**, Differential expression analysis in OA versus healthy (FDR < 0.05) **d**, Analysis of the correlation between DDX5 and COL1 and COL2 in COL2^hi^ and COL1^hi^ cells. Correlation coefficients are represented by numbers. **e**, Safranin O/Fast Green (SO&FG) and Masson staining, and immunofluorescence (IF) detection, of COL1A1 and DDX5 in OA cartilage (left). Quantitation of COL1A1 and DDX5 (*n* = 6 biologically independent samples). **f**, IF of DDX5 and SO&FG staining 12 week after mouse surgery for destabilization of the medial meniscus (DMM). Quantification of the Osteoarthritis Research Society International (OARSI) score and DDX5 protein (*n* = 6 mice per group). **e**,**f**, Scale bar, 100 µm. All data are presented as the mean ± s.e.m. A Student’s *t*-test (paired in **e**, unpaired in **f**) were conducted. Source data

**Extended Data Fig. 1:**
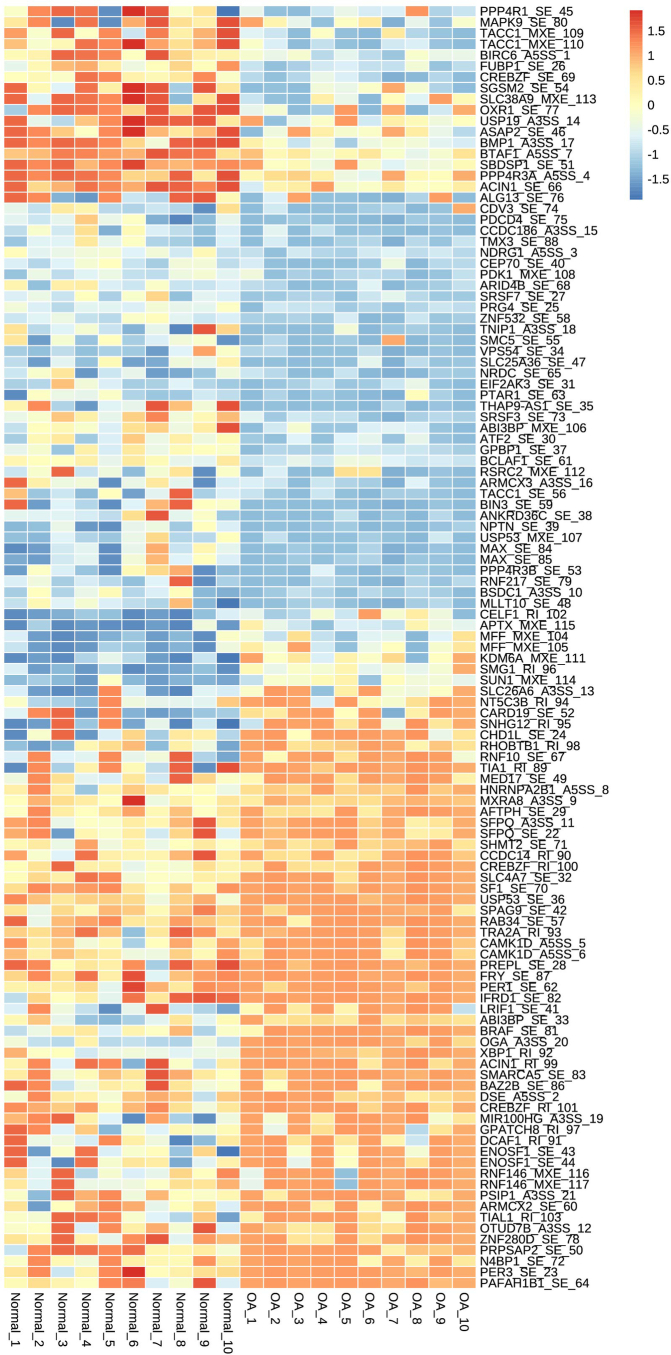
The most significant differential alternative splicing events in OA. The most significant differential alternative splicing events between normal and osteoarthritis (OA) samples (RNA^−^seq datasets GSE114007, n = 10 /group) were analyzed using rMATS. The screening cut off delta PSI > 20% and FDR < 0.0001.The schematic diagram was shown in Panel a-I of Fig. 1.

**Extended Data Fig. 2:**
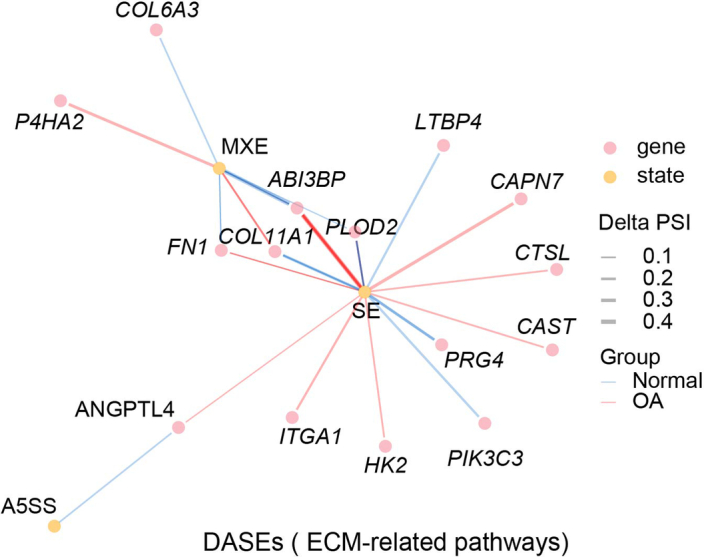
The differential alternative splicing events of ECM-related pathways in OA. The DASEs between normal and OA samples (GSE114007, n = 10 /group) were analyzed using rMATS. The screening cut off delta PSI > 4% and FDR < 0.05. The schematic diagram was shown in Panel a-I of Fig. 1.

**Extended Data Fig. 3:**
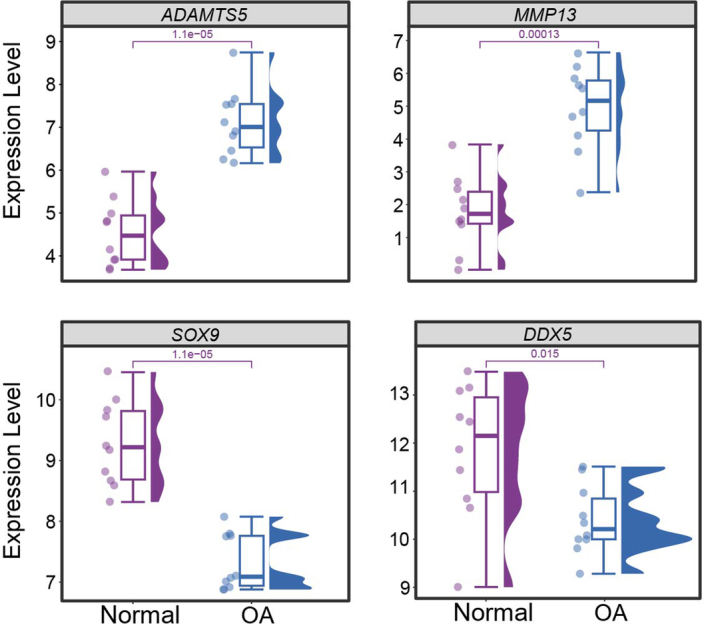
The expression levels of the representative candidate marker genes and DDX5 between normal and OA samples. The schematic diagram for this panel was shown in Panel a-I of Fig. 1 (GSE114007, n = 10 samples/group). The significant differences were tested in related genes through ‘wilcox test’. The box plots was defined as follows: minima (Q1 - 1.5×IQR), maxima (Q3 + 1.5×IQR), centre (Q2), bounds of box (Q1 - Q3) and whiskers (Min-Max) and percentile (Min, 0%; Q1, 25% percentile; Q2, 50% percentile; Q3, 75% percentile, Max, 100%).

**Extended Data Fig. 4:**
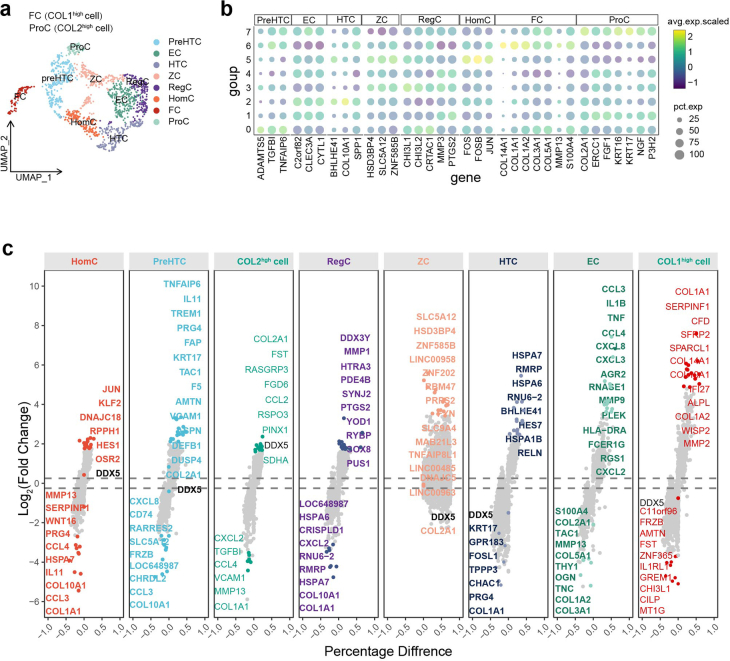
Identification of chondrocyte populations and gene signatures during human OA progression. (**a**) Single-cell count matrices were obtained from the GEO database (GSE104782). The UMAP plot of all cells were colored by their cell type identity. The cell types classified are as follows: PreHTC (prehypertrophic chondrocyte), EC (effector chondrocyte), HTC (hypertrophic chondrocyte), ZC (ZNF585B positive chondrocyte), RegC (regulatory chondrocyte), HomC (homeostatic chondrocyte), FC (fibrocartilage chondrocyte), ProC (proliferative chondrocyte). (**b**) The bubble chart displays some characteristic genes in different clusters. (**c**) The dot plot represents the differential genes expression (cell cluster in each group vs all other cell clusters).

**Extended Data Fig. 5:**
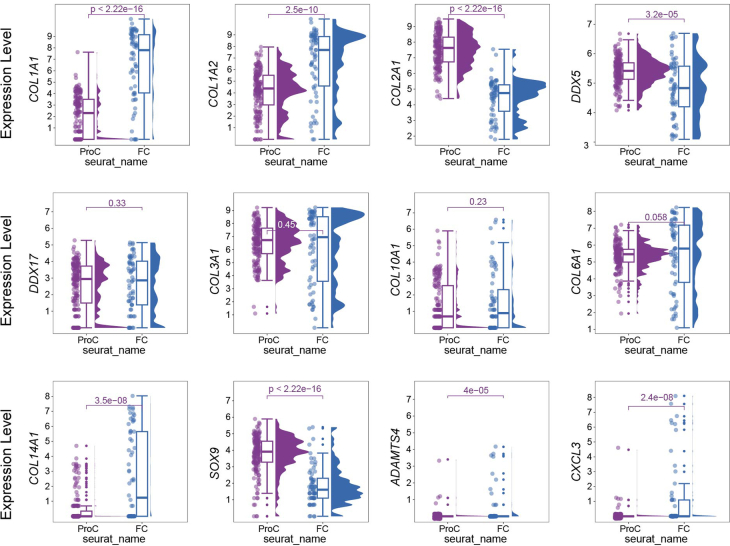
The expression of related genes in COL1^high^ cells and COL2^high^ cells. The significant differences were tested in related genes through ‘wilcox test’ (GSE104782, n = 10 samples/group). The box plots was defined as follows: minima (Q1 - 1.5×IQR), maxima (Q3 + 1.5×IQR), centre (Q2), bounds of box (Q1 - Q3) and whiskers (Min-Max) and percentile (Min, 0%; Q1, 25% percentile; Q2, 50% percentile; Q3, 75% percentile, Max, 100%).

To investigate whether AS is involved in OA pathogenesis, we depicted the AS landscape in cartilage tissues from patients with OA and donors without OA using the RNA sequencing (RNA-seq) datasets (GSE114007)^11^ (Fig. 1a-I). We identified a total of 624 differential AS events (DASEs) in the group with OA compared to donors without OA, which can be categorized into five major AS patterns, including skipped exon, exon exclusion, retained intron and alternative 3′­ and 5′­ splicing (A3SS and A5SS) (Fig. 1b). One hundred and seventeen significant DASEs were identified with delta percentage spliced in (PSI) > 20% and a false discovery rate (FDR) < 0.0001 (Extended Data Fig. 1). These AS gene changes related to ECM pathways were indicated (Extended Data Fig. 2). With the exception of *HK2* and *ANGPTL4*, most of the differentially expressed genes were also identified in the analysis by Li et al.^12^. Then, to identify dysregulated splicing regulators related to hyaline cartilage fibrosis, we aggregated a comprehensive list of 452 splicing regulators from different sources, including previous publications^13–17^ and the SpliceAid2 database^18^. Thirty-six genes encoding splicing regulators were dysregulated in the group with OA compared to the control group; DDX5 ranked first according to its FDR and was downregulated (Fig. 1c and Extended Data Fig. 3). To further determine the intrinsic association of DDX5 with hyaline cartilage fibrosis, we analyzed the single-cell RNA-seq (scRNA-seq) dataset (GSE104782; Fig. 1a-II) of joint cartilage from ten patients with OA undergoing knee arthroplasty^19^. We defined two populations: a population of proliferative chondrocytes (COL2^hi^ cells) and a population of fibrocartilage chondrocytes (COL1^hi^ cells) (Extended Data Fig. 4). Further analysis of these two populations revealed that DDX5 expression was higher in COL2^hi^ cells than in COL1^hi^ cells (Extended Data Fig. 5). However, the expression of *DDX17*, a homolog of *DDX5*, did not differ significantly in COL2^hi^ and COL1^hi^ cells. Also, chondrocyte hypertrophic markers of type X collagen (COL X) did not show a significant difference between the two groups, while chondrogenic markers, including transcription factors SOX-9 (*SOX9*) and COL2, had lower expression levels in COL1^hi^ cells compared to COL2^hi^ cells (Extended Data Fig. 5). In addition, expression of DDX5 showed a negative correlation with COL1, whereas it showed positive correlation with COL2 (Fig. 1d).

**Extended Data Fig. 6:**
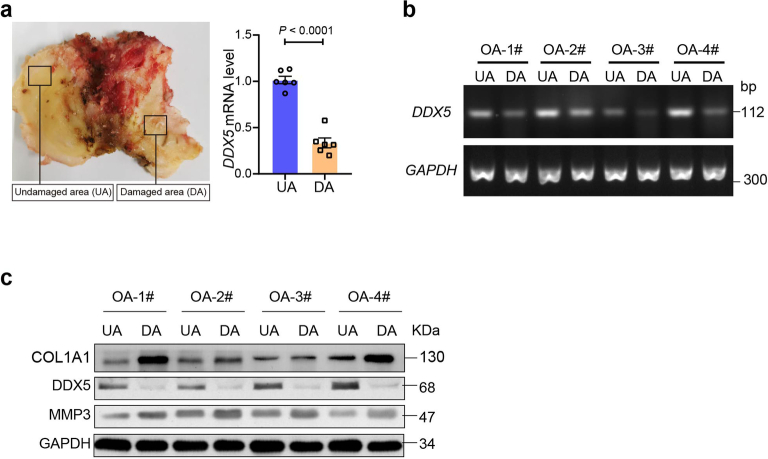
The expression level of DDX5 in damaged areas and uninjured areas of cartilage from OA patients. (**a**) QPCR was used to detect the mRNA level of DDX5 in damaged areas (DA) and uninjured areas (UA) of cartilage from OA patients (n = 6 samples). The data are presented as the mean ± SEM. Student’s *t*-test (paired) was conducted. (**b**) The mRNA expression of *DDX5* in UA and DA of human OA cartilage was detected by RT-PCR analysis (n = 4 samples). (**c**) Western bolt analysis was performed to detect the expression of DDX5, COL1A1 and MMP3 in the DA and UA of cartilage from OA patients (n = 4 samples). Source data

**Extended Data Fig. 7:**
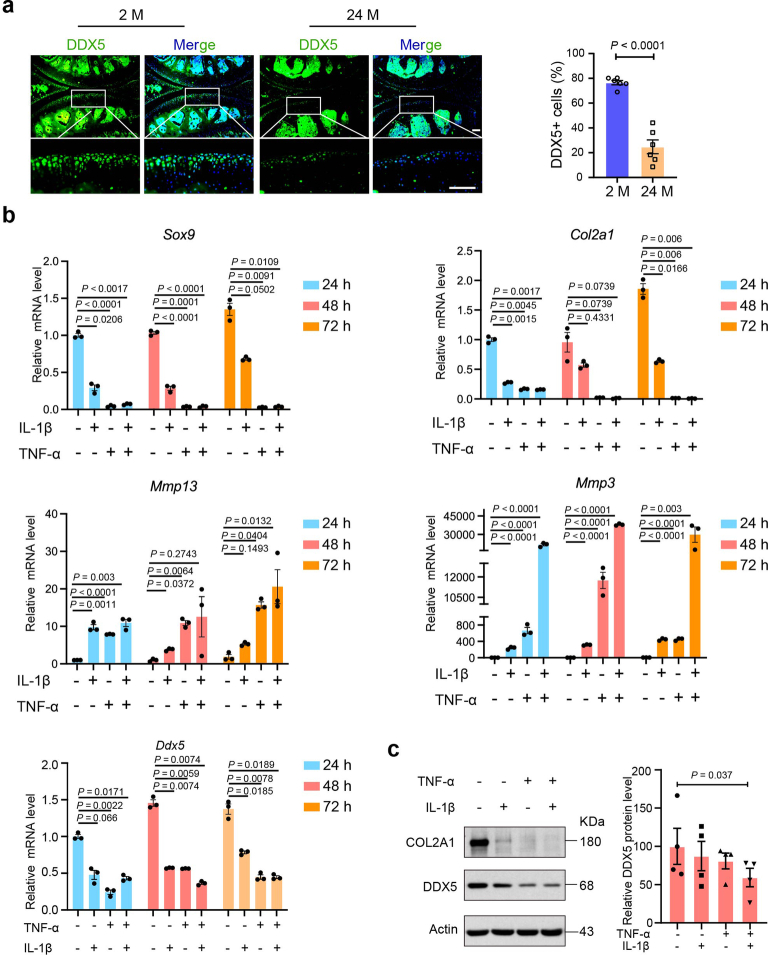
The expression level of DDX5 in aged mice and under cytokine stimulation conditions. Related to Fig. 1. (**a**) Representative images of DDX5 immunostaining in 2-month-old and 24-month-old mice (left panel). Scale bars, 100 μm. Quantitative of DDX5 (right panel, n = 6 mice/group). (**b**) QPCR analysis of *Sox9*, *Col2a1*, *Mmp3* and *Ddx5* mRNA levels in primary mouse chondrocytes stimulated with 5 ng/ml IL-1β and/or 25 ng/ml TNF-α for 12 h. (**c**) Western bolt analysis of DDX5 in primary mouse chondrocytes stimulated with 5 ng/ml IL-1β and/or 25 ng/ml TNF-α for 48 h (n = 4 independent experiments). COL2A1, as a positive control. All data are presented as the mean ± SEM. Unpaired Student’s *t*-test (a), two-way ANOVA with Tukey’s multiple comparisons test (b) and Friedman test (c) were conducted. Source data

Then, we collected cartilage samples from patients with OA undergoing joint replacement surgery, both from the undamaged (UA) and damaged (DA) areas (Extended Data Fig. 6a). Fibrocartilage tissue was more easily detected in DAs, with a more fibrous matrix, and was accompanied by a high expression of COL1 (Fig. 1e). In addition, a significant decrease in both the mRNA and protein expression levels of DDX5 was found in DAs compared to UAs (Fig. 1e and Extended Data Fig. 6). Furthermore, expression of DDX5 in the articular cartilage of the OA mouse model was significantly lower than that of sham-operated mice (Fig. 1f). Similarly, expression of DDX5 in the knee articular cartilage of aged mice was significantly lower than that in young mice (Extended Data Fig. 7a). Pro-inflammatory cytokines, such as interleukin-1β (IL-1β) and tumor necrosis factor-α (TNF-α), are critical mediators of the disturbed processes implicated in OA pathophysiology. We combined IL-1β and TNF-α to stimulate primary mouse chondrocytes in vitro and found that the mRNA and protein levels of DDX5 were also significantly decreased (Extended Data Fig. 7b,c). These data suggest that DDX5 may have an important role during the progression of hyaline cartilage fibrosis.

### Regulation of DDX5 expression alters the progression of posttraumatic OA

**Fig. 2:**
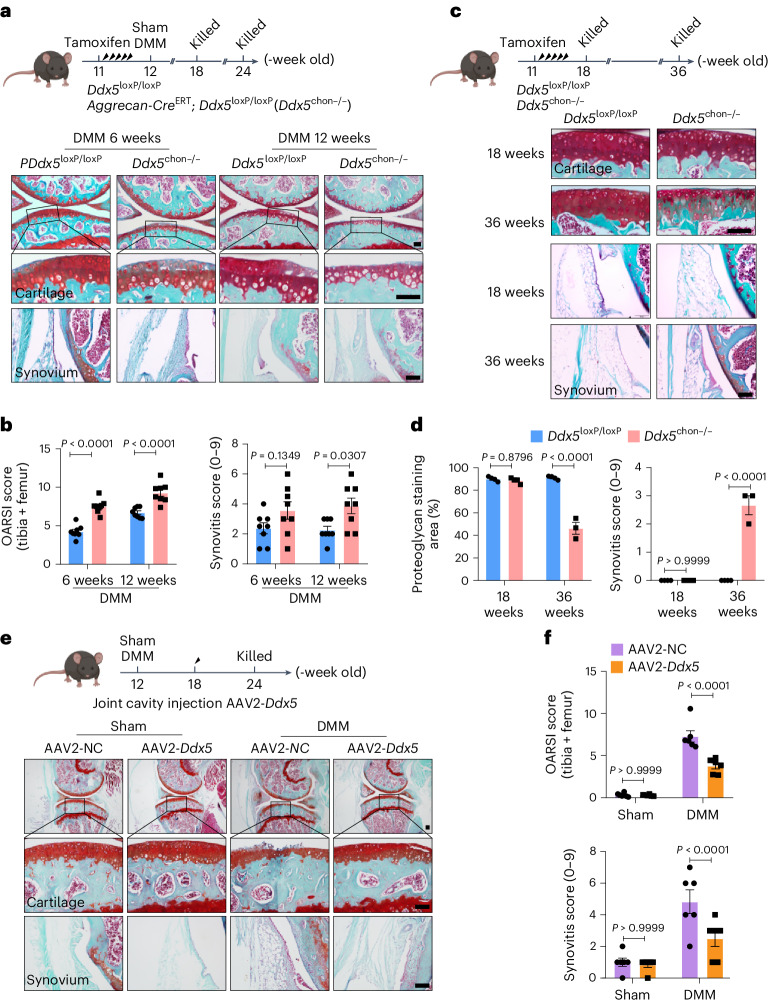
Regulation of DDX5 expression alters the progression of posttraumatic OA. **a**, Schematic of the experimental design. At 11 weeks of age, *Ddx5*^loxP/loxP^ and *Ddx5*^chon−/−^ male mice received five daily intraperitoneal injections of tamoxifen. One week after tamoxifen injection, mice underwent DMM surgery and were killed at 6 and 12 weeks postoperatively. SO&FG staining of the knee joint sections from *Ddx5*^loxP/loxP^ and *Ddx5*^chon−/−^ mice is shown. **b**, Corresponding OARSI and synovitis scores using histological sections (*n* = 8 mice per group). **c**, SO&FG staining of knee joint sections from *Ddx5*^loxP/loxP^ and *Ddx5*^chon−/−^ mice at 18 and 36 weeks. **d**, Quantification of the SO&FG staining area (red) and synovitis score using histological sections (*n* = 3 in 36-week-old *Ddx5*^loxP/loxP^ mice, *n* = 4 mice in the other groups). **e**, Representative joint sections from mice with DMM injected intra-articularly with AAV2-NC or AAV2-*Ddx5*, stained with SO&FG. **f**, OARSI and synovitis scores (*n* = 6 mice per group). **a**,**c**,**e**, Scale bars, 100 µm. All data are expressed as the mean ± s.e.m. **b**,**d**,**f**, A two-way analysis of variance (ANOVA) with Šidák’s correction was conducted. Source data

**Extended Data Fig. 8:**
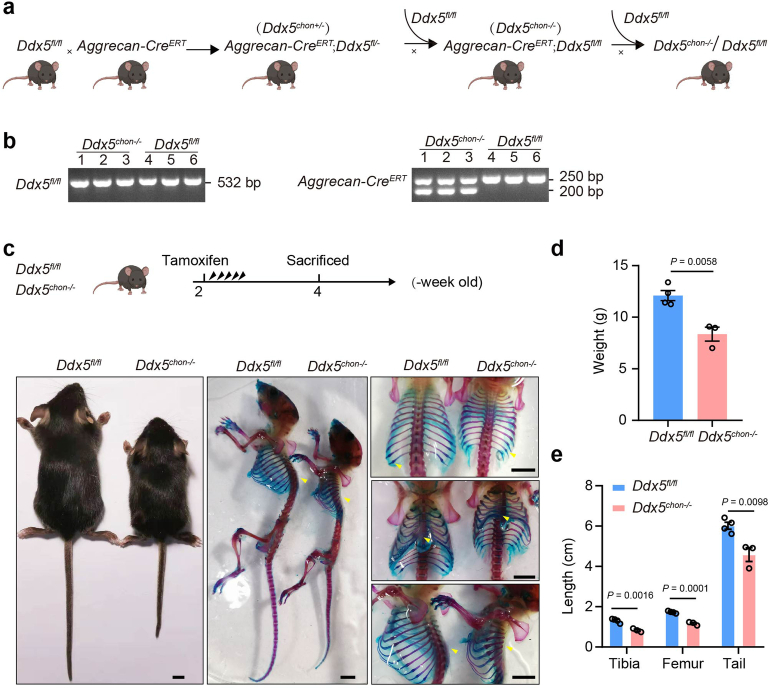
Genetic knockdown of Ddx5 delays early development in mice. (**a**)The deletion of *Ddx5* affected early development in mice, resulting in impaired skeletal development, including shortened limbs and tails, as well as abnormal ribs. Mating strategies were employed in mice. (**b**) Genotyping was detected using mice tail PCR. (**c**) Staining of mouse skeletons showed that *Ddx5* deletion could result in impaired skeletal development, including short limbs and tails, and abnormal ribs (indicated by yellow arrows). Scale bars, 0.5 cm. (**d**, **e**) In tissue sections were examined after 2 weeks after tamoxifen injection, and the weight (d) and length of femurs, tibias and tails (e) were counted. *Ddx5*^chon-/-^ mice, n = 4; *Ddx5*^fl/fl^ mice, n = 3. All data are presented as the mean ± SEM. Unpaired Student’s *t* test (d, e) was conducted. Source data

**Extended Data Fig. 9:**
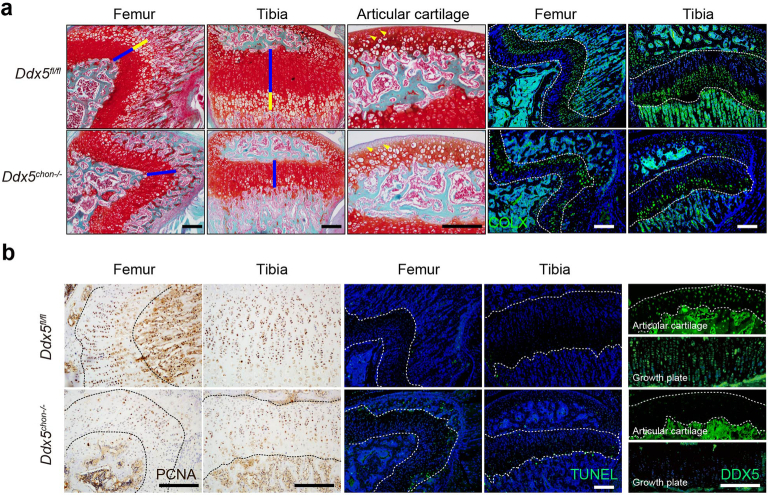
Genetic knockdown of Ddx5 promotes chondrocytes apoptosis and inhibits chondrocytes proliferation in the early stages of mouse development. (**a**) In tissue sections taken 2 weeks after tamoxifen injection, knee joint sections of *Ddx5*^fl/fl^ and *Ddx5*^chon-/-^ mice were detected by SO&FG staining. COLX expression was detected by IF. The SO&FG staining showed significant shortening of the growth plate in *Ddx5*^chon-/-^ mice, affecting both hypertrophic and non-hypertrophic regions, and causing a slight loss of articular cartilage. (**b**) PCNA expression was detected by IHC. Cell apoptosis was detected by TRUNEL kit (Scale bars, 100 μm). DDX5 expression was detected by IF (Scale bars, 200 μm). The blue and yellow lines in the panel represented the hypertrophic and non-hypertrophic cartilage thickness at the growth plate, respectively. The white or black dotted lines frame the cartilage or growth plate area. Source data

Next, we sought to determine whether the selective removal of DDX5 aggravates the progression of OA and its symptoms. To investigate whether DDX5 is necessary for chondrocyte function in OA pathogenesis, we used *Aggrecan-Cre*^ERT^ transgenic mice that express a tamoxifen-activated Cre recombinase exclusively in chondrocytes to selectively delete *Ddx5* (Extended Data Fig. 8a,b). Using tamoxifen, we induced delayed skeletal development in adult mice by knocking out *Ddx5* in the early developmental stage (Extended Data Fig. 8c–e and Extended Data Fig. 9). Then, we induced posttraumatic OA in *Ddx5*^loxP/loxP^ and *Aggrecan-Cre*^ERT^; *Ddx5*^loxP/loxP^ mice (*Ddx5*^chon−/−^) using DMM surgery. Six and 12 weeks after DMM surgery, these *Ddx5*^chon−/−^ mice displayed reduced SO&FG staining of cartilage and more severe synovitis than *Ddx5*^loxP/loxP^ mice (Fig. 2a,b). Furthermore, investigation of natural aging revealed that structural integrity of the articular cartilage was relatively well preserved in 18-week-old *Ddx5*^chon−/−^ mice. However, at 36 weeks, *Ddx5*^chon−/−^ mice exhibited mild OA-like changes, including patchy reduction in SO&FG staining of cartilage and mild synovial inflammation (Fig. 2c,d), indicating spontaneous cartilage degeneration.

**Extended Data Fig. 10:**
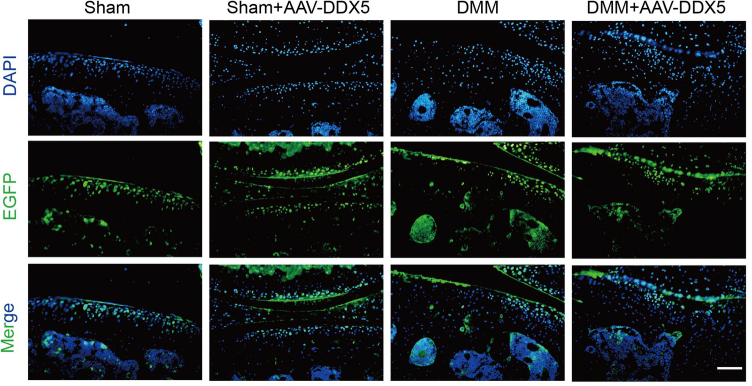
The transfection efficiency of AAV2 virus in cartilage of mice. The efficiency of AAV2 infection in articular chondrocytes was assessed by expressing enhanced green fluorescent protein (EGFP) using AAV2. After 6 weeks of DMM surgery in mice, the virus was injected into the joint cavity for 6 another 6 weeks. The expression of EGFP protein was detected using immunofluorescence. Scale bars, 200 μm. Source data

We next wondered whether overexpression of DDX5 could protect against OA progression caused by DMM in mice. We injected adeno-associated virus 2 (AAV2-*Ddx5*) expressing *Ddx5* or control AAV2 (AAV2-NC) intra-articularly. We evaluated the efficiency of AAV2 infection in articular chondrocytes by injecting AAV2 expressing enhanced green fluorescent protein (eGFP) intra-articularly. Six weeks after DMM surgery in mice, the virus was injected into the joint. Six weeks after injection, a strong eGFP signal was detected in articular chondrocytes (Extended Data Fig. 10). Intra-articular injection of AAV2 expressing *Ddx5* significantly ameliorated the progression of DMM-induced OA in mice (Fig. 2e,f), further corroborating that targeted expression of DDX5 in articular chondrocytes preserves integrity of articular cartilage and protects against instability-induced OA. These findings suggest that DDX5 is essential for maintaining the structural soundness of articular cartilage.

### DDX5 deficiency upregulates inflammation-related and fibrosis-related pathways

**Fig. 3:**
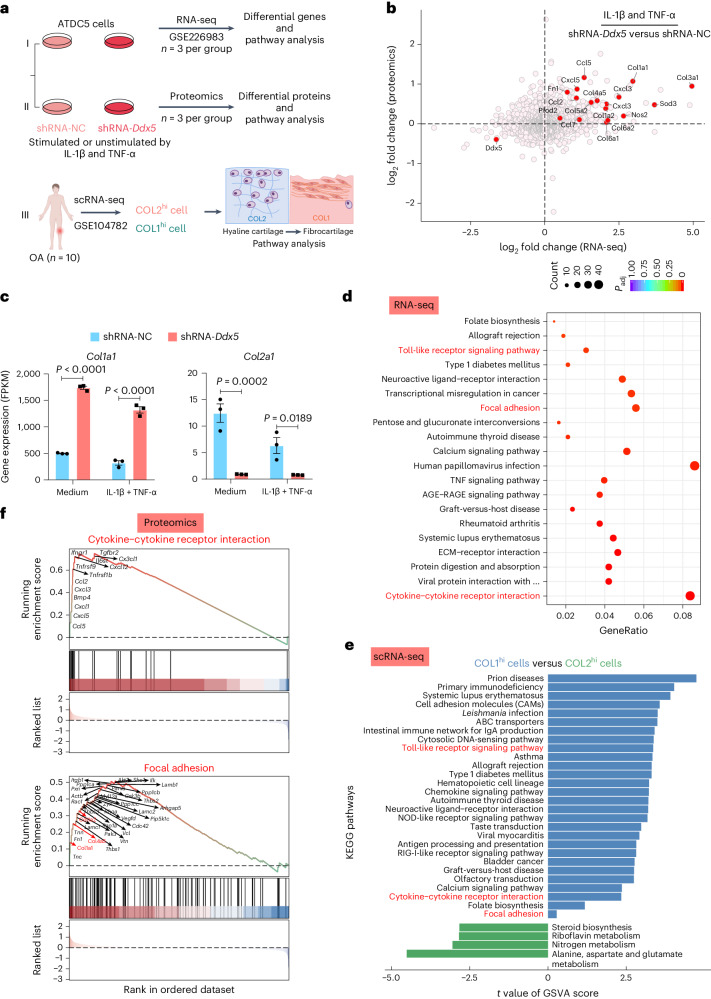
*Ddx5* knockdown results in the upregulation of inflammation-related and fibrosis-related pathways. **a**, Schematic of the experimental design. **b**, Bi-omics joint analysis was performed in ATDC5 cells stimulated with IL-1β combined with TNF-α. The dotted line represents the FDR (FDR < 0.05). **c**, Gene expression levels (shRNA-*Ddx5* versus shRNA-NC) from the RNA-seq analysis of ATDC5 cells (5 ng ml^−1^ IL-1β + 25 ng ml^−1^ TNF-α, 6 h). A two-way ANOVA with Šidák’s correction was conducted. The results are expressed as the mean ± s.e.m. FPKM, fragments per kilobase of transcript per million mapped reads. **d**, A KEGG term enrichment analysis was performed on the data (shRNA-*Ddx5* versus shRNA-NC, RNA-seq) in ATDC5 cells (5 ng ml^−1^ IL-1β + 25 ng ml^−1^ TNF-α, 6 h). **e**, KEGG enrichment pathway analysis of the scRNA-seq dataset (GSE104782) of patients with OA. GSVA, gene set variation analysis. **f**, GSEA analysis was performed on differentially expressed proteins determined using quantitative proteomics (shRNA-*Ddx5* versus shRNA-NC) in ATDC5 cells (5 ng ml^−1^ IL-1β + 25 ng ml^−1^ TNF-α, 24 h). **b**–**f**, *n* = 3 biologically independent experiments. Source data

To gain a fuller understanding of the consequences of *Ddx5* loss in OA on transcription and protein expression, we created *Ddx5* knockdown ATDC5 cells, a chondrogenic cell line, and performed RNA-seq and tandem mass tag (TMT) labeling-based quantitative proteomic analysis (Fig. 3a–f and Supplementary Fig. 1). RNA-seq analysis showed that *Ddx5* knockdown led to the upregulation of inflammation-related and fibrosis-related genes (Supplementary Figs. 2 and 3). Proteomic analysis identified 7,279 proteins, of which 7,184 were quantified. Proteins that demonstrated a 1.2-fold change and FDR < 0.05 were defined as demonstrating significantly different expression (Supplementary Fig. 4a). The volcano plot generated from the differential analysis of proteins demonstrated that deficiency of DDX5 significantly increased the expression of proteins related to cartilage fibrosis (Supplementary Fig. 4b). Furthermore, bi-omics joint analysis (RNA-seq and proteomic data) in short hairpin RNA (shRNA)-*Ddx5* versus shRNA-NC with cytokine stimulation, revealed that common targets were enriched. Among them, the fibrosis-related genes *Col1* (*Col1a1, Col1a2*) and *Col3a1* were concomitantly upregulated (Fig. 3b). Gene Ontology (GO) enrichment analysis similarly showed significant enrichment in terms related to collagen synthesis and assembly, based on increased expression of fibrosis-related genes (Supplementary Figs. 5 and 6). Additionally, RNA-seq data showed that the cartilage degradation-related genes *Mmp13*, *Nos2*, *Adamts4* and *Adamts5*, and *Sod3* were all upregulated, whereas *Col2a1* was downregulated (Fig. 3c and Supplementary Fig. 7).

The Kyoto Encyclopedia of Genes and Genomes (KEGG) analysis of the RNA-seq data showed that fibrosis-related pathways, including ECM receptor interaction, focal adhesion and protein digestion and absorption (Fig. 3d), were significantly enriched. In addition, chemokine–cytokine receptor interactions and Toll-like signaling pathway were significantly enriched in the RNA-seq data (Fig. 3d), indicating that *Ddx5* knockdown amplified inflammation-related responses. Similarly, the enrichment score of COL2^hi^ cells in the single-cell sequencing of OA clinical data (Fig. 3a–f) was significantly lower than that of COL1^hi^ cells for fibrosis-related and inflammation-related pathways (Fig. 3e). Also, the bi-omics joint analysis revealed significant enrichment of fibrosis-related and inflammation-related pathways (Supplementary Fig. 8). Additionally, gene set enrichment analysis (GSEA) of proteomic data indicated that these pathways corresponded to enriched proteins (Fig. 3f and Supplementary Fig. 9). Therefore, these results suggest that deficiency of DDX5 may trigger hyaline cartilage fibrosis and degeneration through the involvement of fibrosis-related and inflammation-related pathways.

### DDX5 is indispensable for inhibiting hyaline cartilage fibrosis and degradation

**Fig. 4:**
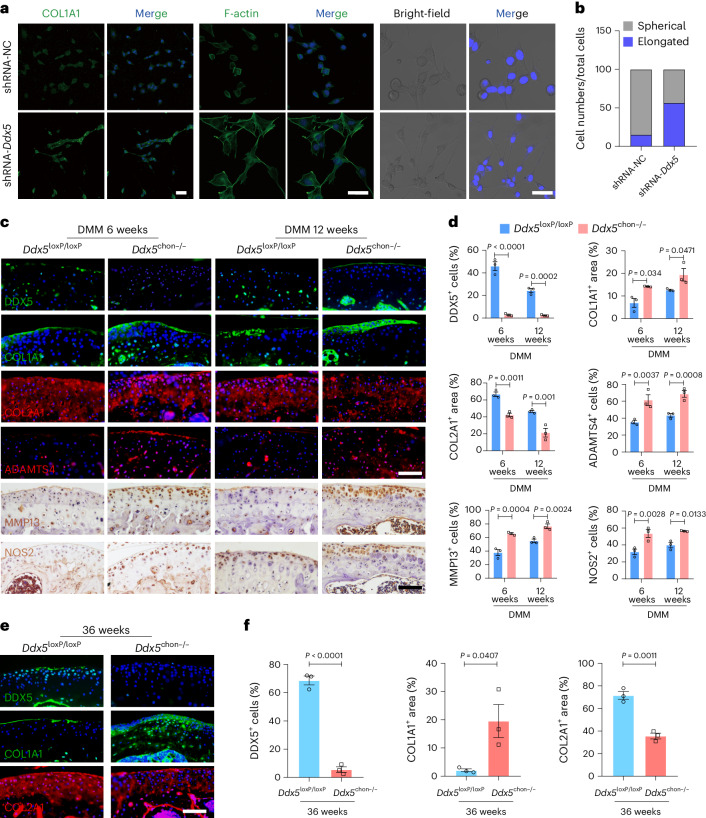
DDX5 reduces the fibrocartilage phenotype and inhibits cartilage degradation. **a**, Left, Representative IF images of COL1A1 expression in ATDC5 cells (shRNA-*Ddx5* versus shRNA-NC). Middle, F-actin was labeled using fluorescently conjugated phalloidin. ATDC5 cells were cultured in confocal dishes. Right, Nuclei were counterstained with Hoechst dye (blue); optical transmission (bright-field) images show cell morphology. **b**, Ratio of spherical and elongated cells to total cells. **c**, Representative images of DDX5, COL1A1, COL2A1, MMP13 and NOS2 expression in knee sections of *Ddx5*^loxP/loxP^ and *Ddx5*^chon−/−^ mice after DMM. **d**, Percentage of COL1A1^+^ and COL2A1^+^ areas, and number of cells stained for DDX5, MMP13 and NOS2 (*n* = 3 mice per group). **e**, Expression of DDX5, COL1A1 and COL2A1 in the knee sections of *Ddx5*^loxP/loxP^ and *Ddx5*^chon−/−^ mice at 36 weeks of age. **f**, Number of cells stained for COL1A1, COL2A1 and DDX5 in slices (*n* = 3 mice per group). **a**,**c**,**e**, Scale bars, 100 μm. All data are presented as the mean ± s.e.m. A two-way ANOVA with Šidák’s correction (**d**) and an unpaired Student’s *t*-test (**f**) were conducted. Source data

To further identify the molecular phenotype of DDX5 in protecting against cartilage degradation and fibrosis, we next determined the expression of cartilage degradation genes and the cartilage fibrosis phenotype. The experiment showed that knockdown of *Ddx5* increased production of COL1A1 and changed the morphology of ATDC5 cells, causing cells to shift from a spherical to an elongated pattern as observed in the F-actin and optical transmission (bright-field) images (Fig. 4a,b). We stimulated mice with combined IL-1β and TNF-α; the levels of NOS2 and ADAMTS5 were higher in the DDX5 knockdown group than in the controls (Supplementary Fig. 10). Using immunohistochemistry (IHC) and IF staining of sections from *Ddx5*^loxP/loxP^ and *Ddx5*^chon−/−^ mice at 6 and 12 weeks postoperatively, cartilage degeneration and fibrosis were confirmed after DMM surgery. IF and IHC showed that deletion of *DDX5* significantly increased production of COL1A1, MMP3, ADAMTS4 and NOS2 in *Ddx5*^chon−/−^ mice in the DMM model (Fig. 4c,d). We also observed a reduction in the expression of COL2A1 in *Ddx5*^chon−/−^ mice in the DMM model (Fig. 4c,d). We also evaluated the expression of COL1A1 and COL2A1 in 9-month-old *Ddx5*^chon−/−^ mice, which similarly showed upregulation of COL1A1 production and downregulation of COL2A1 production compared to littermate *Ddx5*^loxP/loxP^ mice (Fig. 4e,f). Taken together, these results suggest that *Ddx5*^chon−/−^ mice exhibited a more severe OA phenotype because of increased catabolism and cartilage fibrosis.

### DDX5 is required to splice key genes in OA

**Fig. 5:**
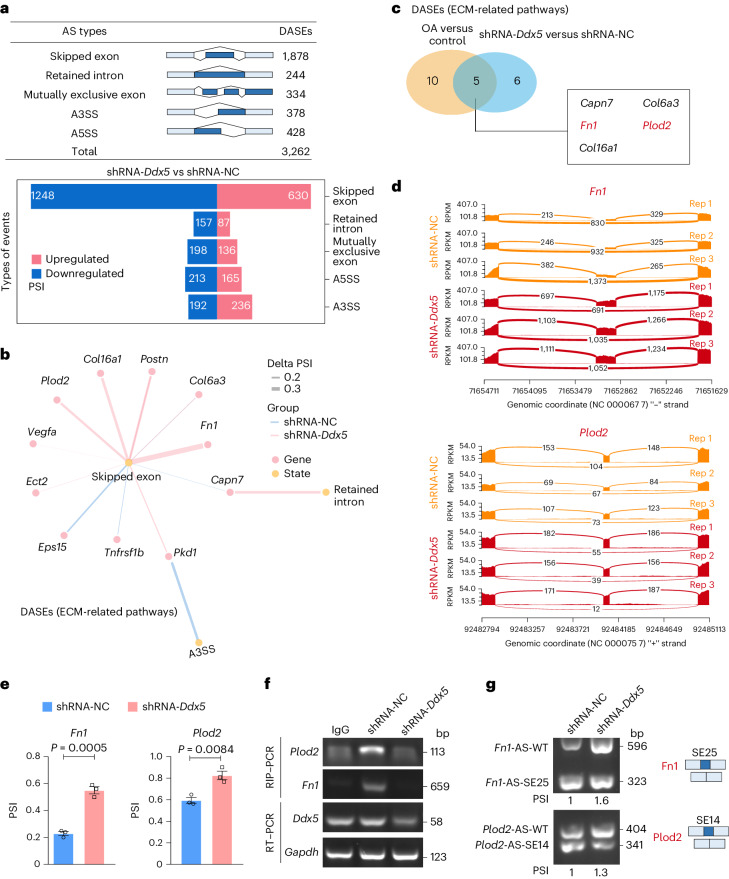
DDX5 is required to splice key genes in OA. **a**, Summary of the differential splicing analysis performed between shRNA-NC and shRNA-*Ddx5* ATDC5 cells (delta PSI > 10% and FDR < 0.05). The numbers of DASEs in each category on *Ddx5* deletion are indicated. **b**, DDX5-regulated DASEs related to ECM pathways in ATDC5 cells. **c**, Venn diagram indicating common ECM-related splicing genes between DDX5-regulated DASEs and human OA-associated DASEs. **d**, Sashimi plots showing an alternative exon skipping event in the *Fn1* and *Plod2* genes in shRNA-NC (yellow) and shRNA-*Ddx5* (red) ATDC5 cells. RPKM, reads per milobase per million mapped reads. **e**, The inclusion levels of the *Fn1* and *Plod2* genes were analyzed in ATDC5 cells using the rMATS software (shRNA-NC and shRNA-*Ddx5*) (*n* = 3 independent experiments). Data are presented as the mean ± s.e.m. An unpaired Student’s *t*-test was conducted. **f**, Enrichment of *Fn1* and *Plod2* by DDX5 was detected using a RIP–PCR assay. **g**, Splicing of alternative exons in ATDC5 cells (shRNA-NC and shRNA-*Ddx5*) was analyzed using PCR with reverse transcription (RT–PCR). Quantification is shown as the fold change of PSI relative to the control sample. **a**–**g**, ATDC5 cells were treated with combined IL-1β and TNF-α for 6 h. Source data

To elucidate the molecular mechanism of DDX5 in cartilage fibrosis and degeneration, we analyzed the AS events regulated by DDX5 in ATDC5 cells. Cells were stimulated with IL-1β and TNF-α; we used the rMATS software for the analysis. We identified a total of 3,262 DDX5-regulated AS events (Fig. 5a). One hundred and sixteen highly significant DASEs with delta PSI > 20% and FDR < 1 × 10^−8^ (Supplementary Fig. 11) were identified. DASEs related to ECM pathways are indicated (Fig. 5b). We then combined the analysis of DASEs related to ECM pathways in both the RNA-seq database of *Ddx5* knockdown and the clinical OA samples. Using a Venn diagram, we found that five candidate genes were collectively enriched (Fig. 5c). Interestingly, among these the mRNAs associated with DDX5, FN1 and PLOD2 are required to maintain collagen deposition and hydroxylation^20,21^. The results of the coding sequence analysis showed that pre-mRNA *Fn1* had more exon 25-containing variants, while pre-mRNA *Plod2* had more exon 14-containing variants in *Ddx5* knockdown ATDC5 cells (Fig. 5d,e). Additionally, rMATS analysis of the OA cartilage samples revealed that pre-mRNA *FN1* and *PLOD2* also had skipped exon-type splicing (Supplementary Fig. 12).

To determine whether the effect on AS was mediated by the direct binding of DDX5, we confirmed that *Fn1* and *Plod2* were bound to DDX5 using RNA immunoprecipitation (RIP)–PCR assays (Fig. 5f). Moreover, the isoforms of *Fn1*-AS-WT and *Plod2*-AS-WT were increased in cytokine-stimulated *Ddx5* knockdown ATDC5 cells compared to controls (Fig. 5g and Supplementary Fig. 13). These data collectively demonstrate that depletion of DDX5 represses exon skipping of pre-mRNA *Fn1* and *Plod2* in OA.

### Knockdown of *Fn1*-AS-WT and *Plod2*-AS-WT inhibits the transcription of fibrosis-related and ECM degradation genes and reverses the phenotype that aggravates OA

**Fig. 6:**
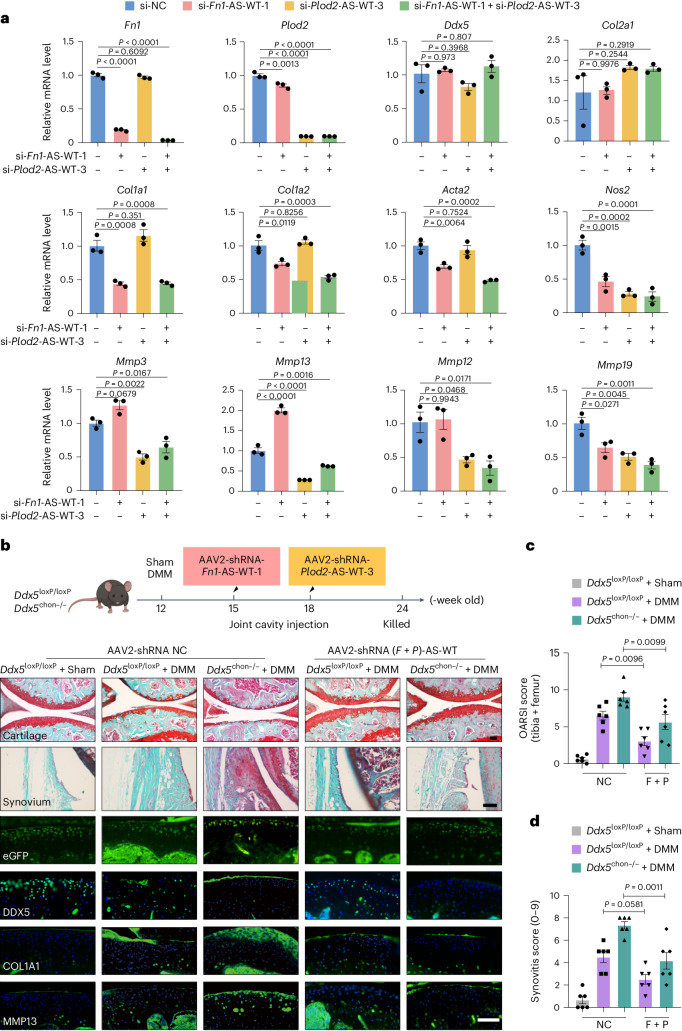
Knockdown of *Fn1*-AS-WT and *Plod2*-AS-WT inhibits transcription of fibrosis-related and ECM degradation genes and reverses the phenotype that aggravates OA, caused by DXX5 deficiency. **a**, qPCR analysis of the indicated genes in ATDC5 cells transfected with two small interfering RNAs (siRNAs) alone or in combination for 24 h (*n* = 3 per group repetition). **b**, Upper, Schematic illustrating the experimental design. Bottom left, Representative SO&FG and IF staining of eGFP, DDX5, COL1A1 and MMP13 in sham and DMM joint sections from mice injected intra-articularly with AAV2-NC or AAV2-shRNA-(*Fn1* + *Plod2*)-AS-WT. Scale bar, 100 µm. **c**,**d**, Bottom right, OARSI (**c**) and synovitis (**d**) scores (*n* = 6 mice per group). All data are presented as the mean ± s.e.m. In **a**,**c**,**d**, a one-way ANOVA with Tukey’s multiple comparisons test was conducted. Source data

We next examined the functions of different isoforms of *FN1*-AS and *PLOD2*-AS in OA. First, we knocked down *Fn1*-AS-WT and *Plod2*-AS-WT in primary mouse chondrocytes and ATDC5 cells. Knockdown of *Fn1*-AS-WT significantly decreased fibrosis by inhibiting *Col1a1* and *Col1a2* and *Acta2* expression, while knockdown of *Plod2*-AS-WT markedly reduced ECM degradation by inhibiting *Nos2*, and *Mmps3* *Mmps12*, *Mmps13*, *Mmps19*, expression (Fig. 6a and Supplementary Fig. 14). Combined knockdown of *Fn1*-AS-WT and *Plod2*-AS-WT simultaneously reduced the expression of fibrosis-related and ECM degradation genes (Fig. 6a). Next, we explored whether AAV2-shRNA-*Fn1*-AS-WT or AAV2-shRNA-*Plod2*-AS-WT via intra-articular injection could protect against cartilage defects caused by microfracture (MF). The results showed that treatment of AAV2-shRNA-*(Fn1* *+* *Plod2)*-AS-WT significantly restored defective cartilage and partially reversed the DDX5 deficiency-induced, OA-aggravating phenotype in mice (Supplementary Fig. 15). Moreover, to examine whether knockdown of the *Fn1*-AS-WT and *Plod2*-AS-WT variants can reverse the OA-aggravating phenotype caused by DDX5 deficiency, we used a DMM-induced mouse model of OA. The efficiency of AAV2 infection in articular chondrocytes was assessed by intra-articular injection of AAV2-expressing eGFP (Fig. 6b). Treatment with AAV2-shRNA-(*Fn1* *+* *Plod2*)-AS-WT significantly inhibited cartilage injury in *Ddx5*^chon−/−^ mice (Fig. 6b). ECM degradation and fibrosis in mice administered combined treatment was significantly alleviated compared to controls, as evidenced by the production of MMP13 and COL1A1 (Fig. 6b). Importantly, DMM caused dramatic cartilage loss and synovitis, which was largely ameliorated in the AAV2-shRNA-(*Fn1* *+* *Plod2*)-AS-WT-treated group compared to controls (Fig. 6b–d). Together, these data suggest that depletion of DDX5 produces more isoforms of *Fn1*-AS-WT and *Plod2*-AS-WT to promote cartilage fibrosis and degradation.

### G4 structure in the *Col2* promoter region

**Fig. 7:**
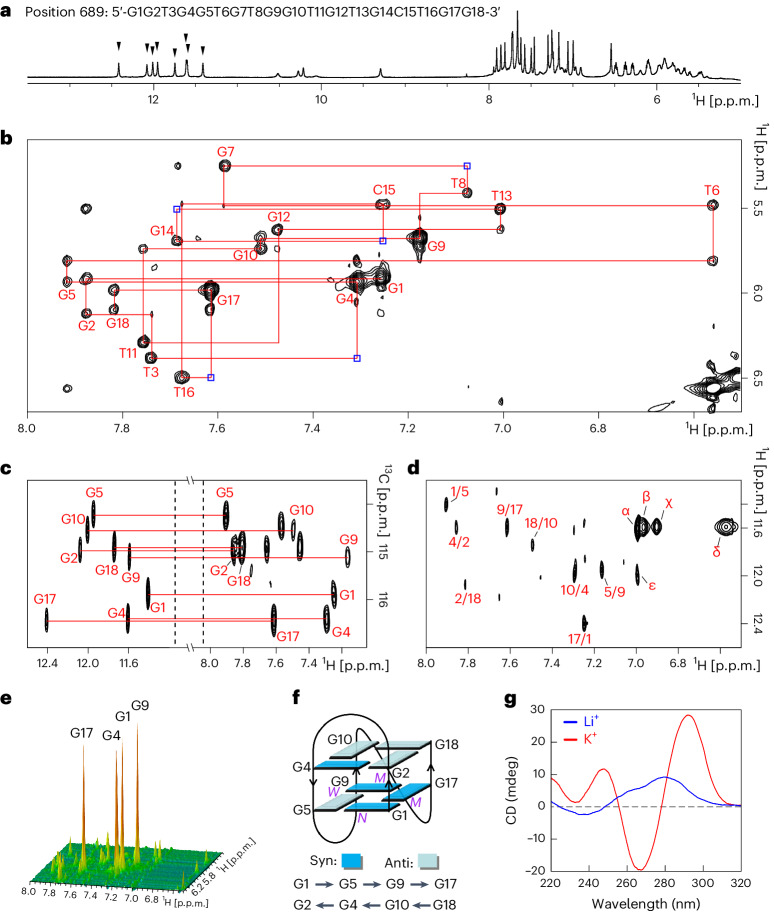
Determination of the position 689 folding topology of the *Col2* promoter. **a**, Residue number and one-dimensional ^1^H NMR spectrum of position 689. The imino proton peaks of the G4 folded according to position 689 are denoted by the downward arrowheads. **b**, H8/6-H1′ sequential connectivity in the NOESY spectrum (250 ms mixing time, 100% D_2_O) at 298 K. Intra-residue NOEs are labeled with residue numbers. Weak or missing sequential connections are labeled with blue rectangles. **c**, The HMBC spectrum shows the through-bond correlations between imino H1 and base H8 protons from the same guanine residue via ^13^C5 at natural abundance. **d**, The NOESY spectrum (250 ms mixing time, 10% D_2_O and 90% H_2_O) reveals inter-residue H1–H8 cross-peaks for the identification of the arrangement of the G-tetrads. The guanine H1–H8 cross-peaks are labeled with the residue numbers of the H1 and H8 protons in the first and second positions, respectively. The remaining strong cross-peaks are identified as: α, G4H1-T13H6; β, G9H1-C15H22; χ, G9H1-C15H21; δ, G9H1-G9H22; and ε, G10H1-T13H6. **e**, Stacked plot of the two-dimensional NOESY spectrum (50 ms mixing time, 100% D_2_O) of position 689. **f**, Schematic folding topologies of position 689. The hydrogen bond directionality from donor (arrow tail) to acceptor (arrow head) within the same G-tetrad is shown in the same row by the arrows. W, M and N represent wide, medium and narrow groove widths, respectively. **g**, CD spectra of G4 folded according to position 689 in 100 mM Li^+^ (blue) or K^+^ (red). Source data

As the knockdown of both *Fn1*-AS-WT and *Plod2*-AS-WT isoforms did not affect COL2 expression, there might be other mechanism by which DDX5 regulates COL2 expression. COL2 is the most abundant matrix molecule of cartilage and is essential for maintaining the structural integrity of cartilage. Thus, the regulation of COL2 expression is crucial for maintaining collagen stability. Considering that DDX5 acts as a helicase, and participates in RNA splicing, it can resolve both DNA-formed and RNA-formed G4 to regulate gene expression^22^. DDX5 unfolded a G4 DNA motif in the proximal G/C-rich promoter region of the *c-Myc* gene, which acts as a transcriptional silencer^23^. QGRS Mapper^24^ predicted the presence of 14 putative quadruplex sequences in the proximal G/C-rich promoter region within 2,000 bp upstream of the *Col2* promoter. Nuclear magnetic resonance (NMR) experiments showed that only position 689, located in the promoter region 689 upstream of *Col2*, could fold into the G4 structure in K^+^ solution (Supplementary Table 1). In the one-dimensional ^1^H NMR spectrum of position 689 with a 100-mM K^+^ solution at 310 K and pH 6.8, eight sharp guanine imino proton signals were well resolved at approximately 11.5–12.5 p.p.m. These signals were in the fingerprinting region for G4 formation (Fig. 7a and Supplementary Fig. 16).

As the NMR spectra showed good resolution of the signal peaks of imino and aromatic protons of G4 folded by position 689 (Supplementary Figs. 16 and 17a), we determined the G4 topology. Assignments of nonexchangeable H8/H6 base proton and sugar H1′ proton were accomplished by tracing the sequential nuclear Overhauser effect (NOE) connectivities in the nuclear Overhauser effect spectroscopy (NOESY) spectrum, which had a mixing time of 250 ms recorded in 100% D_2_O (Fig. 7b)^25^. The H6-CH3 of thymine and H5-H6 of cystine were assigned using the total correlation spectroscopy and NOESY spectra (Supplementary Fig. 17b). Assignments of guanine imino proton were achieved using the heteronuclear multiple bond correlation (HMBC) experiment (Fig. 7c). This experiment is based on the correlation between guanine H8 base and imino H1 protons through ^13^C5 at natural abundance, using long-range J-couplings^26^. Furthermore, the hydrogen bond alignments and directionality within each G-tetrad were determined based on the establishment of H1–H8 connectivities in the NOESY spectrum, with a mixing time of 250 ms in 10% D_2_O and 90% H_2_O (Fig. 7d). This yielded a total of two G-tetrads, including G1 → G5 → G9 → G17 and G2 ← G4 ← G10 ← G18. In the stacked NOESY spectrum with a short mixing time of 50 ms, four strong H8-H1′ cross-peaks were observed for residues G1, G4, G9 and G17, indicating that they adopted a syn glycosidic conformation (Fig. 7e). Accordingly, the folding topology of position 689 was established as a (3 + 1) intramolecular G4, which consists of two G-tetrad layers (Fig. 7f). This conclusion is also supported by the circular dichroism (CD) spectrum (Fig. 7g).

### DDX5 resolves G4 in the *Col2* promoter and unfolding of G4 in the *Col2* promoter enhances COL2 expression

**Fig. 8:**
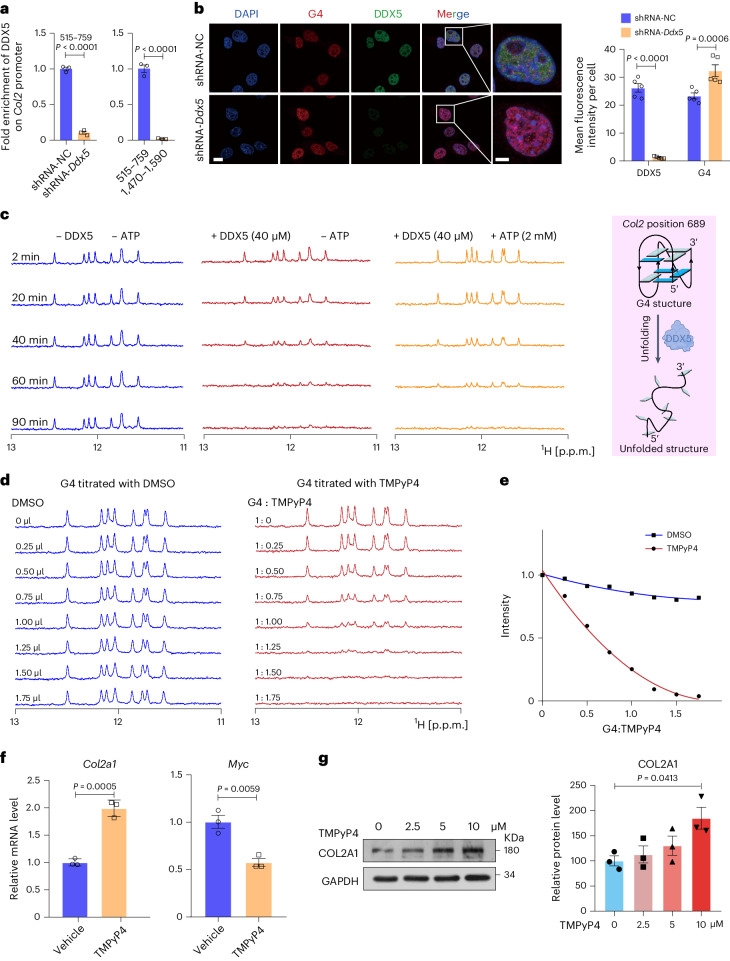
DDX5 resolves G4 in the *Col2* promoter and TMPyP4 unfolds G4 in the *Col2* promoter and enhances COL2 expression. **a**, ChIP was performed with an anti-DDX5 antibody in shRNA-NC and shRNA-*Ddx5* ATDC5 cells. qPCR analysis of the promoter region (515–759 bp) of the *Col2* gene (1,470–1,590 bp region as the negative control, *n* = 3 per group repetition) is shown. **b**, Representative IF staining of DDX5 and G4 in shRNA-NC and shRNA-*Ddx5* ATDC5 cells. The mean fluorescence intensity (intensity per area) of each cell was calculated using Image J (*n* = 5 cells). **c**, NMR tracing of DDX5 unfolding of the *Col2* promoter G4 at position 689. **d**, Expanded imino proton regions of one-dimensional ^1^H NMR spectra were recorded for prefolded G4 position 689 titrated with 0–1.75-equivalent TMPyP4. **e**, Variation of peak intensity of G4 position 689 with 0–1.75-equivalent TMPyP4 titrations. **f**, qPCR analysis of *Col2a1* and *Myc* in primary mouse chondrocytes treated with TMPyP4 (5 μM, 24 h, *n* = 3 repetition). **g**, Immunoblot analysis of COL2 in primary mouse chondrocytes (at the indicated doses of TMPyP4, 24 h). Image J was used for the density measurements (*n* = 3 biologically independent experiments). All data are presented as the mean ± s.e.m. **b**, Scale bar, 5 μm. A two-way ANOVA with Šidák’s correction (**b**), a Student’s *t*-test (**a**,**f**) and a one-way ANOVA with Dunnett’s multiple comparisons test (**g**) were conducted. Source data

As DNA G4s form in the *Col2* promoter, we hypothesized that DDX5 regulates *Col2* expression through a G4-dependent mechanism. To test this hypothesis, we conducted chromatin immunoprecipitation (ChIP) experiments in mouse chondrocytes to determine whether DDX5 directly interacts with the *Col2* G4-forming promoter region in vivo. The ChIP–quantitative PCR (qPCR) results demonstrated that DDX5 directly interacted with the *Col2* promoter, as evidenced by the enrichment of the *Col2* G4-forming promoter region (Fig. 8a). Then, we visualized cellular G4s through immunostaining by using the selective G4 antibody BG4 in both shRNA-NC and shRNA-*Ddx5* ATDC5 cells to measure the accumulation of DDX5 in the nucleus. shRNA-NC ATDC5 cells displayed clear accumulation of DDX5 in the nucleus; this accumulation was associated with a significant reduction in BG4 staining (Fig. 8b). Indeed, knocking down *Ddx5* remarkably increased the fraction of BG4^+^ cells in the nucleus (Fig. 8b). Then, we sought to validate the unfolding of *Col2* G4. To investigate the unfolding activity of DDX5, we conducted time course analyses of the *Col2* promoter G4 unfolding by DDX5 using an NMR assay. We expressed and purified the DDX5 protein (Supplementary Fig. 18a) according to the method reported by Xing et al.^27^. We then examined the effect of K^+^ and Na^+^ because they could stabilize the G4 structures. The activity of DDX5 in binding and unfolding double-stranded RNA was ATP-dependent^28^. Surprisingly, our results showed that the unfolding activities of DDX5 on the *Col2* G4 structure occurred in the absence of ATP (Fig. 8c). Furthermore, the presence of ATP might slightly impede the unfolding speed of the G4 structure by DDX5, possibly because some DDX5 proteins were used for ATP hydrolysis (Fig. 8c and Supplementary Fig. 18b,c).

We next sought to confirm whether *Col2* G4 affected the expression level of COL2. TMPyP4 is a compound that interacts with G4 and stabilizes DNA G4 structures^29,30^. TMPyP4 can have both stabilizing and destabilizing effects on RNA G4 structures^31–33^. Interestingly, using NMR spectroscopy, we revealed that TMPyP4 strongly bound to *Col2* genomic DNA (gDNA) and unfolded it (Fig. 8d,e). After treatment with TMPyP4, the mRNA level of *Col2a1* was increased (Fig. 8f). The mRNA level of *Myc*, which was used as a positive control, decreased, as shown in Fig. 8f, as *Myc* G4 DNA is stabilized by TMPyP4 (ref. ^13^). Immunoblot analysis also showed that COL2 expression increased in a concentration-dependent manner on treatment with TMPyP4 in primary mouse chondrocytes (Fig. 8g). Taken together, these data indicate that DDX5 actively unfolds *Col2* and this process is independent of ATP hydrolysis. Destabilization of *Col2* G4 by the small-molecule TMPyP4 increases the expression level of COL2.

## Discussion

Currently, there is no effective treatment strategy for fibrocartilage in the clinical treatment of OA. Therefore, it is crucial to clarify the mechanism of hyaline cartilage fibrosis. Our study demonstrates that DDX5 deficiency produces isoforms of *Fn1-*AS-WT and *Plod2*-AS-WT, which promote cartilage fibrosis and degradation. Moreover, this study provides evidence for a mechanism of DDX5 involvement in COL2 expression by directly unfolding G4 in the promoter region of *Col2*; to our knowledge, this has not been reported before. Collectively, these findings suggest that developing strategies to upregulate DDX5 can greatly benefit the treatment of OA.

Katsoula et al.^34^ conducted RNA-seq on paired samples of low-grade and high-grade OA knee cartilage, and identified a molecular map of long noncoding RNA expression, isoform switching and AS in OA^34^. The difference between our study and the study by Katsoula et al. is that we analyzed genome-wide differential splicing between healthy and OA cartilage. Furthermore, the study by Li et al.^12^ and our study indicated that there are many DASEs related to ECM-related pathways between healthy controls and individuals with OA. Li et al. identified TIA1 as a key regulator of these DASEs. We demonstrated that DDX5 was an important splicing regulator involved in *Col2* G4 unfolding in OA progression. In addition, DDX5 was involved in ECM-related pathways by regulating the expression of genes related to cartilage fibrosis, degradation and synthesis. Interestingly, under the same parameters (delta PSI > 20% and FDR < 0.0001) as those used by Li et al., some of the same DASEs, such as SRSF7, PRG4 and ABI3BP, were found in both groups, while other DASEs were identified in the present study, including TACC1, ENOSF1 and RNF146 (Extended Data Fig. 1).

The RNA-seq results revealed that some genes regulating the degradation of cartilage were also upregulated after *Ddx5* knockdown. However, related proteins were not enriched by proteomic analysis, possibly because of the limitations of proteomic technology in quantifying proteins. Subsequently, identification by higher expression of degradation-related proteins in the cartilage of *Ddx5*^chon−/−^ mice and *Ddx5* knockdown in ATDC5 cells clarified that *Ddx5* deficiency upregulated the expression of cartilage-degrading enzymes. Deletion of *Ddx5* promoted chondrocyte apoptosis and inhibited chondrocyte proliferation during the early stages of mouse development (Extended Data Fig. 9). Upregulation of the apoptosis signaling pathway and downregulation of the Ras signaling pathway also supported this phenotype (Supplementary Figs. 2 and 19). In addition, we found that TmPyP4 significantly decreased the protein level of DDX5 rather than its mRNA level (Supplementary Fig. 20a–c). It is possible for TMPyP4 to reduce the expression of DDX5 by stabilizing *Ddx5* G4. As TMPyP4 is not a highly specific small-molecule regulator of G4, it can potentially affect the structures of G4 in many genes. A decrease in DDX5 downregulated the expression of COL2, as shown in Figs. 4 and 5. However, the final result showed upregulation of COL2 after TMPyP4 treatment (Fig. 8g and Supplementary Fig. 20c). Although seeming contradictory, we suggest that the impact of downregulated DDX5 on *Col2* G4 was completely blocked by TMPyP4, which continuously and directly unfolded *Col2* G4 (Supplementary Fig. 20d).

DDX5 is involved in the AS of many important genes related to tumors; it promotes the occurrence and progression of cancer and is highly expressed in many cancer types^35^. Our study shows that DDX5 is abnormally downregulated in OA. One of the reported main functions of DDX5 is to participate in selective RNA splicing^36–38^. The results of our GO analysis clearly show that terms such as RNA metabolic regulation and RNA metabolic process regulation were enriched in *Ddx5-*deficient ATDC5 cells (Supplementary Fig. 5b,d). Hydroxylysine residue is a critical participant in collagen biosynthesis. Lysyl hydroxylation to hydroxylysine is catalyzed by PLOD2 (ref. ^39^). The collagen substrate of PLOD2 is COL1 (ref. ^40^). Thus far, PLOD2-related studies have been performed on fibrotic diseases and cancers such as Bruck syndrome, Ehlers–Danlos syndrome and carcinomas^41–44^. *Plod2* has two transcript variants. *Plod2*-AS-SE14 lacks an internal in-frame coding exon compared with full-length *Plod2* (*Plod2*-AS-WT), resulting in a shorter isoform (also known as LH2a)^40^. Dardenne et al.^36^ reported the effect of DDX5 knockdown on *Plod2* splicing in epithelial cells, which is similar to our results in chondrocytes. We demonstrated that knockdown of full-length *Plod2* has no impact on the expression of genes related to cartilage fibrosis. However, further investigation is required to determine whether the *Plod2* gene affects COL1 cross-linking in chondrocytes. FN1 is a high-molecular-weight multifunctional glycoprotein; its pre-mRNA has three AS sites: extra domain A (EDA); extra domain B (EDB); and a type III homology-connecting segment, which generate 20 different isoforms^10^. However, *Fn1*-AS-SE25 lacks the extra domain A (FN1-EDA)^45^. Accumulated evidence demonstrated that the FN1-EDA domain has an emerging role in some fibrotic diseases of many organs, including the dermis, liver, lung and bone marrow^46–48^. The increased cartilage fibrosis phenotype was caused by an elevation in the EDA^+^:EDA^−^ ratio. This explains how the upregulation of full-length *Fn1* and the concomitant decrease in the *Fn1*-AS-SE25 ratio results in increased COL1 production.

Furthermore, previous studies showed that DDXs, inducing DDX1 and DDX25, can bind to G4 structures present in intronic switch RNA^20,21^. A recent study found that DDX5 could unfold RNA G4 (ref. ^22^). DDX5 regulates RNA processing and metabolism by unfolding short double-stranded RNAs in an ATP-dependent manner. In this study, we showed that DDX5 could bind directly to the G4 promoter region of *Col2* DNA and increase COL2 production, in addition to its function of pre-mRNA splicing in *Fn1* and *Plod2*. Interestingly, we found that this process is ATP-independent and distinct from its function as an RNA helicase. Our research has some limitations. For instance, we did not investigate the reasons behind the decreased expression of DDX5. DDX5 functions as part of the spliceosome and is unable to recognize sequence specificity in its RNA substrates^49^. This suggests that other cofactors may be recognizing the alternative splice site in *Fn1* and *Plod2* pre-mRNAs. Immunoprecipitation and mass spectrum analysis identified multiple factors that interact with DDX5, including serine-rich and arginine-rich splicing factors^50^. Further investigation is needed to understand how DDX5 interacts with splicing factors to facilitate *Fn1* and *Plod2* pre-mRNAs.

Our results support the important role of DDX5 in regulating splicing and unfolding of G4 in chondrocytes (Supplementary Fig. 21). These defects in splicing and abnormalities in G4 unfolding may lead to severely impaired gene expression or inappropriate translation of protein isoforms. This may explain the gradual loss of the hyaline cartilage phenotype observed on *Ddx5* ablation. In conclusion, DDX5 can inhibit hyaline cartilage fibrosis and degradation in OA by pre-mRNA splicing and G4 unfolding. Targeted upregulation and expression of DDX5 in chondrocytes could potentially represent a therapeutic approach to tackling cartilage fibrosis and degeneration in OA.

## Methods

### Cell isolation and culture

The ATDC5 cell line was donated by J. Luo (Tongji University) and cultured with DMEM (Gibco) with 5% FCS and 1% penicillin-streptomycin (Thermo Fisher Scientific). Primary mouse chondrocytes were obtained from the knee joint cartilage of mice within 3 days of birth. Knee joints were dissected from the tibia plateau and femoral condyle articular cartilage of mouse pups and digested in 0.2% type II collagenase (Sigma-Aldrich) for 4 h at 37 °C; chondrocytes were collected using centrifugation. Primary chondrocytes were grown in a 1:1 mixture of DMEM/F-12 medium (catalog no. 10-092-CVR, Corning) supplemented with 5% FCS and 1% penicillin-streptomycin. All cells were maintained at 37 °C under 5% CO_2_.

### Animal model

The *Ddx5*^loxP/loxP^ mice were generated by the Shanghai Model Organisms Center. The *Aggrecan-Cre*^ERT^ mice were provided by G. Zhang (Hong Kong Baptist University). The chondrocyte-specific *Ddx5* knockout mice (*Aggrecan-Cre*^ERT^*; Ddx5*^loxP/loxP^) were generated by crossing *Ddx5*^loxP/loxP^ mice with *Aggrecan-Cre*^ERT^ mice. Male mice (*Ddx5*^loxP/loxP^ and *Aggrecan-Cre*^ERT^*; Ddx5*^loxP/loxP^ mice) were injected with 45 mg kg^−1^ tamoxifen intraperitoneally for five consecutive days to induce specific knockout of *Ddx5* in chondrocytes at 11 weeks of age. Tamoxifen was dissolved in corn oil at a concentration of 10 mg ml^−1^. Mice then underwent DMM or MF surgery. For the DMM model, the medial meniscus on the right knee was destabilized by surgically transecting the medial meniscus ligament^51,52^. Sham surgery was performed on independent mice. The cartilage injury score was based on the OARSI score^53^. A set of eight images of the medial tibial plateau and femoral condyle of each mouse were scored twice by experienced scorers. The sum of the average femoral and tibial scores from eight images was used as the final OARSI score. Synovitis scores were evaluated as reported previously^54^. In MF surgery, the cartilage defect mouse model was induced according to a previously established protocol. To assess the regeneration ability of articular cartilage after MF surgery, 12-week-old male littermates of *Ddx5*^loxP/loxP^ and *Aggrecan*-*Cre*^ERT^; *Ddx5*^loxP/loxP^ mice were used, according to the established protocol for the MF surgery-induced cartilage defect mouse model^55^. Histological assessment of cartilage repair in MF surgery was performed according to the International Cartilage Repair Society^56^. After 8 weeks, the repaired articular cartilage was analyzed histologically and scored for correlation. All animal experiments were conducted in accordance with the National Institutes of Health Guide for the Care and Use of Laboratory Animals and were approved by the Experimental Animal Care and Use Committee of Nanjing University (no. IACUC-2210005).

### Clinical samples

Articular cartilage was collected from patients with OA who underwent total knee arthroplasty. According to the research methods outlined by Snelling et al.^57^ and Wu et al.^58^, undamaged and damaged cartilage was separated from each individual. The articular cartilage samples from patients with OA were obtained from Nanjing Drum Tower Hospital. The clinical and demographic characteristics of the study population datasets are listed in Supplementary Table 2. This study was approved by the Ethical Committee of Nanjing Drum Tower Hospital, which is affiliated to Nanjing University Medical School (2020-156-01). We obtained written informed consent from patients with OA.

### Masson staining

Masson staining was performed with a Masson Stain Kit (Trichrome TISSUE-TROL Control Slides, catalog no. G1006, Servicebio). Experimental procedures strictly followed the manufacturer’s instructions. Images were captured with an Olympus BX51 Fluorescence Microscope (Olympus Life Science).

### SO&FG staining

SO&FG staining was performed to examine proteoglycans in the cartilage. Before SO staining, tissue slices were dewaxed. Then, an SO solution (0.1%, w/v) was added and incubated for 5 min. Fast green (0.05%, w/v) was added and incubated for another 5 min. After treating the tissue with glacial acetic acid for 10 s, it was washed with 95% alcohol for 2 min, followed by 100% alcohol for another 2 min, and finally with xylene for 5 min. Tissue slices were sealed with neutral gum and images were obtained with an Olympus BX51 Fluorescence Microscope.

### Real-time qPCR

Total RNA was extracted from articular cartilage tissue using the TransZol Up Plus RNA Kit (catalog no. ER501-01, Transgen Biotech); 1 μg RNA was used as input material for the RNA sample preparations. Total RNA was extracted from cells using TRIzol (Takara Bio). Reverse transcription to complementary DNA was prepared using SuperScript II Reverse Transcriptase (Invitrogen) and oligo (deoxythymidine) primers. Real-time qPCR was performed using the SYBR Premix Ex Taq II reagent with a CFX 100 (Bio-Rad Laboratories). *Gapdh* mRNA levels were used as an internal control for the target mRNAs. Normalization and fold changes were calculated using the $\Delta\Delta^{\mathrm{C}_{\mathrm T}}$ method. The primer sets are listed in Supplementary Table 3.

### Immunoblot

The immunoblot was performed based on our previously established protocol^59^. Cells were lysed with radioimmunoprecipitation assay lysis buffer supplemented with protease and phosphatase inhibitors (Roche) and then quantified using a bicinchoninic acid assay. Then, samples were separated using SDS–polyacrylamide gel electrophoresis and transferred onto polyvinylidene fluoride membrane. The membranes were then incubated with primary antibodies overnight at 4 °C, and then incubated with a horseradish peroxidase-coupled secondary antibody. Antibodies are listed in Supplementary Table 4. Detection was performed using a LumiGLO chemiluminescent substrate system.

### Real-time PCR analysis

The 2× Taq PCR Master Mix (KT201) was mixed with complementary DNA samples, forward and reverse primers and double-distilled H_2_O to a final volume of 20 μl. The cycling parameters were as follows: initial denaturation for 2 min at 94 °C; denaturation for 30 s at 94 °C; annealing for 30 s at 60 °C; DNA extension for 45 s at 72 °C, repeating steps 2–4 for 25–30 cycles, followed by a final extension for 5 min at 72 °C. Real-time PCR was performed using the GeneAmp PCR System 9700. Primers are shown in Supplementary Table 5. Agarose gel electrophoresis was performed and the gels were photographed using the ChemiDoc XRS+ System.

### RNA-seq analysis

shRNA-NC and shRNA-*Ddx5* ATDC5 cells were treated with 5 ng ml^−1^ IL-1β and 25 ng ml^−1^ TNF-α (PeproTech) for 6 h; 1 μg RNA per sample was used as the input material for the RNA sample preparations. PCR products were purified using the AMPure XP system and library quality was assessed on the Agilent Bioanalyzer 2100 system. RNA-seq was performed using an Illumina system, following the protocols provided by Illumina for 2 × 150 paired-end sequencing in WuXi NextCODE. Gene expression levels were quantified using featureCounts (v.1.6.3). Differential expression analysis was performed using the DESeq2 R package (v.1.20.0); the resulting *P* values were adjusted using the Benjamini–Hochberg approach for controlling the FDR. Genes with an adjusted *P* < 0.05 found by DESeq2 were classified as differentially expressed. GO and KEGG pathway enrichment analyses were performed using the clusterProfiler R package (v.4.2.0). Clinical disease data were obtained from the Gene Expression Omnibus (GEO) (accession no. GSE114007). On analyzing this dataset, significant differences were observed between two sequencing platforms (Illumina HiSeq 2000 and NextSeq 500). Considering the smaller intra-group differences of sequencing data obtained using the NextSeq 500 platform, we only selected these data for the downstream analysis.

### rMATS methods

The human raw data from the GEO public datasets (accession no. GSE114007) underwent quality control using Trim Galore! (v.0.6.10) and were then mapped to the genome sequence (hg38) using STAR by the rMATS software. The rMATS software (turbo_v4.2.0) was used for the AS event analysis, which included the analysis of five splice types. The number of all identified ASEs, derived from the gene transfer format (GTF) and the number of RNAs was 110,965. The final output, which included only reads that spanned defined junctions was 57,645. We then focused on all (SE).MATS.JC.txt; among these AS events, we identified 624 DASEs (PSI > 4%, FDR < 0.05). The same parameters were used for the RNA-seq raw data (accession no. GSE226983) from shRNA-NC and shRNA-*Ddx5* ATDC5 cells treated with 5 ng ml^−1^ IL-1β and 25 ng ml^−1^ TNF-α. The genome file used mm39.fa and the GTF file used mm39.ncbiRefSeq.gtf. The AS events derived from the GTF and the number of RNA was 94,101. The final output included 68,559 reads that spanned defined junctions. Then, we focused on all SE.MATS.JC.txt; among these ASEs, we identified 3,262 DASEs (PSI > 10%, FDR < 0.05). The rmats2sashimiplotl (v.3.0.0) package was used to produce plots with an annotation file and genomic coordinates of FN1 (*Fn1*) and PLOD2 (*Plod2*) extracted from SE.MATS.JC.txt.

### Single-cell sequencing data processing

Single-cell count matrices were obtained from the GEO (GSE104782) and converted to sparse matrices using Seurat (v.4.0.4) in R. The 1,600 individual chondrocytes obtained from the articular cartilage of ten patients were used in the following analysis. We used sctransform to normalize gene expression and correct batch effects. Principal component analysis was performed with filtered variable genes using RunPCA in the Seurat package. The first 30 principal components were selected for uniform manifold approximation and projection; Louvain clustering was performed using RunUMAP and FindNeighbors/FindClusters in the Seurat package, respectively. The Harmony algorithm (v.1.0) can accurately integrate single-cell data from different platforms and batches. Thus, we integrated these cells from ten patients using RunHarmony. Cell types were determined according to marker gene expression.

### TMT-labeled proteomics assay

shRNA-NC and shRNA-*Ddx5* ATDC5 cells were treated with 5 ng ml^−1^ IL-1β and 25 ng ml^−1^ TNF-α (PeproTech) for 24 h. The detailed steps were as described previously^60^. Cells were washed using cold PBS buffer three times and then lysed with lysis buffer (8 M urea, 50 mM NH_4_HCO_3_, 1× protease and phosphatase inhibitors (Roche)) on ice for 30 min. Then, the lysate was sonicated on ice for 3 min (2 s on and 5 s off) with 30% energy. The solution was centrifuged at 13,400*g* for 10 min and then transferred to a new Eppendorf tube. The concentration of the extracted proteins was detected using bicinchoninic acid assay. Then, proteins were reduced with 5 mM dithiothreitol at 56 °C for 30 min and alkylated with 15 mM IAA in the dark at room temperature for 30 min. The alkylation reaction was quenched with 20 mM IAA at room temperature for 30 min. The concentration of urea in the protein solution was diluted fourfold with 100 mM NH_4_HCO_3_. Then, protein was digested by adding sequencing-grade trypsin (Hualishi) at an enzyme-to-protein ratio of 1:50 (w/w) at 37 °C overnight. Tryptic peptides were desalted using a Sep-Pak tC18 column and vacuum-dried using Speed-Vac before 12-plex TMT labeling. Twelve-plex TMT labeling was performed according to the manual. TMT labeling was performed as reported previously^61^. After labeling, all TMT-labeled samples were pooled at a 1:1 (w/w) ratio and vacuum-centrifuged to dry. The TMT-labeled peptide mixture was desalted using Sep-Pak tC18 columns. The efficiency of TMT labeling was analyzed before deep proteome profiling. Ten micrograms of TMT-labeled peptides were extracted and analyzed using an Easy-NanoLC 1200 system with a Q Exactive HF-X mass spectrometer (Thermo Fisher Scientific). All dried samples were dissolved in loading buffer (0.1% formic acid in water, v/v) and then detected using the Easy-NanoLC 1200 system with a Q Exactive HF-X mass spectrometer.

### Bi-omics joint analysis

DESeq was used to perform differential analysis of both RNA-seq and protein-seq data; the inner_join (dplyr v.1.1.2) function was used to combine the two sets of data based on the same differential gene. The ggplot2 package was used to display key genes in four quadrants. The omics joint display of the GSEA analysis also adopted a similar approach. Pathway enrichment was performed using the KEGG on both sets of data; the inner join function was used to combine the two sets of data based on the same pathways. The KEGG pathways with an enrichment score greater than 0 in both sets of data were screened out.

### AAV intra-articular injection and surgically induced OA model

We chose to use intra-articular injection of recombinant AAV2 for in vivo overexpression of DDX5, and *Fn1*-AS-WT and *Plod2*-AS-WT knockdown. The AAVs pAAV2-CMV-*Ddx5*-3×FLAG-eGFP-WPRE, pAAV2-U6-shRNA (*Plod2*)-CMV-eGFP-WPRE and pAAV2-U6-shRNA(*Fn1*)-CMV-eGFP-WPRE were purchased from Obio Technology. We performed DMM surgery on the right knee of C57BL/6 mice and then injected 1.0 × 10^12^ vg ml^−1^ AAV particles in a 10 µl volume into the knee joint cavity underneath the patella of the right leg using a microsyringe.

### IF and IHC

The knee joint tissues were fixed in a solution of 4% paraformaldehyde, decalcified, dehydrated and finally embedded in paraffin. Serial sections (6 μm) were cut and stained with SO&FG (catalog no. G1371, Solarbio) according to the manufacturer’s instructions. For IF staining, 6-μm sections were permeabilized with 0.2% Triton X-100, blocked with 2% BSA for 1 h and then incubated with primary antibodies overnight at 4 °C. After washing, sections were incubated with anti-rabbit Alexa Fluor 488 (Invitrogen) or anti-mouse Alexa Fluor 568 (Invitrogen) secondary antibodies (1:500 dilution) for 1 h at room temperature. For IHC staining, immunohistochemical antigen retrieval in knee joint tissue is similar to IF staining. After washing, sections were incubated with anti-mouse or anti-rabbit isotype-specific HRP secondary antibodies. Then, sections were processed according to the steps of Tyramide Signal Amplification Vivid Fluorophore Kits. Images were obtained using an Olympus BX51 Fluorescence Microscope. The IF and IHC antibodies used are listed in Supplementary Table 4.

### F-actin staining

ATDC5 cells (shRNA-NC and shRNA-*Ddx5*) were cultured on coverslips and stained with Vari Fluor 488-Phalloidin (catalog no. HY-D1817, MedChemExpress). The experimental procedures strictly followed the manufacturer’s instructions. Images were obtained using a Zeiss LSM 880 confocal laser scanning microscope.

### DDX5 protein purification

Plasmid pMAL-MBP-DDX5-GST purchased from General Biosystems was transformed into BL21 (DE3) cells. Expression of this plasmid was induced by cell culture with 0.2 mM isopropyl β-d-1-thiogalactopyranoside at an OD_600_ of 0.8. After induction, the cell culture was incubated at 16 °C overnight, and cells were collected. Cells were lysed in buffer A containing 50 mM Tris-HCl (pH 8.0) and 300 mM NaCl. MBP-DDX5-GST was first purified from the soluble lysate using glutathione resin and then eluted in buffer B (50 mM Tris-HCl, pH 8.0, 300 mM NaCl, reduced 10 mM glutathione). Then, the initially purified protein was further purified using amylose resin and eluted in buffer C containing 50 mM Tris-HCl, pH 8.0, 300 mM NaCl and 9.2 mM maltose. Finally, the target protein was concentrated using centrifugal ultrafiltration and then exchanged with buffer D, which contained 50 mM Tris-HCl (pH 8.0) and 100 mM NaCl, using centrifugal ultrafiltration. Protein concentration was determined by measuring the ultraviolet absorbance at 280 nm using One Drop OD-1000plus ultra-violet visble spectrophotometer.

### NMR spectroscopy

NMR data were collected on 500 MHz and 850 MHz Bruker spectrometers with 298 K or 310 K cryoprobes. Two-dimensional ^1^H-^1^H total correlation spectroscopy with a mixing time of 80 ms, ^1^H-^13^C HMBC, ^1^H-^13^C heteronuclear single quantum correlation and ^1^H-^1^H NOESY spectra in 10% D_2_O (250-ms mixing time) and 100% D_2_O (50-ms and 250-ms mixing times) were recorded for resonance assignment and structural identification. Water suppression was used in the experiments, either by using gradient-tailored excitation (WATERGATE) for 10% D_2_O samples or a presaturation pulse sequence for 100% D_2_O samples. All datasets were processed and analyzed using Bruker Topspin v.3.6.2. and CcpNmr Analysis v.2.4.2 (ref. ^62^).

### CD spectroscopy assay

Samples that underwent NMR testing were diluted in buffer A (5 mM KPi, pH 6.8, 100 mM KCl) to a concentration of 50 M. Then, CD spectra were obtained at room temperature (25 °C) using a JASCO J-810 spectropolarimeter with a 1-mm path length quartz cuvette. Spectra ranging from 220 to 320 nm were recorded at a scanning rate of 100 nm min^−1^. On average, three scans were taken for each measurement; the baseline was corrected with the same buffer.

### Ribonucleoprotein immunoprecipitation assay and ChIP assay

The Magna RIP RNA-Binding Protein Immunoprecipitation Kit (Sigma-Aldrich) was used to perform RIP assays, according to the manufacturer’s instructions. For the ChIP assays, the ChIP Assay Kit (Beyotime) was used according to the manufacturer’s instructions. The primer sequences and antibodies used are listed in Supplementary Tables 3 and 4, respectively.

### Mouse genotyping

All offspring were genotyped using PCR analysis of gDNA extracted from their toes to screen for DDX5 conditional knockout mice. For PCR, the 2× Taq PCR Master Mix was mixed with toe gDNA, forward and reverse primers, and double-distilled H_2_O to a final volume of 20 μl. The cycling parameters were as follows: initial denaturation for 2 min at 94 °C; denaturation for 30 s at 94 °C; annealing for 30 s at 58 °C; and DNA extension for 30 s at 72 °C, repeating steps 2–4 for 40 cycles, followed by a final extension for 5 min at 72 °C. PCR was performed using the GeneAmp PCR System 9700. The primer sequences used are listed in Supplementary Table 5. Agarose gel electrophoresis was performed and the gels were photographed using the ChemiDoc XRS+ System.

### Gene deletion and silencing

For the deletion of *Ddx5* in ATDC5 cells, pCLenti-U6-shRNA (*Ddx5*)-CMV-Puro-WPRE was purchased from Obio Technology. pCLenti-U6-shRNA-CMV-Puro-WPRE was used as the negative control. ATDC5 cells were transduced in suspension with 500 μl of viral supernatant in a 24-well plate. Cells with *Ddx5* deletion were screened using 10 μg ml^−1^ puromycin. Quantitative PCR with reverse transcription (RT–qPCR) and immunoblot analyses were used to confirm deletion of DDX5 in ATDC5 cells. The shRNA sequence targeting *Ddx5* is shown in Supplementary Table 6.

For the siRNA knockdown experiments, ATDC5 cells were transfected with the indicated siRNAs. After 48 h, protein extracts were isolated from cells and subjected to immunoblot analyses with the indicated antibodies. The sequences of the siRNA primers used in this study are summarized in Supplementary Table 7. Chondrocytes at 30–40% confluency were transfected with 100 nM siRNA and Lipofectamine 2000 (Invitrogen) according to the manufacturer’s protocol. siRNAs targeting *Fn1*-AS-WT and *Plod2*-AS-WT were obtained from Ruibo Bio, while nontargeting (scrambled) siRNA was used as the negative control. The primer sequences used are listed in Supplementary Table 3.

### Statistics and reproducibility

Sample sizes for the mouse studies were similar to those reported in previous publications. The animals used in the experiments were allocated randomly. Data collection and analysis were not performed blind to the conditions of the experiments. No animals or data points were excluded. IF and histology were performed and analyzed in a double-blinded manner. Some graphic elements in Figs. 1a, 2a,c,e, 3a and 6b, Extended Data Fig. 6a,c and Supplementary Figs. 1, 15a, 20d and 21 were created using BioRender. At least three independent experiments obtained similar results, as shown in Figs. 4, 5f,g and 6b, as well as Supplementary Fig. 18a and Extended Data Figs. 6b, 9a,b and 10. Prism v.8.0.1 (GraphPad Software) was used to perform a normal distribution analysis of the data. Then, statistical analyses were conducted using Prism 8. A two-tailed unpaired or paired Student’s *t*-test, and one-way and two-way ANOVA with multiple comparisons, were used for parametric data. Nonparametric data were transformed before parametric analyses. We used nonparametric analysis for converted data that did not adhere to a normal distribution. No statistical methods were used to predetermine sample sizes but our sample sizes are similar to those reported in other publications^52,63^. The results were expressed as the mean ± s.e.m., as indicated in the figure legends. *P* < 0.05 was considered statistically significant.

### Reporting summary

Further information on research design is available in the Nature Portfolio Reporting Summary linked to this article.

### Supplementary information

Supplementary Information Supplementary Figs. 1–21 and Tables 1–7.

Reporting Summary

Supplementary Data 1 Source data for Supplementary Fig. 7.

Supplementary Data 2 Source data for Supplementary Fig. 10.

Supplementary Data 3 Source data for Supplementary Fig. 13.

Supplementary Data 4 Source data for Supplementary Fig. 14.

Supplementary Data 5 Source data for Supplementary Fig. 15.

Supplementary Data 6 Source data for Supplementary Fig. 16.

Supplementary Data 7 Source data for Supplementary Fig. 17.

Supplementary Data 8 Source data for Supplementary Figure 18 (raw NMR data).

Supplementary Data 9 Supplementary data.

Supplementary Table 1 Raw NMR data.

Supplementary Table 2 Proteomic data.

### Source data

Source Data Fig. 1 Unprocessed images, statistical source data.

Source Data Fig. 2 Unprocessed immunoblot, unprocessed images, statistical source data.

Source Data Fig. 3 Unprocessed images, statistical source data.

Source Data Fig. 4 Statistical source data.

Source Data Fig. 5 Unprocessed images, statistical source data.

Source Data Fig. 6 Unprocessed images, statistical source data.

Source Data Fig. 7 NMR data.

Source Data Fig. 8 Unprocessed immunoblot, unprocessed images, statistical source data, NMR data.

Source Data Extended Data Fig. 6 Unprocessed immunoblot, unprocessed images, statistical source data.

Source Data Extended Data Fig. 7 Unprocessed immunoblot, unprocessed images, statistical source data.

Source Data Extended Data Fig. 8 Unprocessed images, statistical source data.

Source Data Extended Data Fig. 9 Unprocessed images.

Source Data Extended Data Fig. 10 Unprocessed images.

## Data Availability

The raw sequence data reported in this article have been deposited in the GEO under accession no. GSE226983. The proteomics raw data have been deposited with a member of the ProteomeXchange consortium iProX (http://www.iprox.org) under project ID IPX0006326000. All data supporting the findings of this study are available within the article or from the corresponding authors upon reasonable request.

## Code availability

The analysis code for this article has been uploaded to the associated website at https://github.com/Portulaca666/AS.code.
