# Supplementary material for: DDX5 inhibits hyaline cartilage fibrosis and degradation in osteoarthritis via alternative splicing and G-quadruplex unwinding
Source: Nat Aging. 2024 May 17;4(5):664–80. doi: 10.1038/s43587-024-00624-0 (PMC11108786; doi:10.1038/s43587-024-00624-0)
Supplement: Supplementary file 1 — Supplementary Figs. 1–21 and Tables 1–7. [file 43587_2024_624_MOESM1_ESM.pdf]

nature aging

Article

https://doi.org/10.1038/s43587-024-00624-0

# DDX5 inhibits hyaline cartilage fibrosis and degradation in osteoarthritis via alternative splicing and G-quadruplex unwinding

In the format provided by the authors and unedited

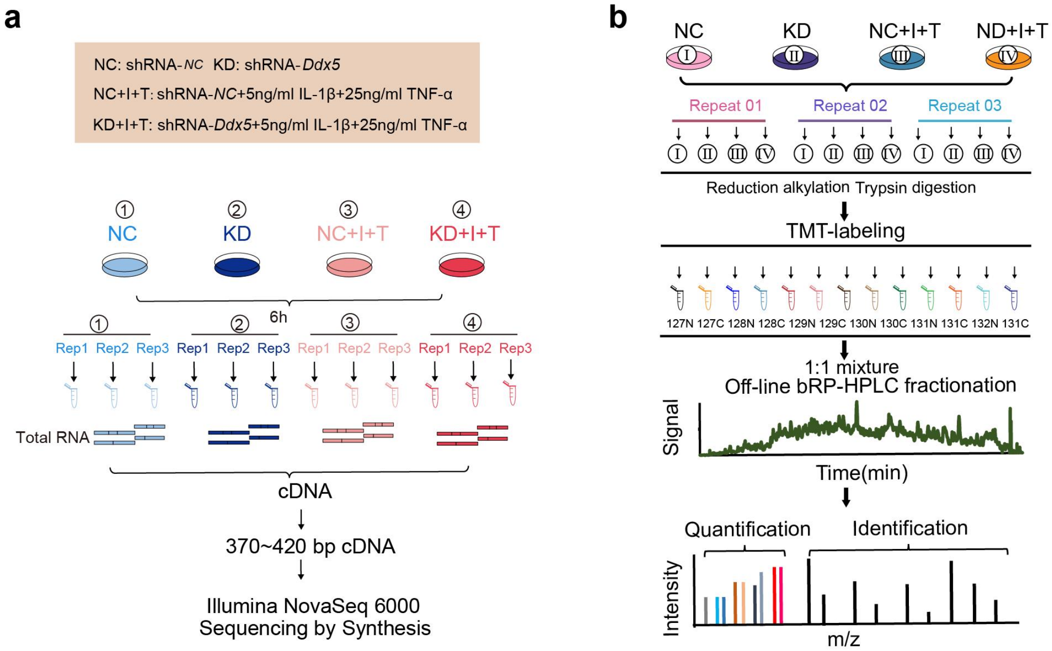


**Supplementary Fig. 1. Technical diagrams of RNA-seq and proteomic experiments. Related to Figure 3. (a)** The technical diagram of RNA-seq experiment design in ATDC5 cells is shown without cytokine stimulation or stimulated with IL-1β combined with TNF-α for 6 h. **(b)** The experiment design in ATDC5 cells with IL-1β combined with TNF-α stimulation for 24 h is shown. For the deep proteome profiling analysis, all the mass spectrometry data were searched against UniProt mouse database (version 20180930) by Proteome Discoverer software (2.5) with the SEQUEST HT search engine. Enzyme specificity was set to trypsin. The 12-plex TMT label on lysine and peptide N-termini, carbamidomethylation of cysteine were set as fixed modifications. The acetylation of protein N-term and oxidation of methionine were set as variable modifications. The maximum missed cleavages were set at 2. The tolerances of MS and MS/MS were set at 20 ppm and 0.02 Da, respectively. The Percolator algorithm in PD was adopted to control peptide spectrum matches at a false discovery rate (FDR) and maximum delta Cn=0.05.

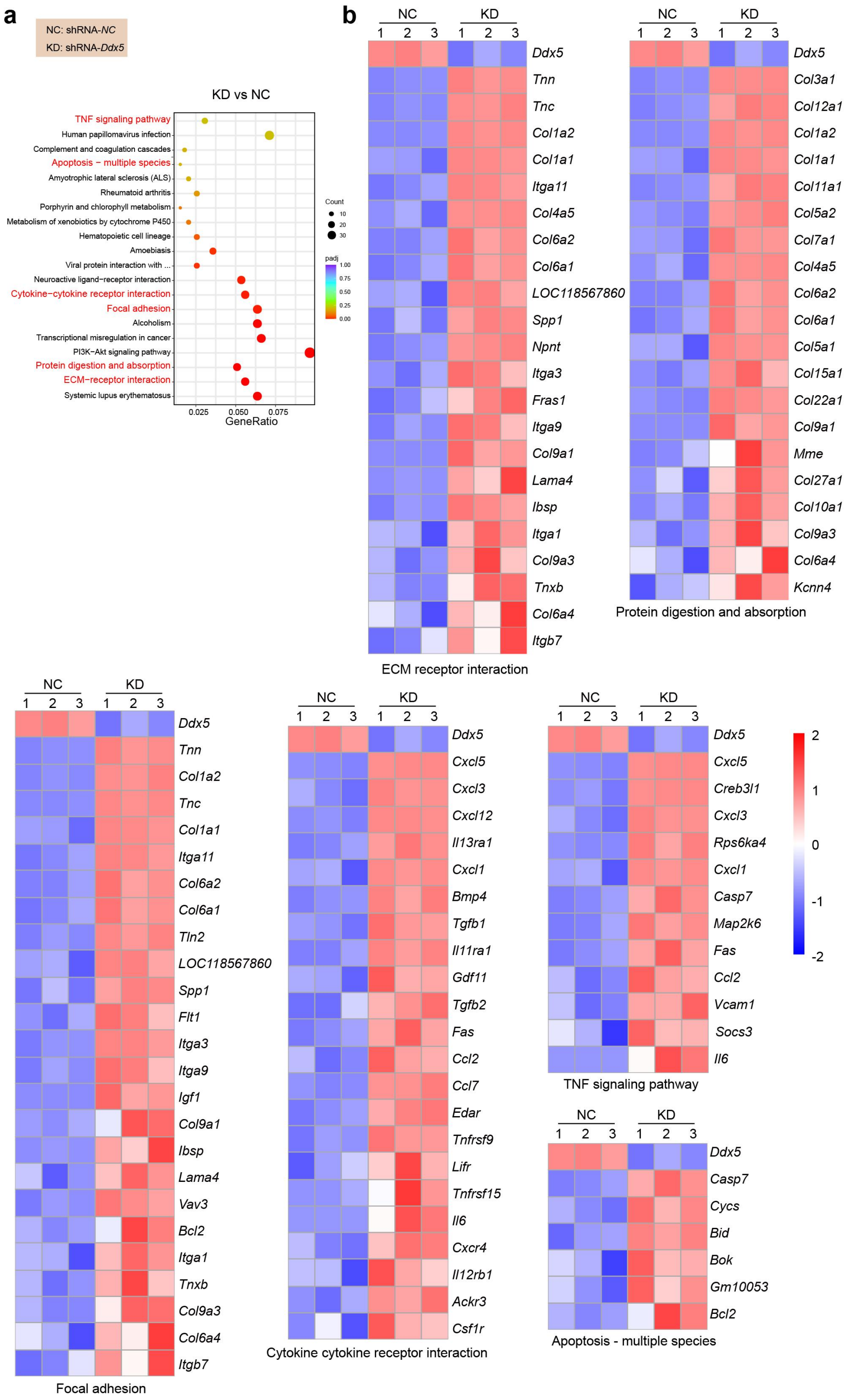


**Supplementary Fig. 2. The knockdown of *Ddx5* results in the upregulation of genes related to inflammation and fibrosis without cytokine stimulation. Related to Figure 3. (a)** Enriched KEGG pathway analysis of upregulated gene (shRNA-Ddx5 vs shRNA-NC) in ATDC5 cells without cytokine stimulation. **(b)** Heat map of gene expression (shRNA-*Ddx5* vs shRNA-*NC*) in the indicated pathway in ATDC5 cells without cytokine stimulation.

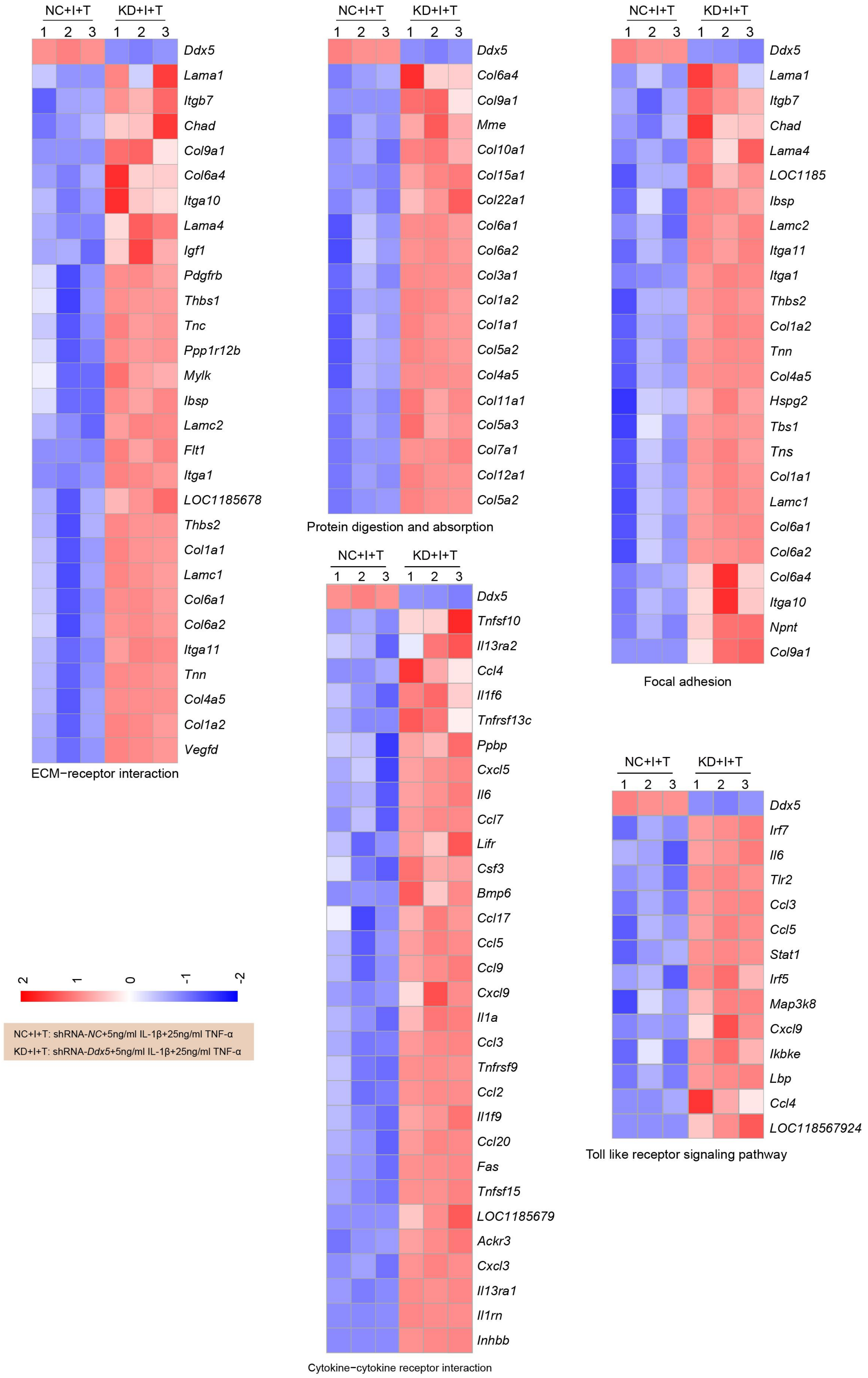


**Supplementary Fig. 3. The knockdown of *Ddx5* results in the upregulation of genes related to inflammation and fibrosis in cytokine stimulation. Related to Figure 3.** Heat map of gene expression (shRNA-*Ddx5* vs shRNA-*NC*) in the indicated pathway in ATDC5 cells (5 ng/ml IL-1β combined with 25 ng/ml TNF-α, 6 h).

**a**

| No.of identified proteins | No.of quantified proteins | KD vs NC | KD+I+T vs NC+I+T |
|---|---|---|---|
| 7279 | 7184 | 715 (Up)<br>510 (Down) | 387 (Up)<br>162 (Down) |

**b**

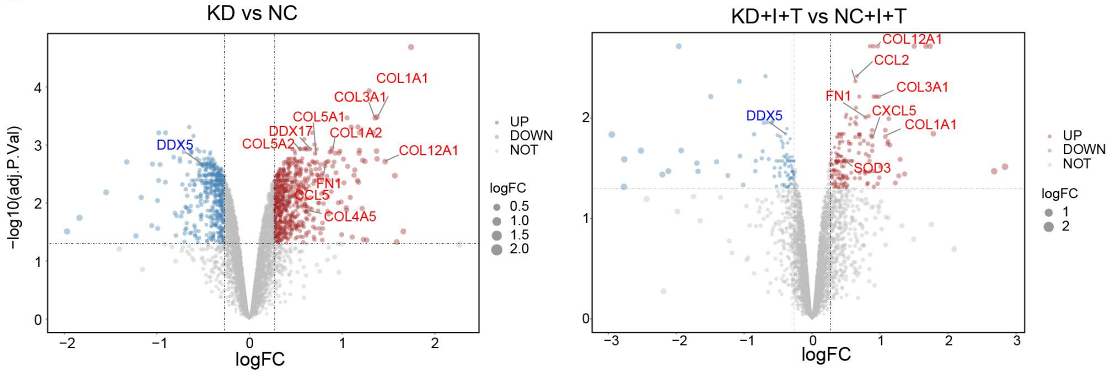


**Supplementary Fig. 4. The knockdown of *Ddx5* results in the upregulation of proteins related to inflammation and fibrosis. Related to Figure 3. (a)** The 12-plex TMT labeling-based quantitative proteomic analysis. The FDR of protein and peptide identification were all set to 1%. The proteins in treated group with 1.2 fold-change were filtered. The statistical analysis was used Student’s *t*-test and the *p*-value was cut-off at 0.05. **(b)** The volcano plot of the protein expression in ATDC5 cells without cytokine stimulation or stimulated with IL-1β combined with TNF-α for 24 h.

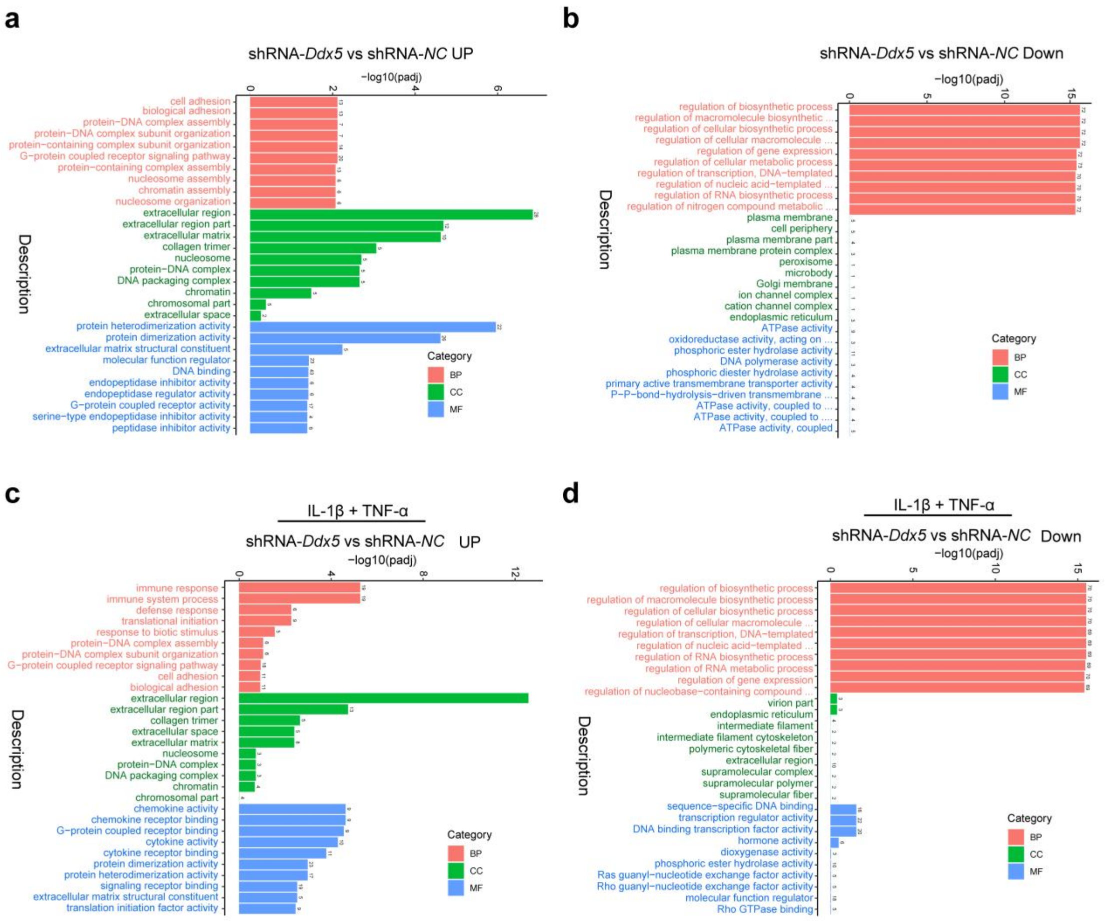


**Supplementary Fig. 5. The GO analysis of upregulated and downregulated genes after *Ddx5* gene knockdown. Related to Figure 3. (a, b)** GO enrichment analysis was proformed on upregulated and downregulated genes (shRNA-*Ddx5* vs shRNA-*NC*) in ATDC5 cells without cytokine stimulation. **(c, d)** GO enrichment analysis was proformed on upregulated and downregulated genes (shRNA-*Ddx5* vs shRNA-*NC*) in ATDC5 cells stimulated with combined with TNF-α stimulation for 6 h. The three main categories: biological process (BP), cellular component (CC), and molecular function (MF).

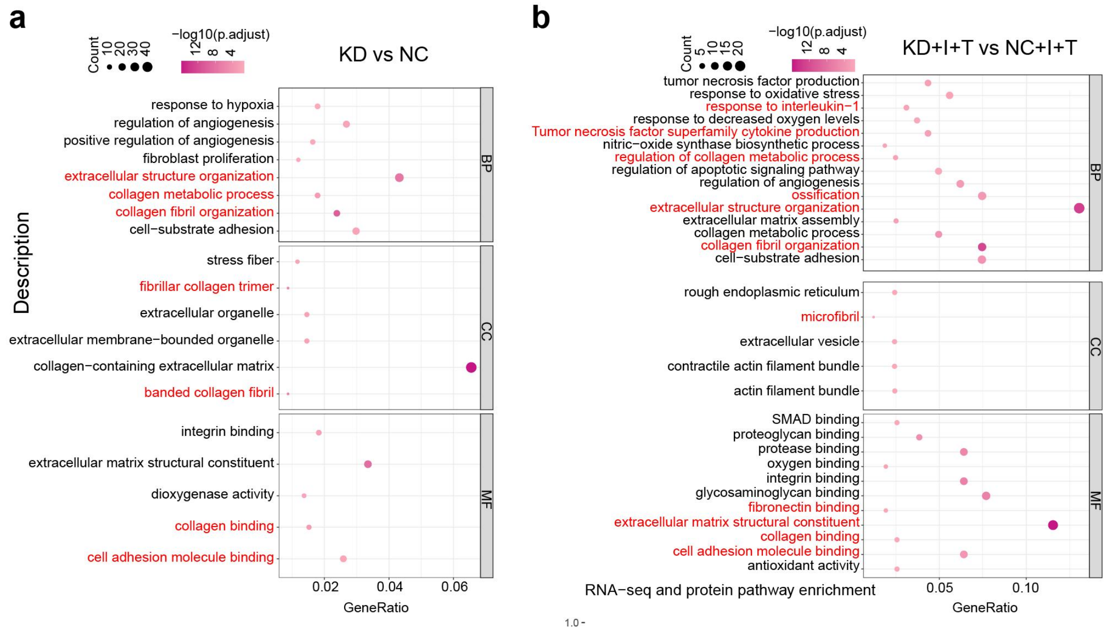


**Supplementary Fig. 6. The proteomic analysis of *Ddx5* knockdown with cytokine stimulation in ATDC5 cells. Related to Figure 3. (a)** GO enrichment analysis was proformed on upregulated proteins (shRNA-*Ddx5* vs shRNA-*NC*) in ATDC5 cells without cytokine stimulation. **(b)** GO enrichment analysis was proformed on upregulated proteins (shRNA-*Ddx5* vs shRNA-*NC*) in ATDC5 cells in ATDC5 cells with IL-1β combined with TNF-α stimulation for 24 h.

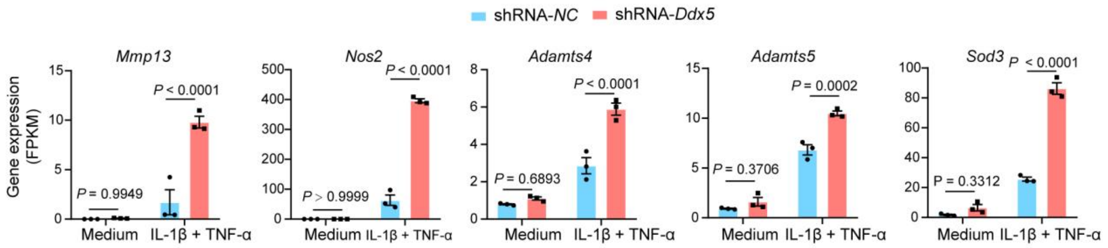


**Supplementary Fig. 7. The knockdown of *Ddx5* results in upregulation genes related to cartilage degradation. Related to Figure 3.** The expression of cartilage degradation-related genes *Mmp*13, *Nos2*, *Adamts* (4, 5), and *Sod3* in shRNA-*NC* and shRNA-*Ddx5* ATDC5 cells stimulated with 5 ng/ml IL-1β combined with 25 ng/ml TNF-α or not for 6 h (n = 3 biologically independent experiments). All data are presented as the mean ± SEM. Two-way ANOVA with Sidak's multiple comparisons test was conducted.

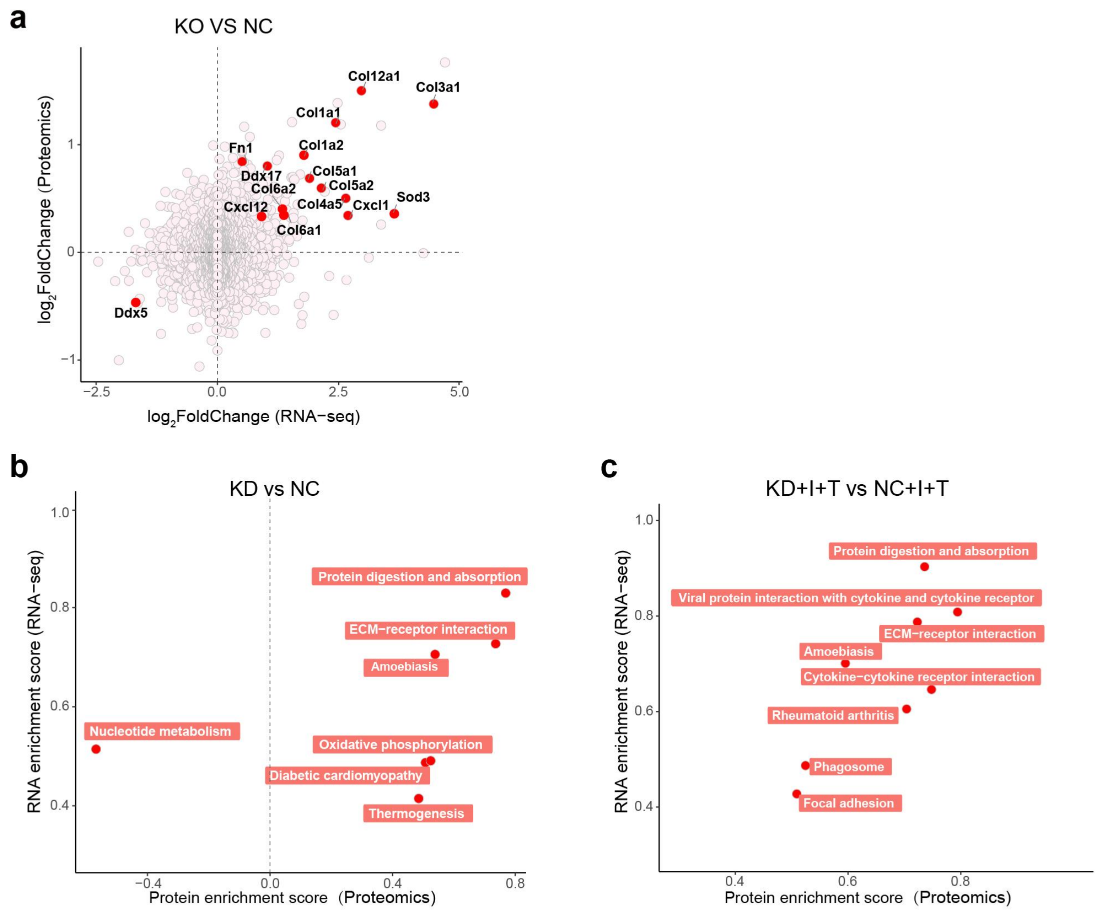


**Supplementary Fig. 8. The common genes and pathways are enriched in both RNA-seq data and proteomic data pathway analysis. Related to Figure 3.** The Bi-omics joint pathway analysis was conducted in ATDC5 cells without cytokine stimulation or stimulated with IL-1β combined with TNF-α.

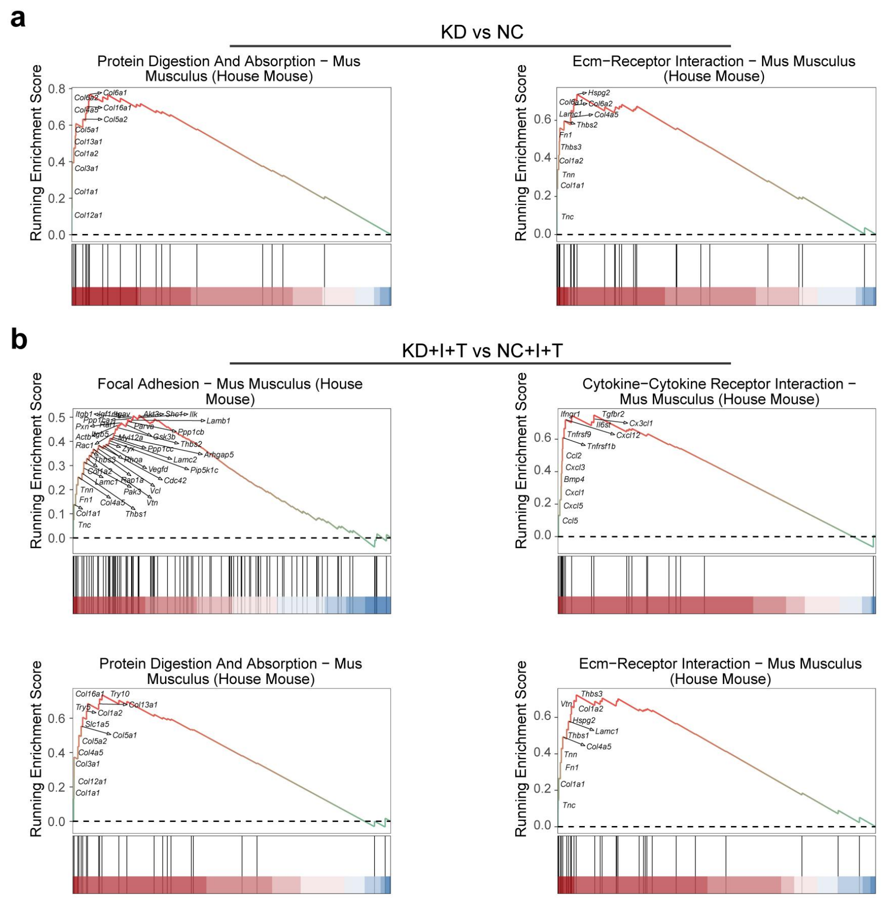


**Supplementary Fig. 9. The GSEA analysis from proteomics data. Related to Figure 3.** ATDC5 cells were stimulated with IL-1β combined with TNF-α for 24 h or without cytokine stimulation.

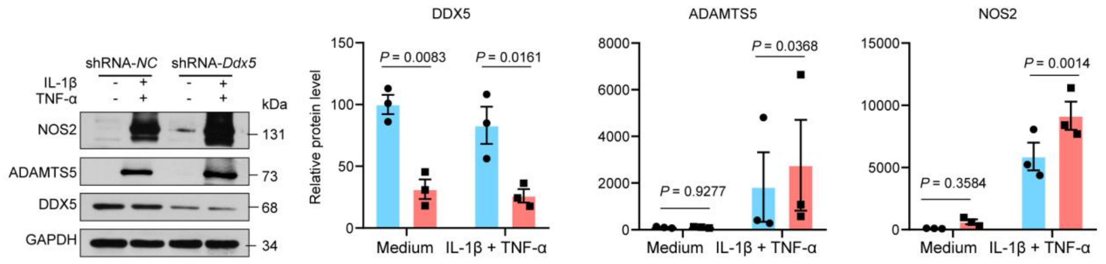


**Supplementary Fig. 10. The knockdown of *Ddx5* results in upregulation proteins related to cartilage degradation. Related to Figure 4.** Western bolt analysis of cartilage degradation-related proteins NOS2 and ADAMTS5 in shRNA-*NC* and shRNA-*Ddx5* ATDC5 cells stimulated with 5 ng/ml IL-1β combined with 25 ng/ml TNF-α or not for 24 h. The density of NOS2 and ADAMTS5 was measured by Image

J software (n = 3 biologically independent experiments). All data are presented as the mean ± SEM. Two-way ANOVA with Sidak's multiple comparisons test was conducted.

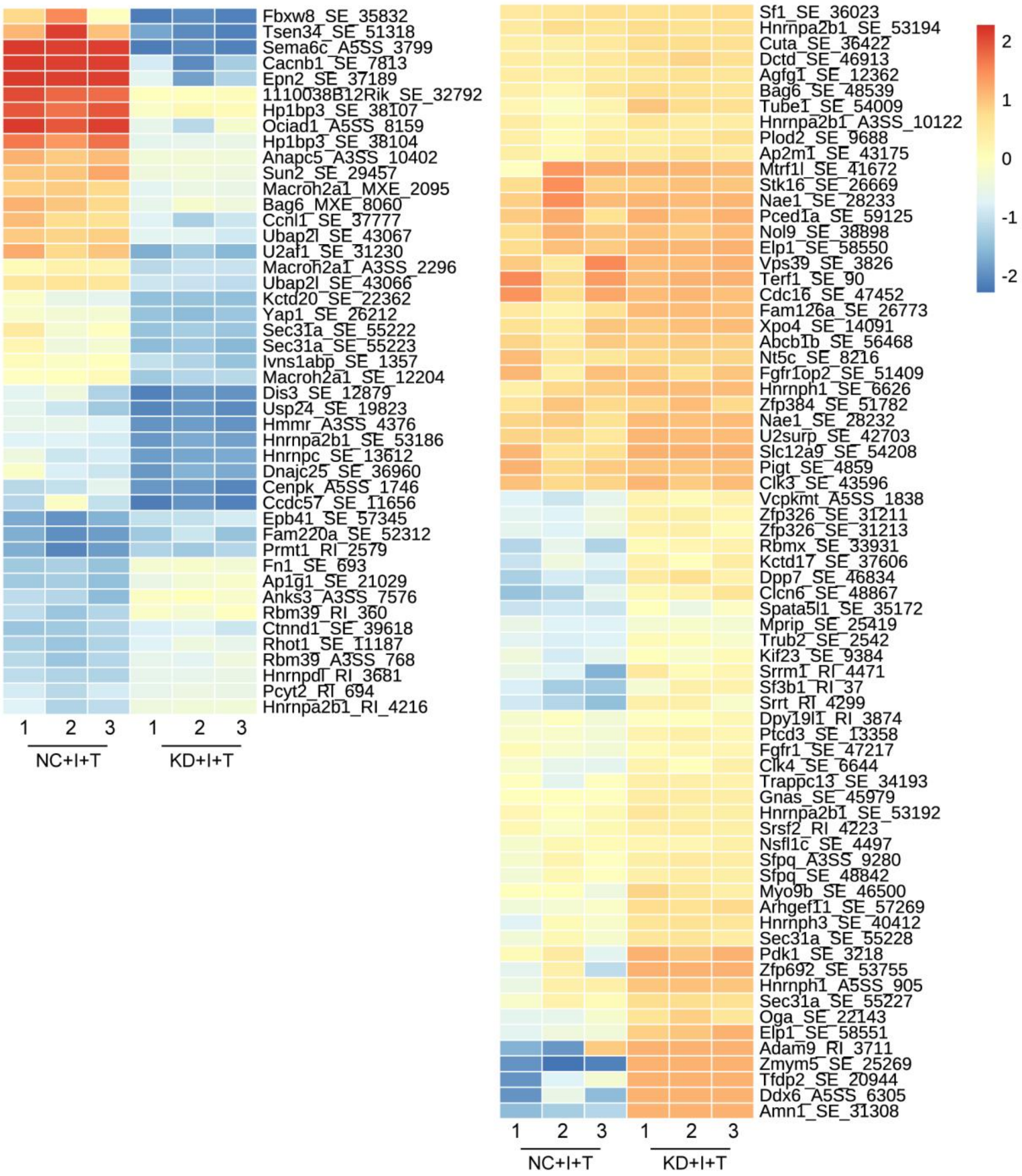


**Supplementary Fig. 11. The most significant differential alternative splicing events between shRNA-*NC and* shRNA-*Ddx5* ATDC5 cells. Related to Figure 5.** The shRNA-*NC* and shRNA-*Ddx5* ATDC5 cells stimulated with IL-1β combined with TNF-α for 6 h. Numbers of alternative splicing events in each category upon *Ddx5* deletion are indicated. The screening cut off delta PSI > 20% and FDR < 1× $10^{-8}$.

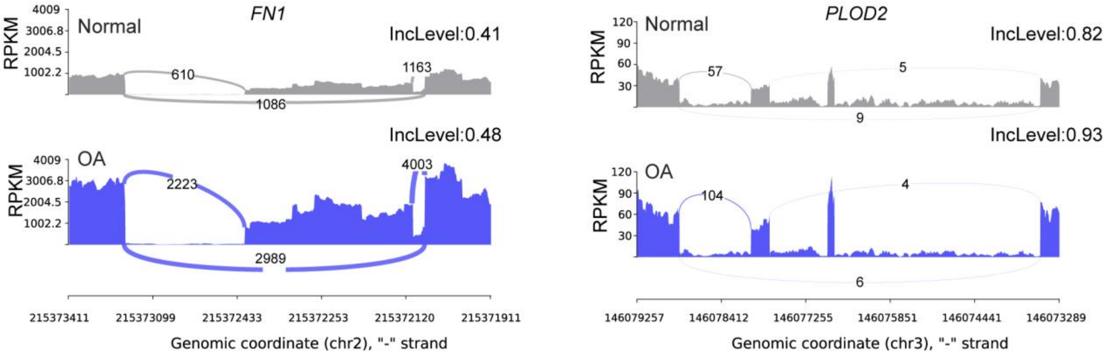


**Supplementary Fig. 12. The *FN1* and *PLOD2* pre-mRNAs produce SE-type splicing by rMATS analysis of clinical OA cartilage samples. Related to Figure 5.** The replicate multivariate analysis of transcript splicing (rMATS) software was used to analyze the splicing differences of the skipped exon (SE) in *FN1* and *PLOD2* genes between 10 human OA cartilage tissues and 10 normal controls (GSE114007).

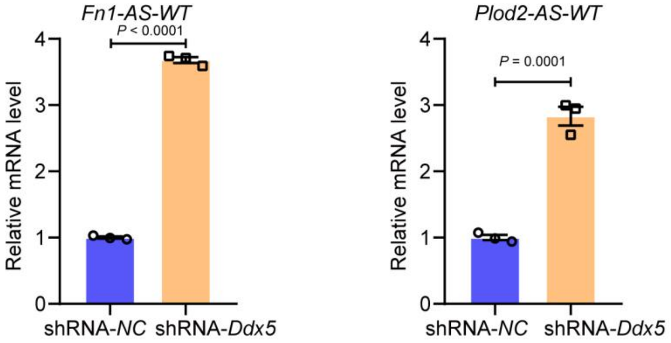


**Supplementary Fig. 13. The mRNA levels of *Fn1-AS-WT* and *Plod2-AS-WT* in ATDC5 cells. Related to Figure 5.** The ATDC5 cells (shRNA-*NC* and shRNA-*Ddx5*) were stimulated with IL-1β combine with TNF-α for 6 h (n = 3 repetition). All data are presented as the mean ± SEM. Student's *t*-test (unpaired) was conducted.

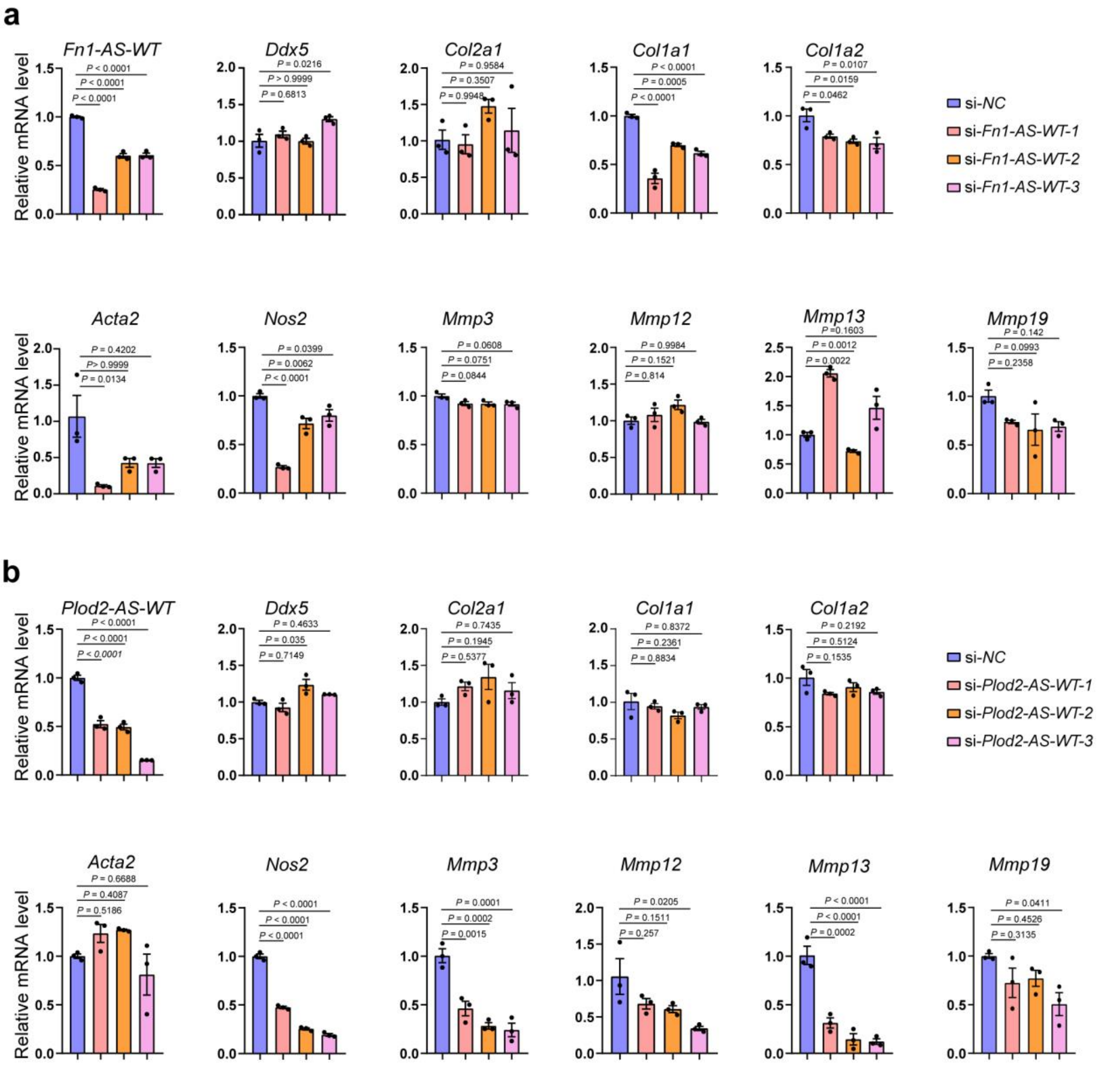


**Supplementary Fig. 14. The knockdown of *Fn1-AS-WT* significantly decreases fibrosis by inhibiting *Col1a* (1, 2) and *Acta2* expression, while the knockdown of *Plod2-AS-WT* markedly reduces ECM degradation by inhibiting *Nos2* and *Mmps* (3, 12, 13, 19) expression. Related to Figure 6.** The knockdown efficiency of siRNA and illustrated genes expression were analyzed by qPCR in primary mouse chondrocytes transfected with siRNA-*Fn1-AS-WT*-1/2/3- **(a)** or siRNA-*Plod2-AS-WT*-1/2/3 **(b)** for 24 h (n = 3 repetition). All data are shown as the mean ± SEM. **a**-*Acta2*, Kruskal-Wallis with Dunn's multiple comparisons test. One-way ANOVA with Tukey's multiple comparisons test was conducted in others.

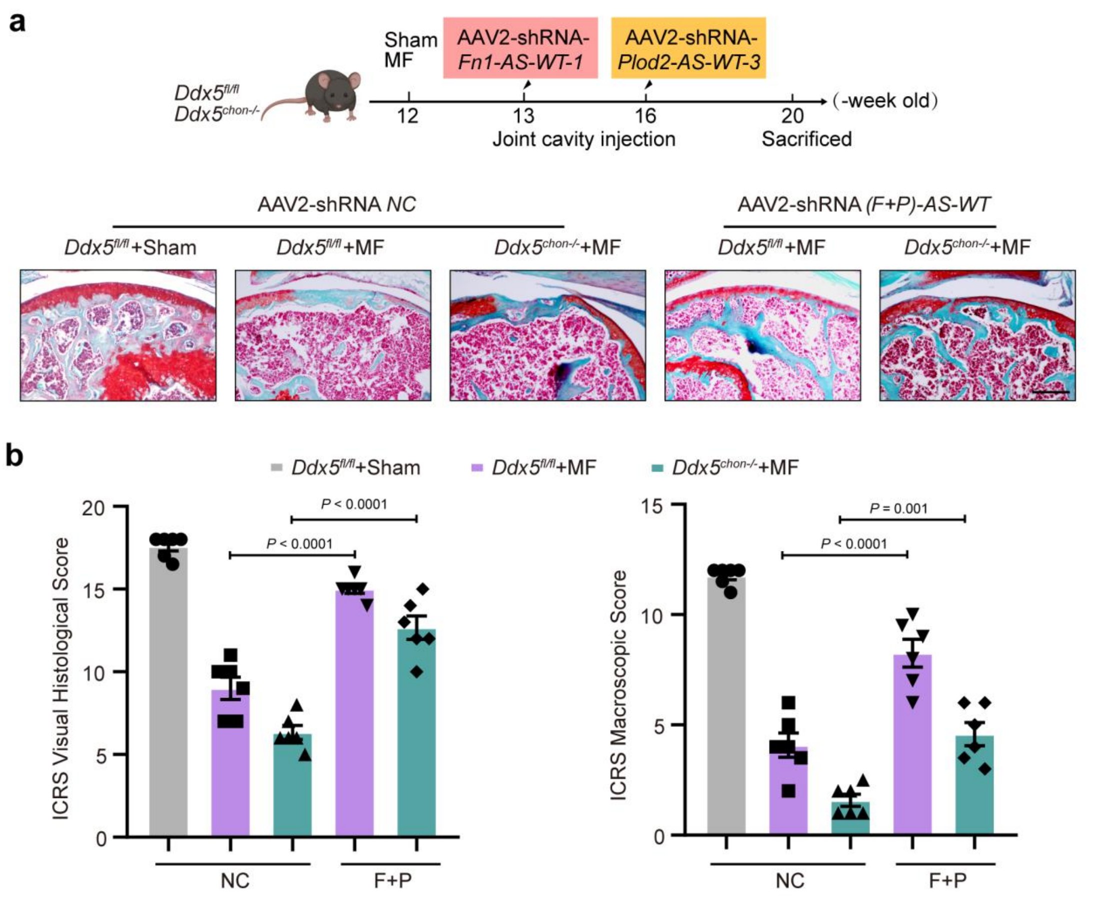


**Supplementary Fig. 15. The knockdown of *Fn1-AS-WT* and *Plod2-AS-WT* variants reverses the aggravating phenotype of microfracture (MF) surgery induced by DDX5 deficiency. Related to Figure 6. (a)** Representative SO&FG staining image from sham and MF mice intra-articular injected with AAV2-NC or AAV2-shRNA-(*Fn1*+*Plod2*)-AS-WT. **(b)** The sham and defect cartilage 8 weeks after MF surgery were shown. The ICRS Visual Histological Score and International Cartilage Repair Society (ICRS) macroscopic score of regenerated tissues analysis was analyzed (n = 6 mice/group). Scale bars, 100 μm. All data are the mean ± SEM. One-way ANOVA with Tukey's multiple comparisons test was conducted.

All data are presented as the mean ± SEM. *$P < 0.05$, **$P < 0.01$, ***$P < 0.001$ by one-way ANOVA.

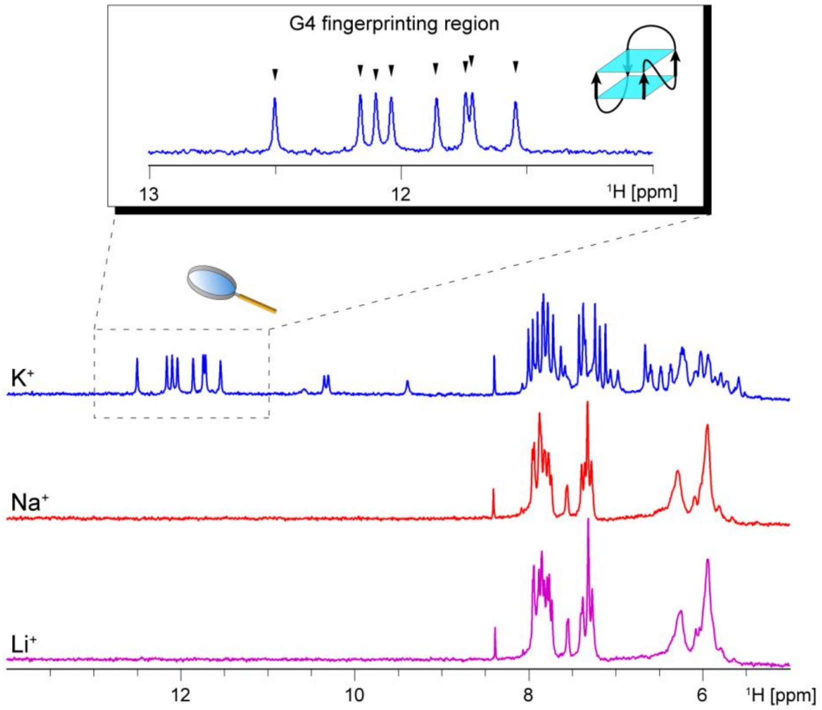


**Supplementary Fig. 16. One-dimensional NMR spectra of position-689 in $Li^+$ (magenta), $Na^+$ (red), or $K^+$ (blue) solutions, respectively. Related to Figure 7.** The G4 fingerprinting region (11~13 ppm) of position-689 in $K^+$ is magnified in the inset. Experimental conditions: 0.2 mM position-689, 20 mM Tris-HCl (pH 6.8), 100 mM LiCl/NaCl/KCl, 310 K.

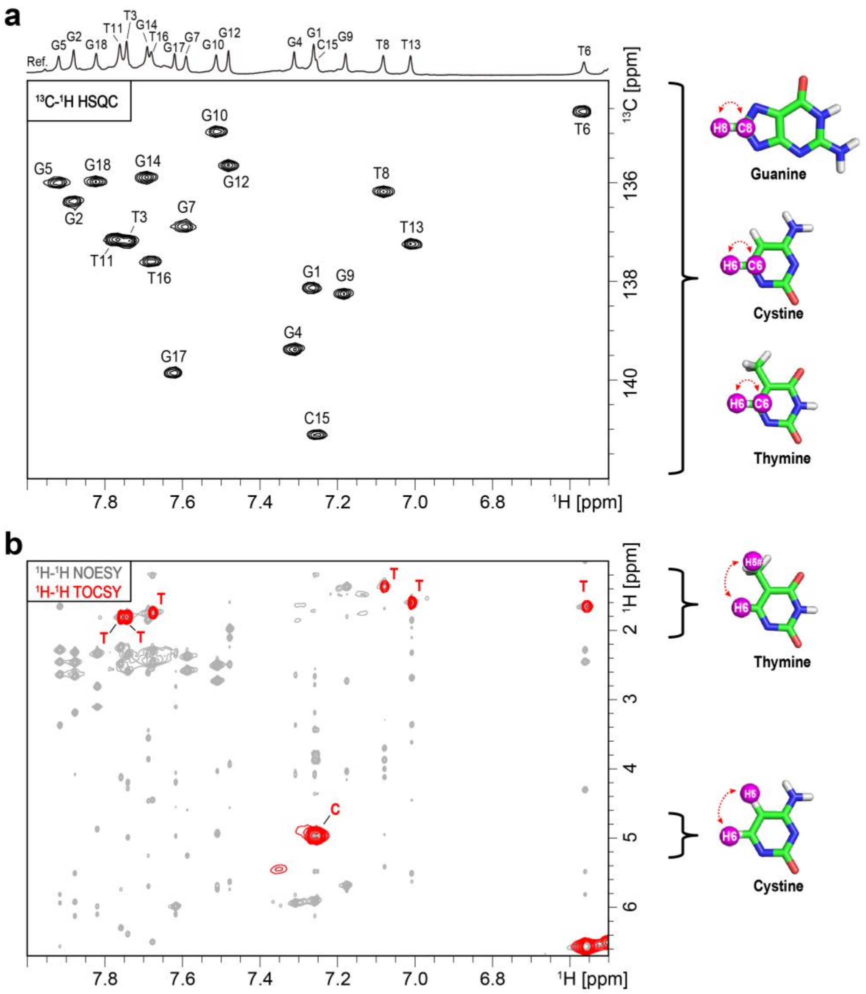


**Supplementary Fig. 17. Aromatic-proton assignments of position-689 using two-dimensional through-bond NMR experiments. Related to Figure 7.** The two protons correlated through bonds are labelled with magenta beads in the bases and shown on the right. **(a)** The expanded base H8/H6 proton region of the $^{1}$H-$^{13}$C HSQC spectrum. One-dimensional projections of aromatic base protons are shown on the top. **(b)** H6 proton of thyine and cystine assignments using the two-dimensional spectra $^{1}$H-$^{1}$H NOESY (coloured gray) and $^{1}$H-$^{1}$H TOCSY (coloured red). Cross-peaks H6-CH3 (thyine) and H6-H5 (cystine) are labelled on the spectum. Experimental conditions: 4 mM position-689, 20 mM K-Pi buffer (pH 6.8), 100 mM KCl and 298 K.

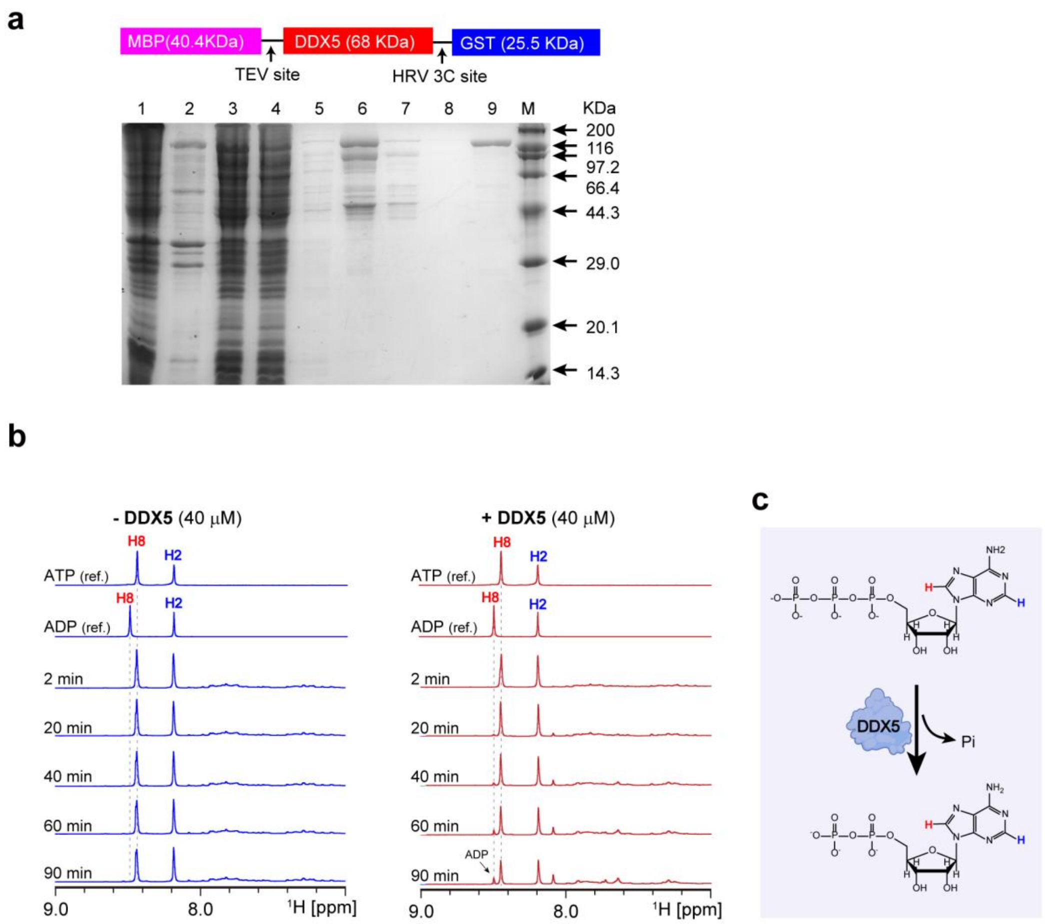


**Supplementary Fig. 18. NMR traces that partial DDX5 is used to catalyze the formation of ADP from ATP. Related to Figure 8. (a)** DDX5 protein purification. The numbers 1-9 on the gel represent the followimg: 1. Bacteria liquid after ultrasonic lysis; 2. Precipitate after centrifugation; 3, Supernatant after centrifugation; 4. GST puncture liquid; 5. GST rinse liquid; 6. GST elution liquid; 7. MBP puncture liquid; 8. MBP rinse liquid; 9. MBP elution liquid; M represents protein marker (broad). **(b)** The aromatic region of one-dimensional NMR spectra are shown. Experimental conditions: 0.2 mM position-689, 0 μM or 40 μM DDX5 , 20 mM Tris-HCl (pH 8.0), 10 mM KCl, 2.5 mM $MgCl_2$, 2 mM DTT, and 310 K. The peaks of H8 and H2 protons are coloured red and blue, respectively. **(c)** A schematic illustration of DDX5 catalyzing the formation of ADP from ATP.

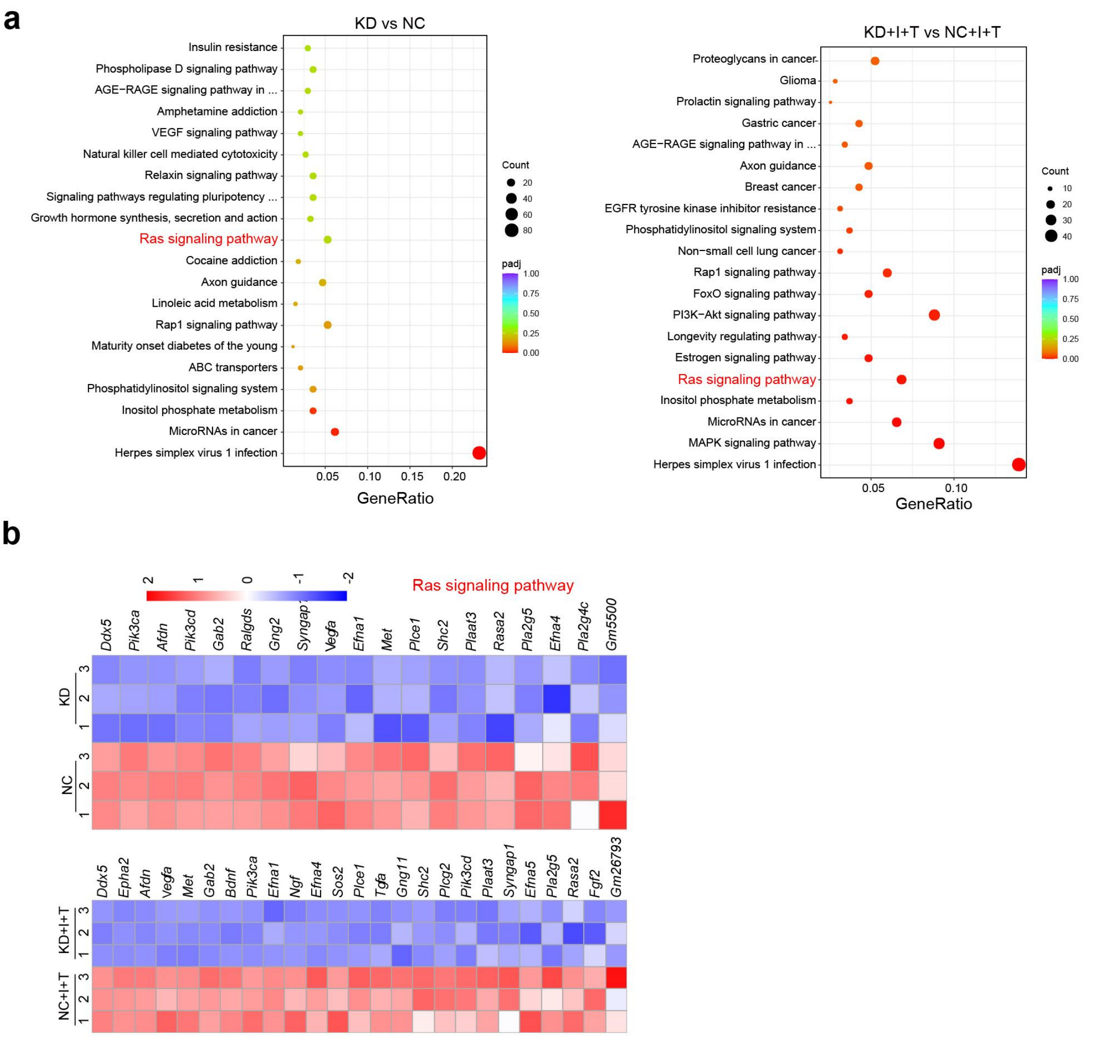


**Supplementary Fig. 19. The KEGG analysis of downregulated genes after *Ddx5* knockdown. Related to Figure 3 and Discussion. (a)** Downregulated gene-enriched KEGG pathway analysis (shRNA-*Ddx5* vs shRNA-*NC*) in ATDC5 cells without cytokine stimulation or stimulated with IL-1β combined with TNF-α for 6 h. **(b)** Heat map of gene expression (shRNA-*Ddx5* vs shRNA-*NC*) in Ras signaling pathway in ATDC5 cells without cytokine stimulation or stimulated with IL-1β combined with TNF-α for 6 h.

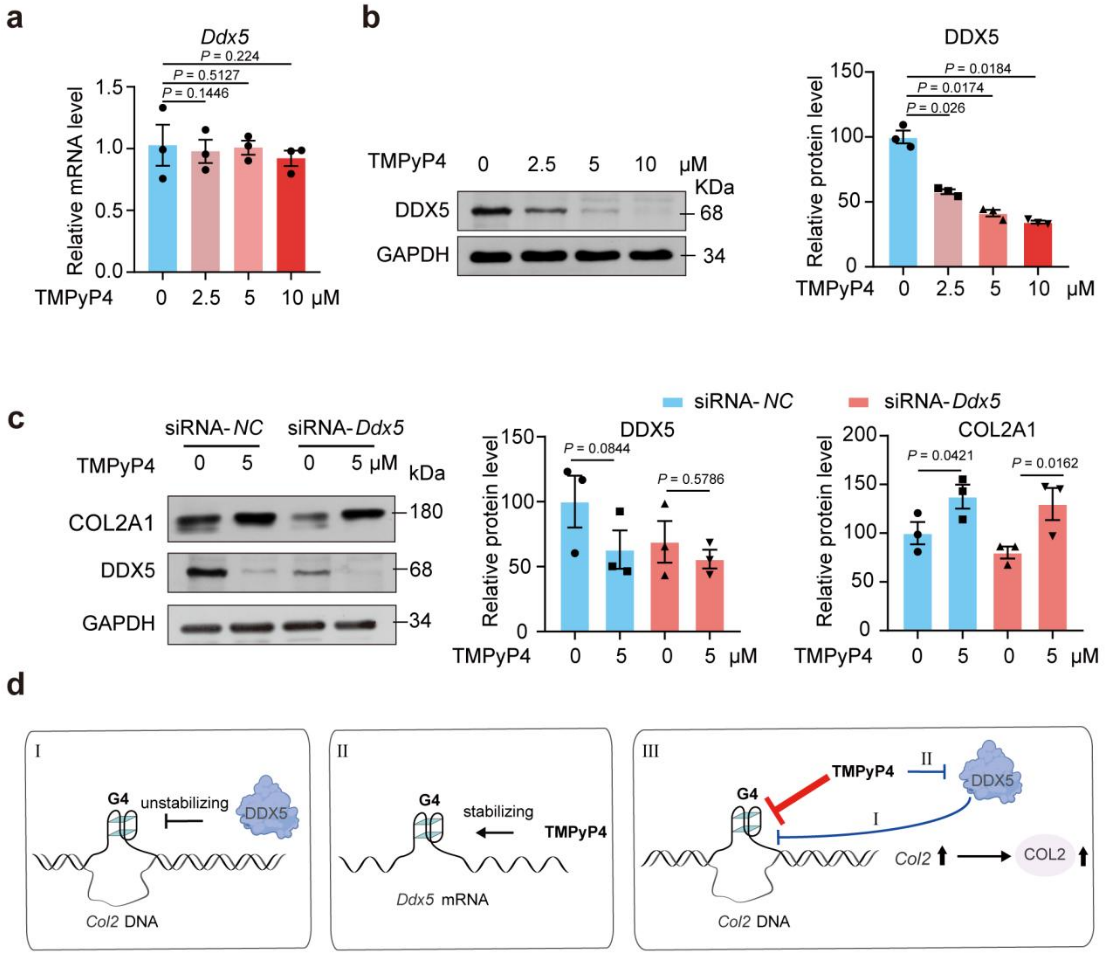


**Supplementary Fig. 20. The effect of TmPyP4 on COL2 in primary mouse chondrocytes. Related to Discussion. (a)** QPCR analysis of *Ddx5* in primary mouse chondrocytes was conducted with indicated doses of TMPyP4 for 12 h (n = 3 repetition). **(b)** Western bolt analysis of DDX5 in primary mouse chondrocytes was conducted with indicated doses of TMPyP4 for 24 h (n = 3 biologically independent experiments). Image J software for the density measurements. **(c)** Western bolt analysis of COL2A1 and DDX5 in primary mouse chondrocytes (siRNA-*NC* vs siRNA-*Ddx5*) was conducted with indicated doses of TMPyP4 for 24 h (n = 3 repetition). Image J software for the density measurements. **(d)** A schematic illustration for TMPyP4 regulation of *Col2*G4-DNA and *Ddx5*G4-mRNA. The *Col2*G4-DNA is unstabilized by DDX5 (I). The *Ddx5*G4-mRNA is stabilized by TMPyP4 (hypothesis) (II). The impact of the downregulated DDX5 on *Col2*G4 was

completely blocked by TMPyP4 as it continuously and directly unwound *Col2*G4 (III). All data are presented as the mean ± SEM. One-way ANOVA with Tukey's multiple comparisons test (a,b), and two-way ANOVA with Sidak's multiple comparisons test were conducted (c).

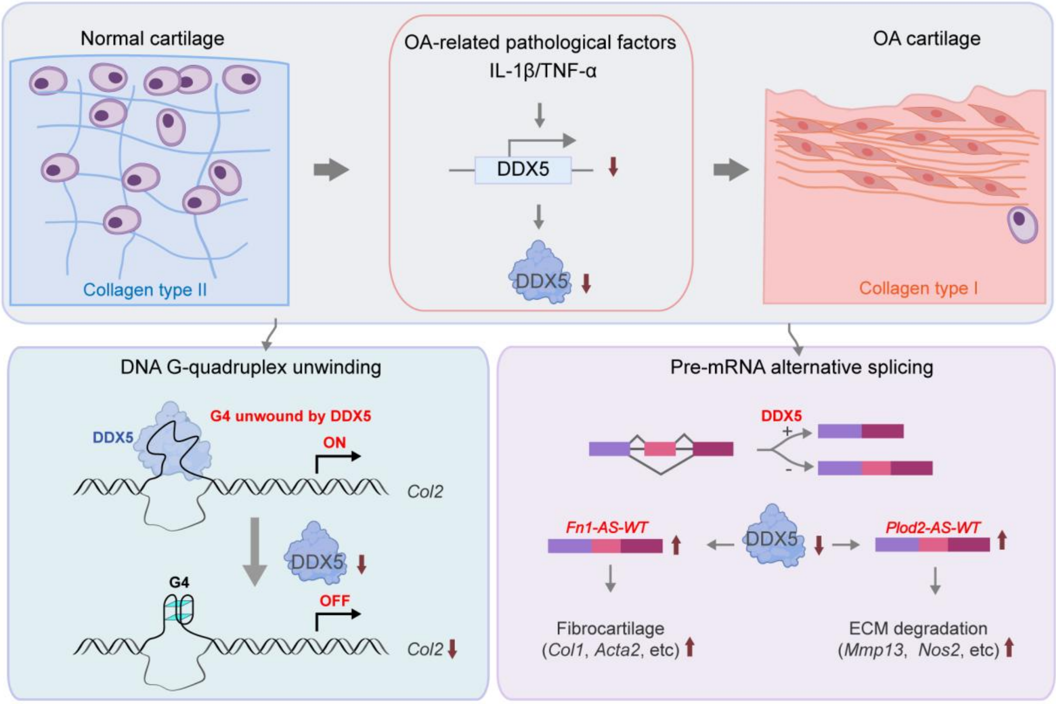


**Supplementary Fig. 21. A mechanistic model is proposed to explain how DDX5-mediated alternative splicing and G4 unwinding to inhibit hyaline cartilage fibrosis and degradation in OA.** The expression of DDX5 is significantly downregulated in response to OA-related pathological factors. The decrease of DDX5 exacerbates hyaline cartilage fibrosis in two ways. On the one hand, the reduction of DDX5 results in an increased G4 in the *Col2* promoter region, leading to a decrease in expression level of COL2. This decrease indicates a loss of the hyaline cartilage phenotype (left). On the other hand, the loss of DDX5 leads to a decrease in the splicing of Fn1 and Plod2 pre-mRNAs. Consequently, the upregulated *Fn1-AS-WT* and *Plod2-AS-WT* isoforms promote fibrotic cartilage phenotype and ECM degradation, respectively (right). Therefore, inducing the upregulation of DDX5 in chondrocytes can protect the integrity of articular cartilage and prevent cartilage fibrosis and degeneration.

**Supplementary Table 1. $^{1}$H NMR detection of the putative quadruplex sequences in the *Col2* promoter.**

| Position | Length | Sequence (5'→3') | G-Score | $^{1}$H NMR Spectra [b] |
|---|---|---|---|---|
| 689 | 18 | GGTGGTGTGGTGTGC TGG | 16 | 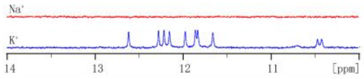 |
| 736 | 26 | GGGAGTGTCGGTCA GGTCCATAGAGG | 16 | 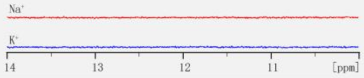 |
| 1000 | 22 | GGGCTAAGGTGGGG GGGTGGGG | 36 | 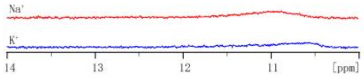 |
| 1040 | 23 | GGCTGGAGGTCCAG GGATAGGGG | 21 | 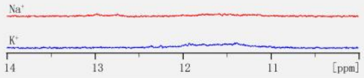 |
| 1199 | 30 | GGCCAGCGGTTGCTC TATCCAGGCTAAGGG | 20 | 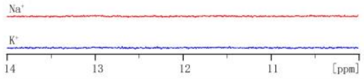 |
| 1461 | 20 | GGATGGGGATGGG TTAGGG | 21 | 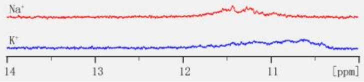 |
| 1504 | 20 | GGTGGGAGCTCACGG GCAGG | 16 | 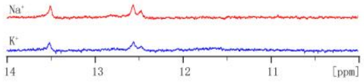 |
| 1541 | 15 | GGAAGGCAGGTCTGG | 20 | 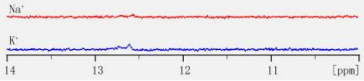 |
| 1796 | 20 | GGTTAGAGGGGGCAG TGTGG | 16 | 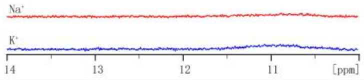 |
| 1827 | 15 | GGGGGCGACCGGAGG | 17 | 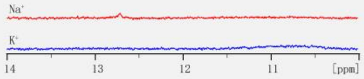 |
| 1873 | 26 | GGGCACATTGGGGGC GGGAAGCTGGG | 39 | 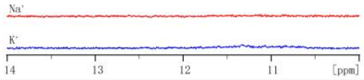 |
| 1908 | 17 | GGCGACTGGCCTTGG | 16 | 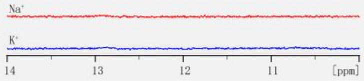 |
| 1949 | 20 | GGGCGGGCTCCGGG GGCGGG | 39 | 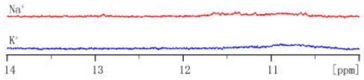 |
| 1979 | 25 | GGTTACAGCCCCGC GGGGGGCTAGG | 11 | 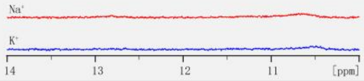 |

[a] Experimental conditions: 0.2 mM position-689, 20 mM K-Pi buffer (pH 6.8), 100 mM KCl, and 298 K.
[b] The region of imino protons (10 ~ 14 ppm) are shown.

**Supplementary Table 2. Clinical and demographic characteristics of the study population**

| Patient ID | OA1 | OA2 | OA3 | OA4 | OA5 | OA6 |
|---|---|---|---|---|---|---|
| Age(years) | 61 | 84 | 77 | 68 | 73 | 82 |
| Gender | Female | Female | Male | Female | Female | Female |
| Weight (Kg) | 66 | 50 | 70 | 89 | 75 | 65 |
| TKA | Left | Right | Left | Right | Right | Right |
| CRP (mg/dL) | 3 | 2.3 | 1.5 | 28.8 | 7.8 | 9.5 |
| ESR (mm/h) | 10 | 14 | 10 | 16 | 38 | 20 |
| WBC (109/L) | 8.9 | 4.6 | 6 | 6.8 | 5.7 | 7.4 |
| Lymphocyte (109/L) | 3.4 | 1.4 | 2.1 | 1.9 | 1.1 | 3.4 |
| Monocyte (109/L) | 0.6 | 0.3 | 0.4 | 0.5 | 0.6 | 0.6 |
| Blood glucose (mmol/L) | 4.06 | 4.76 | 5.37 | 5.13 | 4.82 | 5.03 |
| ALT (U/L) | 26.2 | 17.6 | 11.7 | 12.8 | 15.8 | 12.7 |
| AST (U/L) | 23.8 | 24.4 | 15.7 | 17.6 | 24.5 | 14.6 |
| Total Protein (g/L) | 61.6 | 67.3 | 67.2 | 67.8 | 42.4 | 69.4 |
| Serum albutnin (g/L) | 38.7 | 40.9 | 39.2 | 39.8 | 30.2 | 39.4 |
| BUN (mmol/L) | 6 | 4.9 | 6.7 | 3.6 | 6.3 | 6.4 |
| Cre (μmol/L) | 48 | 56 | 73 | 45 | 55 | 64 |
| TT (s) | 18.8 | 17.5 | 17.2 | 17.1 | 16.8 | 16.6 |
| APTT (s) | 24.1 | 27.4 | 27.1 | 28 | 25.7 | 23.4 |
| INR | 0.91 | 0.89 | 0.92 | 0.94 | 0.95 | 0.94 |

**Supplementary Table 3. Primer sequences used in qRT-PCR.**

| Gene | Primer |
|---|---|
| Mouse *Fn1-AS-WT* | Forward primer: GCCTGGAGTACAACGTCAGT<br>Reverse primer: GGAAGAGTTTAGCGGGGTCC |
| Mouse *Plod2-AS-WT* | Forward primer: ACTCCCCTACTCCGGAAACA<br>Reverse primer: GCAGTTGATATCAGCCGTCCA |
| Mouse *Mmp12* | Forward primer: CTGCTCCCATGAATGACAGTG<br>Reverse primer: AGTTGCTTCTAGCCCAAAGAAC |
| Mouse *Mmp13* | Forward primer: CTTCTTCTTGTTGAGCTGGACTC<br>Reverse primer: CTGTGGAGGTCACTGTAGACT |
| Mouse *Mmp3* | Forward primer: TTGACTCAAGGGTGGATGCTGTCT<br>Reverse primer: GCACATGCTGAACAAAGCACTTCC |
| Mouse *Acta2* | Forward primer: GTCCCAGACATCAGGGAGTAA<br>Reverse primer: TCGGATACTTCAGCGTCAGGA |
| Mouse *Col2α1* | Forward primer: CAGGATGCCCGAAAATTAGGG<br>Reverse primer: ACCACGATCACCTCTGGGT |
| Human *GAPDH* | Forward primer: CGTCTTCACCACCATGGAGA<br>Reverse primer: CGGCCATCACGCCACAGTTT |
| Mouse *Gapdh* | Forward primer: AGGTCGGTGTGAACGGATTTG<br>Reverse primer: TGTAGACCATGTAGTTGAGGTCA |

| | |
|---|---|
| Human *DDX5* | Forward primer: GCCATGTCGGGTTATTCG<br>Reverse primer: GGTTTCCAAACTTCTTTCCAGA |
| Mouse *Ddx5* | Forward primer: TGTGATTGCAAGGCAGAACTTT<br>Reverse primer: TGGCCAGCCCTGAGCTT |
| Mouse *Col1a1* | Forward primer: TAAGGGTCCCCAATGGTGAGA<br>Reverse primer: GGGTCCCTCGACTCCTACAT |
| Mouse *Col1a2* | Forward primer: GTAACTTCGTGCCTAGCAACA<br>Reverse primer: CCTTTGTCAGAATACTGAGCAGC |
| Mouse *Col2α1*-promoter 515-759 | Forward primer: CCCACCCTAGACTGGTTGAA<br>Reverse primer: TCTATGGACCTGACCGACAC |
| Mouse *Col2α1*-promoter 1470-1590 | Forward primer:ATGGGTTAGGGAGGCTGT<br>Reverse primer: CGTTGTCCAGAATCCTAAA |
| Mouse *Nos2* | Forward primer: GGAGTGACGGCAAACATGACT<br>Reverse primer: TCGATGCACAACTGGGTGAAC |
| Mouse *Mmp19* | Forward primer: GCTGACATTCGCCTCTCTTTC<br>Reverse primer: CACTCCTTGATAGGTCCCCTC |

**Supplementary Table 4. Antibody information in this study.**

| Antibody | Brand | Apply |
|---|---|---|
| Rabbit anti-MMP13 | 1:100, Proteinch, 18165-1-AP | IHC |
| Rabbit anti-COL2A1 | 1:400; Rockland, 600-401-104S | IHC |
| Mouse anti-COL1A1 | 1:100; Santa Cruz Biotechnology, sc-293182 | IHC |
| Rabbit anti-ADAMTS4 | 1:100, ABclonal, A2525 | IHC |
| Mouse anti-NOS2 | 1:100; Santa Cruz Biotechnology, sc-7271 | IHC |
| Rabbit anti-DDX5 | Cell Signaling Technology, 9877s | IF (1:100)<br>RIP (1:100)<br>WB (1:1000) |
| Mouse anti-DNA G4 | 1:100, sigma, MABE1126 | IF |
| Anti-EGFP | 1:100, invitrogen, CAB4211 | IF |
| Rabbit anti-COL1A1 | 1:1000, Abcam, ab34710 | WB |
| Mouse anti-DDX5 | 1:100, Santa Cruz Biotechnology, sc-365164 | ChIP |

| | | |
|---|---|---|
| Rabbit anti-MMP3 | 1:500, abcam, ab52915 | WB |
| Mouse anti-GAPDH | 1:1000, Abmart, M20028 | WB |
| Rabbit anti-Actin | 1:1000, Abmart, T40001M | WB |
| Rabbit anti-COL2A1 | 1:1000, Abcam, ab34712 | WB |
| Rabbit anti-ADAMTS5 | 1:1000, Abcam, ab41037 | WB |

**Supplementary Table 5. Primer sequences used in PCR.**

| Gene | Primer | Apply |
|---|---|---|
| Ddx5 Flox:532 bp, WT:453 bp | P1: AATCTGGTCAGATGTTTGGGCTG<br>P2: CTGAGTTACAGCATGAAGAAGCCAC | Genotyping |
| Aggrecan -cre$^{ERT}$ Wt: 299 bp Mut : 200 bp | 19388: GTT ATA TTC CGG AGC CCA CA<br>19387: AAA AGC GAC AAG AAG ACA CCA<br>19388: AAAAGCGACAAGAAGACACCA<br>oIMR8619: CTCCAGACTGCCTTGGGA AAA | Genotyping |
| Mouse *Fn1* | Forward primer: GTTACCCTTCCACACCCCAA<br>Reverse primer: ATGGCGTAATGGGAAACCGT | RIP-PCR |
| Mouse *Plod2* | Forward primer: GAGAGGCGGTGATGGAATGAA<br>Reverse primer: ACTCGGTAAACAAGATGACCAGA | RIP-PCR |
| Mouse *Ddx5* | Forward primer: TGTGATTGCAAGGCAGAACTTT<br>Reverse primer: TGGCCAGCCCTGAGCTT | PCR |
| Mouse *Gapdh* | Forward primer: AGGTCGGTGTGAACGGATTTG<br>Reverse primer: TGTAGACCATGTAGTTGAGGTCA | PCR |
| Mouse *Fn1-AS-SE25* 596/323 | Forward primer: GAGAGGAGCACTACCCCAGA<br>Reverse primer: GAGTAGCGCACCAAGAGGTT | PCR |
| Mouse *Plod2-AS-SE 14* 404/341 | Forward primer: GGAGCACTGAGTCCTGATGG<br>Reverse primer: TTTTCCTTCCAATCCACGGG | PCR |

**Supplementary Table 6. shRNA information**

| Gene | Sequence (5′→ 3′) | |
|---|---|---|
| Mouse *Ddx5* | 5'-CCGGCCTGGAAGACTGATTGACTTTCTCGAGA AAGTCAATCAGTCTTCCAGGTTTTTG-3' | shRNA |
| Mouse *Fn1-AS-WT-1* | 5'-CCGGGGGTACCGAATCACAGTAGTTCTCGAG AACTACTGTGATTCGGTACCCTTTTTG-3' | shRNA |
| Mouse *Plod2-AS-WT*-3 | 5'-CCGGCCCAAAGGGTGTGTTTATGTACTCGAGT ACATAAACACACCCTTTGGGTTTTTG-3' | shRNA |

**Supplementary Table 7. siRNA information**

| Gene | Sequence (5′→ 3′) | |
|---|---|---|
| Mouse *Fn1-AS-WT-1* | Sense: GGGUACCGAAUCACAGUAGUUTT<br>Antisense: AACUACUGUGAUUCGGUACCCTT | siRNA |
| Mouse *Fn1-AS-WT-2* | Sense: GCGUUAUCACUCUCAUUAATT<br>Antisense: UUAAUGAGAGUGAUAACGCTT | siRNA |
| Mouse *Fn1-AS-WT-3* | Sense: CAGUGUUUACACUGUCAAATT<br>Antisense: UUUGACAGUGUAAACACUGTT | siRNA |
| Mouse *Plod2-AS-WT-1* | Sense: CAAUGCUAGAGAUAUGACCUUTT<br>Antisense: AAGGUCAUAUCUCUAGCAUUGTT | siRNA |
| Mouse *Plod2-AS-WT-2* | Sense: CCUACUCCGGAAACAUUCCAATT<br>Antisense: UUGGAAUGUUUCCGGAGUAGGTT | siRNA |
| Mouse *Plod2-AS-WT-3* | Sense: CCCAAAGGGUGUGUUUAUGUATT<br>Antisense: TACAUAAACACACCCUUUGGG | siRNA |
| Mouse *Ddx5* | Sense: CCUGGAAGACUGAUUGACUUUTT<br>Antisense: AAAGUCAAUCAGUCUUCCAGGTT | siRNA |
