# Supplementary material for: DDX5 inhibits hyaline cartilage fibrosis and degradation in osteoarthritis via alternative splicing and G-quadruplex unwinding
Source: Nat Aging. 2024 May 17;4(5):664–80. doi: 10.1038/s43587-024-00624-0 (PMC11108786; doi:10.1038/s43587-024-00624-0)
Supplement: Supplementary file 2 — Reporting Summary [file 43587_2024_624_MOESM2_ESM.pdf]

nature portfolio

Corresponding author(s): Qiang Xu and Yang Sun

Last updated by author(s): Feb 14, 2024

Reporting Summary

Nature Portfolio wishes to improve the reproducibility of the work that we publish. This form provides structure for consistency and transparency in reporting. For further information on Nature Portfolio policies, see our Editorial Policies and the Editorial Policy Checklist.

Statistics

For all statistical analyses, confirm that the following items are present in the figure legend, table legend, main text, or Methods section.

n/a	Confirmed	
☐	☒	The exact sample size (*n*) for each experimental group/condition, given as a discrete number and unit of measurement
☐	☒	A statement on whether measurements were taken from distinct samples or whether the same sample was measured repeatedly
☐	☒	The statistical test(s) used AND whether they are one- or two-sided<br>*Only common tests should be described solely by name; describe more complex techniques in the Methods section.*
☐	☒	A description of all covariates tested
☐	☒	A description of any assumptions or corrections, such as tests of normality and adjustment for multiple comparisons
☐	☒	A full description of the statistical parameters including central tendency (e.g. means) or other basic estimates (e.g. regression coefficient) AND variation (e.g. standard deviation) or associated estimates of uncertainty (e.g. confidence intervals)
☐	☒	For null hypothesis testing, the test statistic (e.g. *F*, *t*, *r*) with confidence intervals, effect sizes, degrees of freedom and *P* value noted<br>*Give P values as exact values whenever suitable.*
☐	☒	For Bayesian analysis, information on the choice of priors and Markov chain Monte Carlo settings
☐	☒	For hierarchical and complex designs, identification of the appropriate level for tests and full reporting of outcomes
☐	☒	Estimates of effect sizes (e.g. Cohen's *d*, Pearson's *r*), indicating how they were calculated

Our web collection on statistics for biologists contains articles on many of the points above.

Software and code

Policy information about availability of computer code

Data collection

RNA-seq analysis
The shRNA-NC and shRNA-Ddx5 ATDC5 cells were treated with 5 ng/ml IL-1β and 25 ng/ml TNF-α (Peprotech) for 6h. 1 μg RNA per sample was used as input material for the RNA sample preparations. The PCR products were purified using the AMPure XP system and library quality was assessed on the Agilent Bioanalyzer 2100 system. RNA-seq was performed using an Illumina system, following the protocols -provided by Illumina for 2×150paired-end sequencing in WuXi NextCODE at Shanghai, China. Gene expression levels were quantified using FeatureCounts (v.1.6.3). Differential expression analysis was performed using the DESeq2 R package (1.20.0) and the resulting P values were adjusted using the Benjamini and Hochberg's approach for controlling the false discovery rate. Genes with an adjusted P value < 0.05 found by DESeq2 were classified as differentially expressed. GO and KEGG pathway enrichment analyses were performed by using the cluster Profiler R package (v.4.2.0). Clinical disease data are obtained from the GEO database (GSE114007). Upon analyszing of this dataset, significant differences were observed between two sequencing platforms (Illumina HiSeq 2000 and NextSeq 500). Considering the smaller intra-group differences of sequencing data obtained using the NextSeq 500 platform, we only selected this data for downstream analysis.
rMATS methods
The human raw data from the GEO public datasets (GSE114007) underwent quality controlled using trim galore (0.6.10) and were then mapped to the genome sequence (hg38) using STAR by rMATS software. The rMATS software (turbo_v4.2.0) was utilized for alternative splicing analysis (ASEs), which included the analysis of five splice types. The number of all identified ASEs, derived from GTF and the number of RNA is 110,965. The final output, which only included only reads that spanned defined junctions is 57,645. We then focused on all (SE).MATS.JC.txt, and among these ASEs, we identified 624 DASEs (PSI>4%, FDR< 0.05). The same parameters were used to RNA-seq raw data (GSE226983) from shRNA-NC and shRNA-Ddx5 ATDC5 cells treated with 5 ng/ml IL-1β and 25 ng/ml TNF-α. The genome file used mm39.fa and the GTF file was mm39.ncbiRefSeq.gtf. The AS events derived from GTF and the number of RNA is 94,101. The final output included 68,559 reads that span defined junctions. Then, we focused on all SE.MATS.JC.txt and among these ASEs, we identified 3262 DASEs (PSI>10%, FDR< 0.05). The rmats2sashimiplotl (v3.0.0) was used to produce plots with an annotation file and genomic coordinates of FN1 (Fn1) and

PLOD2 (Plod2) extracted from SE.MATS.JC.txt.
Single-cell sequencing data processing
Single-cell count matrices were obtained from GEO database (GSE104782) and converted to sparse matrices using the Seurat package (v4.0.4) in R. The 1600 individual chondrocytes obtained from the articular cartilage of 10 patients were put into following analysis. We used SCTransform to normalize gene expression and correct batch effects. Principal component analysis (PCA) was performed with filtered variable genes using RunPCA in the Seurat package. The first 30 principal components (PCs) were selected for Uniform Manifold Approximation and Projection (UMAP), and Louvain clustering was performed using RunUMAP and FindNeighbors/FindClusters in the Seurat package, respectively. The Harmony algorithm (v1.0) can accurately integrate single-cell data from different technology platforms and batches. Thus, we integrated these cells from 10 patients by using RunHarmony. Cell types were determined according to the marker gene expressions.
TMT-labeled proteomics assay
The shRNA-NC and shRNA-Ddx5 ATDC5 cells were treated with 5 ng/ml IL-1β and 25 ng/ml TNF-α (Peprotech) for 24h. The detailed steps cell treatment were as previously described62. The cells were washed by cold PBS buffer three times and then lysed with lysis buffer (8 M urea, 50 mM NH4HCO3, 1× protease and phosphatase inhibitors (Roche)) on ice for 30 min. Then the lysate was sonicated on ice for 3 min (2 sec on and 5 sec off) with 30% energy. The solution was centrifuged at 12000 rpm for 10 min and then transferred to a new EP tube. The concentration of extracted proteins was detected by using BCA assay. Then the proteins were reduced with 5 mM DTT at 56°C for 30 min and alkylated with 15 mM IAA in the dark at room temperature for 30 min. The alkylation reaction was quenched with 20 mM IAA at room temperature for 30 min. The concentration of urea in protein solution was diluted by adding 4-fold dilution with 100 mM NH4HCO3. Then the proteins were digested by adding sequencing grade trypsin (Hualishi) at the enzyme-to-protein ration of 1:50 (w/w) at 37°C for overnight. The tryptic-digestion peptides were desalted by Sep-Pak tC18 column and vacuum-dried by Speed-Vac before 12-plex TMT labeling. 12-plex TMT labeling was performed according to the manual description. The TMT labeling was performed according to the previously reported63. After labeling, all the TMT-labeled samples were pooled at 1:1 (w/w) ratio and vacuum-centrifuged to dry. The TMT-labeled peptides mixture was desalted by Sep-Pak C18 columns. TMT-labeling efficiency was analyzed before did deep proteome profiling. 10 μg TMT-labeled peptides were extracted and analyzed by Easy-nanoLC 1200 system tandem with Q Eaxactive HF-X mass spectrometer (Thermo Scientific, USA). All the dried samples were dissolved in loading buffer (0.1% formic acid in water, v/v) and then detected by Easy-nanoLC 1200 system tandem with Q Eaxactive HF-X mass spectrometer. For the deep proteome profiling analysis, all the mass spectrometry data were searched against UniProt mouse database (version 20180930) through Proteome Discoverer software (2.5) with the SEQUEST HT search engine. Enzyme specificity was set to trypsin. The 12-plex TMT label on lysine and peptide N-termini, carbamidomethylation of cysteine were set as fixed modifications. The acetylation of protein N-term and oxidation of methionine were set as variable modifications. The maximum missed cleavages were set at 2. The tolerances of MS and MS/MS were set at 20 ppm and 0.02 Da, respectively. The Percolator algorithm in PD was adopted to control peptide spectrum matches at a false discovery rate (FDR) and maximum delta Cn=0.05. The FDR of protein and peptide identification were all set to 1%. The proteins in treated group with 1.2 fold-change were filtered. The statistical analysis were used Student's t-test and the p-value was cut-off at 0.05.

Data analysis

RNA-seq analysis
The shRNA-NC and shRNA-Ddx5 ATDC5 cells were treated with 5 ng/ml IL-1β and 25 ng/ml TNF-α (Peprotech) for 6h. 1 μg RNA per sample was used as input material for the RNA sample preparations. The PCR products were purified using the AMPure XP system and library quality was assessed on the Agilent Bioanalyzer 2100 system. RNA-seq was performed using an Illumina system, following the protocols -provided by Illumina for 2×150paired-end sequencing in WuXi NextCODE at Shanghai, China. Gene expression levels were quantified using FeatureCounts (v.1.6.3). Differential expression analysis was performed using the DESeq2 R package (1.20.0) and the resulting P values were adjusted using the Benjamini and Hochberg's approach for controlling the false discovery rate. Genes with an adjusted P value < 0.05 found by DESeq2 were classified as differentially expressed. GO and KEGG pathway enrichment analyses were performed by using the cluster Profiler R package (v.4.2.0). Clinical disease data are obtained from the GEO database (GSE114007). Upon analyszing of this dataset, significant differences were observed between two sequencing platforms (Illumina HiSeq 2000 and NextSeq 500). Considering the smaller intra-group differences of sequencing data obtained using the NextSeq 500 platform, we only selected this data for downstream analysis.
rMATS methods
The human raw data from the GEO public datasets (GSE114007) underwent quality controlled using trim galore (0.6.10) and were then mapped to the genome sequence (hg38) using STAR by rMATS software. The rMATS software (turbo_v4.2.0) was utilized for alternative splicing analysis (ASEs), which included the analysis of five splice types. The number of all identified ASEs, derived from GTF and the number of RNA is 110,965. The final output, which only included only reads that spanned defined junctions is 57,645. We then focused on all (SE).MATS.JC.txt, and among these ASEs, we identified 624 DASEs (PSI>4%, FDR< 0.05). The same parameters were used to RNA-seq raw data (GSE226983) from shRNA-NC and shRNA-Ddx5 ATDC5 cells treated with 5 ng/ml IL-1β and 25 ng/ml TNF-α. The genome file used mm39.fa and the GTF file was mm39.ncbiRefSeq.gtf. The AS events derived from GTF and the number of RNA is 94,101. The final output included 68,559 reads that span defined junctions. Then, we focused on all SE.MATS.JC.txt and among these ASEs, we identified 3262 DASEs (PSI>10%, FDR< 0.05). The rmats2sashimiplotl (v3.0.0) was used to produce plots with an annotation file and genomic coordinates of FN1 (Fn1) and PLOD2 (Plod2) extracted from SE.MATS.JC.txt.
Single-cell sequencing data processing
Single-cell count matrices were obtained from GEO database (GSE104782) and converted to sparse matrices using the Seurat package (v4.0.4) in R. The 1600 individual chondrocytes obtained from the articular cartilage of 10 patients were put into following analysis. We used SCTransform to normalize gene expression and correct batch effects. Principal component analysis (PCA) was performed with filtered variable genes using RunPCA in the Seurat package. The first 30 principal components (PCs) were selected for Uniform Manifold Approximation and Projection (UMAP), and Louvain clustering was performed using RunUMAP and FindNeighbors/FindClusters in the Seurat package, respectively. The Harmony algorithm (v1.0) can accurately integrate single-cell data from different technology platforms and batches. Thus, we integrated these cells from 10 patients by using RunHarmony. Cell types were determined according to the marker gene expressions.
Bi-omics joint analysis
DESeq was used to perform differential analysis on both RNA-seq and protein-seq data and the function inner_join (dplyr version 1.1.2) is used to combine the two sets of data based on the same differential gene. The ggplot2 is used to display key genes in four quadrants. The omics joint display of GSEA analysis also adopted a similar approach. The pathway enrichment was performed using KEGG on both sets of data, and the function inner_join was used to combine the two sets of data based on the same pathways. The KEGG pathways with enrichment score > 0 in both sets of data were screened out.
Quantitative real-time PCR
Gapdh mRNA levels were used as an internal control for the target mRNAs. Normalization and fold changes were calculated using the ΔΔCt method.
Statistical Analysis
The sample size for each experiment was determined based on our previous experience. Animals used in the experiments of this study were randomly grouped. IF and histology were performed and analyzed in a double-blinded manner. Statistical analyses were completed using the Prism GraphPad. Two-tailed unpaired or paired Student's t test (two groups) and one-way ANOVA (multiple groups), followed by Tukey's post

hoc test were used. The non-parametric data was transformed prior to parametric statistical analyses. The results were expressed as the mean ± standard deviation (SEM), as indicated in the Figure Legends. Differences with P<0.05 were considered statistically significant, and ns represents no significance.

For manuscripts utilizing custom algorithms or software that are central to the research but not yet described in published literature, software must be made available to editors and reviewers. We strongly encourage code deposition in a community repository (e.g. GitHub). See the Nature Portfolio guidelines for submitting code & software for further information.

Data

Policy information about availability of data

All manuscripts must include a data availability statement. This statement should provide the following information, where applicable:

- Accession codes, unique identifiers, or web links for publicly available datasets
- A description of any restrictions on data availability
- For clinical datasets or third party data, please ensure that the statement adheres to our policy

Data availability
The raw sequence data reported in the present paper have been deposited in the GEO database under accession no. GSE226983. The proteomics raw data have been deposited to a member of the ProteomeXchange consortium iProX (http://www.iprox.org) with the project ID IPX0006326000 (URL: https://www.iprox.cn/page/PSV023.html;?url=1682066289366fm6j, Password: Of2X). The analysis code for the article have been uploaded to the associated analysis website: https://github.com/Portulaca666/AS.code. All data supporting the findings for this study are available within the paper or from the corresponding authors upon reasonable request. All data is already publicly available.

Research involving human participants, their data, or biological material

Policy information about studies with human participants or human data. See also policy information about sex, gender (identity/presentation), and sexual orientation and race, ethnicity and racism.

Reporting on sex and gender	We indiscriminately choose the sex and gender of osteoarthritis patients.
Reporting on race, ethnicity, or other socially relevant groupings	In the experiment, we collected cartilage tissue from patients with osteoarthritis in our respective regions after total knee replacement surgery, and indiscriminately selected race, ethics, or other socially relevant groups.
Population characteristics	We collected cartilage tissue from patients with osteoarthritis in our respective regions after total knee replacement surgery,and indiscriminately selected population characteristics. Due to the high incidence of osteoarthritis in the elderly population, the samples we collect are generally older than 60 years old<br>During the process of collecting clinical samples, articular cartilage was collected from OA patients undergoing total knee arthroplasty. Patients with osteoarthritis are judged based on symptoms and imaging standards. The artistic cartilage samples from OA patients were observed from Nanjing Drum Tower Hospital. We also listed the clinical and demographic characteristics of the study population
Recruitment	We collected cartilage tissue from patients with osteoarthritis in our respective regions after total knee replacement surgery. The attending physician, who is also the researcher, invites patients to participate in the study when they meet the research criteria, without any self-selection bias or other biases during the recruitment process
Ethics oversight	The articular cartilage samples from OA patients were obtained from Nanjing Drum Tower Hospital. The clinical and demographic characteristics of the study population sets are listed in Supplementary Table 2. This study was approved by the Ethical Committee of the Nanjing Drum Tower Hospital, which is Affiliated Hospital of Nanjing University Medical School (2020-156-01).

Note that full information on the approval of the study protocol must also be provided in the manuscript.

Field-specific reporting

Please select the one below that is the best fit for your research. If you are not sure, read the appropriate sections before making your selection.

☒ Life sciences ☐ Behavioural & social sciences ☐ Ecological, evolutionary & environmental sciences

For a reference copy of the document with all sections, see nature.com/documents/nr-reporting-summary-flat.pdf

Life sciences study design

All studies must disclose on these points even when the disclosure is negative.

Sample size	Patient samples are accustomed to using n = 6(PMID: 36564153), so in our experiment, we used a patient sample of n=6.<br>The sample size for animal model was determined based on our previous experience (PMID: 34569725) and other articles (eg. PMID: 32162789)

Data exclusions

The human raw data from the GEO public datasets (GSE114007) underwent quality controlled using trim galore (0.6.10) and were then mapped to the genome sequence (hg38) using STAR by rMATS software. The rMATS software (turbo_v4.2.0) was utilized for alternative splicing analysis (ASEs), which included the analysis of five splice types. The number of all identified ASEs, derived from GTF and the number of RNA is 110,965. The final output, which only included only reads that spanned defined junctions is 57,645. We then focused on all (SE).MATS.JC.txt, and among these ASEs, we identified 624 DASEs (PSI>4%, FDR< 0.05). The same parameters were used to RNA-seq raw data (GSE226983) from shRNA-NC and shRNA-Ddx5 ATDC5 cells treated with 5 ng/ml IL-1β and 25 ng/ml TNF-α. The genome file used mm39.fa and the GTF file was mm39.ncbiRefSeq.gtf. The AS events derived from GTF and the number of RNA is 94,101. The final output included 68,559 reads that span defined junctions. Then, we focused on all SE.MATS.JC.txt and among these ASEs, we identified 3262 DASEs (PSI>10%, FDR< 0.05). The rmats2sashimiplotl (v3.0.0) was used to produce plots with an annotation file and genomic coordinates of FN1 (Fn1) and PLOD2 (Plod2) extracted from SE.MATS.JC.txt.
In single-cell sequencing data processing, A cluster named ZC that with low RNA counts and low Feature counts can be filtered did not be token into account in following analysis.

Replication

We used three independent repeated experiments in our experiments.

Randomization

We adopt the principle of random group

Blinding

The investigators were blinded to group allocation during data collection and or analysis.

Reporting for specific materials, systems and methods

We require information from authors about some types of materials, experimental systems and methods used in many studies. Here, indicate whether each material, system or method listed is relevant to your study. If you are not sure if a list item applies to your research, read the appropriate section before selecting a response.

Materials & experimental systems

n/a	Involved in the study
☐	☒ Antibodies
☐	☒ Eukaryotic cell lines
☒	☐ Palaeontology and archaeology
☐	☒ Animals and other organisms
☒	☐ Clinical data
☒	☐ Dual use research of concern
☒	☐ Plants

Methods

n/a	Involved in the study
☒	☐ ChIP-seq
☒	☐ Flow cytometry
☒	☐ MRI-based neuroimaging

Antibodies

Antibodies used

Rabbit anti-MMP13, 1:100, Proteinch, 18165-1-AP IHC; Rabbit anti-COL2A1, 1:400; Rockland, 600-401-104S, IHC; Mouse anti-COL1A, 1:100; Santa Cruz Biotechnology, sc-293182, IHC; Rabbit anti-ADAMTS4,1:100, ABclonal, A2525, IHC; Mouse anti-NOS2,1:1000; Santa Cruz Biotechnology, sc-7271,WB; Rabbit anti-DDX5 Cell Signaling Technology, 9877s IF (1:100)RIP (1:100)WB (1:1000); Mouse anti-DNA G4,1:100, sigma, MABE1126, IF; anti-EGFP, 1:100, invitrogen, CAB4211, IF; Rabbit anti-COL1A1, 1:1000, Abcam, ab34710,WB; Mouse anti-DDX5 1:100, Santa Cruz Biotechnology, sc-365164 ChIP; Rabbit anti-MMP3, 1:500, abcam, ab52915, WB; Mouse anti-GAPDH,1:1000, Abmart, M2006,WB;Rabbit anti-Actin,1:1000, Abmart, T40001M, WB; Rabbit anti-ADAMTS5, 1:1000, Abcam, ab41037; Rabbit anti-COL2A1, 1:1000; Abcam,, ab34712, WB.

Validation

Rabbit anti-MMP13, 1:100, Proteinch, 18165-1-AP
https://app.ptgcn.com/online/AbDataSheet.htm?catno=18165-1-AP

Rabbit anti-COL2A1, 1:400; Rockland, 600-401-104S,
https://www.rockland.com/categories/primary-antibodies/collagen-type-ii-antibody-600-401-104-0.1/

Mouse anti-COL1A, 1:100; Santa Cruz Biotechnology, sc-293182
https://www.scbt.com/p/col1a1-antibody-3g3?requestFrom=search

Rabbit anti-ADAMTS4,1:100, ABclonal, A2525,
https://abclonal.com.cn/catalog/A2525

Mouse anti-NOS2,1:1000; Santa Cruz Biotechnology, sc-7271
https://www.scbt.com/p/nos2-antibody-c-11?requestFrom=search

Rabbit anti-DDX5 Cell Signaling Technology, 9877s
https://www.cellsignal.cn/products/primary-antibodies/ddx5-d15e10-xp-rabbit-mab/9877

Mouse anti-DNA G4,1:100, sigma, MABE1126
https://www.sigmaaldrich.cn/CN/zh/search/mabe1126?focus=products&page=1&perpage=30&sort=relevance&term=MABE1126&type=product

Rabbit anti-EGFP, 1:100, invitrogen, CAB4211

https://www.thermofisher.cn/cn/zh/antibody/product/eGFP-Antibody-Polyclonal/CAB4211

Rabbit anti-COL1A1, 1:1000, Abcam, ab34710
https://www.abcam.cn/products/primary-antibodies/collagen-i-antibody-ab34710.html

Mouse anti-DDX5 1:100, Santa Cruz Biotechnology, sc-365164
https://www.scbt.com/p/p68-rna-helicase-antibody-d-7?requestFrom=search

Rabbit anti-MMP3, 1:500, abcam, ab52915
https://www.abcam.cn/products/primary-antibodies/mmp3-antibody-ep1186y-ab52915.html

Mouse anti-GAPDH,1:1000, Abmart, M2006
http://www.ab-mart.com.cn/page.aspx?node=%2059%20&id=%20984

Rabbit anti-Actin,1:1000, Abmart, T40001M
http://www.ab-mart.com.cn/page.aspx?node=%2059%20&id=%201021

Rabbit anti-ADAMTS5, 1:1000, Abcam, ab41037
https://www.abcam.cn/products/primary-antibodies/adamts5-antibody-ab41037.html

Rabbit anti-COL2A1, 1:1000; Abcam, ab34712
https://www.abcam.cn/products/primary-antibodies/collagen-ii-antibody-ab34712.html

Eukaryotic cell lines

Policy information about cell lines and Sex and Gender in Research

Cell line source(s)	The ATDC5 cell line is derived from Shanghai biowing applied biotechnology co.ltd
Authentication	The cell identification result of this strain is a mouse cell line, and the STR typing result is consistent with the ATDC-5 genotype of the control mouse cell line, and the STR typing result is completely matched. No multiple alleles were found in the cell line during this test, and there was no cross contamination or source contamination
Mycoplasma contamination	No mycoplasma contamination has been detected.
Commonly misidentified lines (See ICLAC register)	Common misidentification cell lines were not used in the study

Animals and other research organisms

Policy information about studies involving animals; ARRIVE guidelines recommended for reporting animal research, and Sex and Gender in Research

Laboratory animals	Mice were housed with a 12 h light/dark cycle (light: 7:00 to 19:00), with 55% relative humidity at 22 °C, and with free access to food and water.<br>SPF mice were bred and assigned for DMM surgery at week 12 after birth, and samples are taken from the mice at week 18 or 24.
Wild animals	No wild animals were used in the study.
Reporting on sex	Due to the impact of estrogen on this model, OA models were conducted on male mice.
Field-collected samples	No field collected samples were used in the study.
Ethics oversight	All animal experiments were carried out in accordance with the NIH Guide for the Care and Use of Laboratory Animals (National Academies Press, 2011) and were approved by the Experimental Animal Care and Use Committee of Nanjing University (IACUC-2210005).

Note that full information on the approval of the study protocol must also be provided in the manuscript.
